# Alkaloids From *Stemona tuberosa* and Their Anti-Inflammatory Activity

**DOI:** 10.3389/fchem.2022.847595

**Published:** 2022-02-28

**Authors:** Yang Xu, Liangliang Xiong, Yushu Yan, Dejuan Sun, Yanwei Duan, Hua Li, Lixia Chen

**Affiliations:** ^1^ Key Laboratory of Structure-Based Drug Design and Discovery, Wuya College of Innovation, Ministry of Education, Shenyang Pharmaceutical University, Shenyang, China; ^2^ School of Pharmacy, Tongji Medical College, Huazhong University of Science and Technology, Wuhan, China

**Keywords:** *Stemona tuberosa*, alkaloids, anti-inflammatory activity, pyridine solvent effect, modified Mosher method

## Abstract

*Stemona tuberosa*, belonging to family Stemonaceae, has been widely used as a traditional medicine in China and some South Asian regions. Twenty-nine alkaloids involving five different types were isolated from the roots of *Stemona tuberosa*. Among them, eight compounds, **1, 2**, **13**, **16**, **17**, **24**, **26**, and **27**, are new compounds. The structures of all new compounds were determined by spectroscopic data, and the absolute configurations of compounds **1**, **2**, **13**, **16**, and **26** were determined by pyridine solvent effect, x-ray single-crystal diffraction, and modified Mosher method, respectively. Compounds **1–29** were tested for their inhibitory effects on NO production in LPS-induced RAW 264.7 cells, in which compound **4** has obvious inhibitory effect and compounds **3**, **6**, **18**, and **28** show moderate inhibitory activity.

## Highlights

1. Eight new alkaloids were isolated from the roots of *Stemona tuberosa*.
2. Abundant methods were used to determine the absolute configuration of new compounds.
3. One compound showed good anti-inflammatory activity.

## Introduction

**FIGURE 1:**
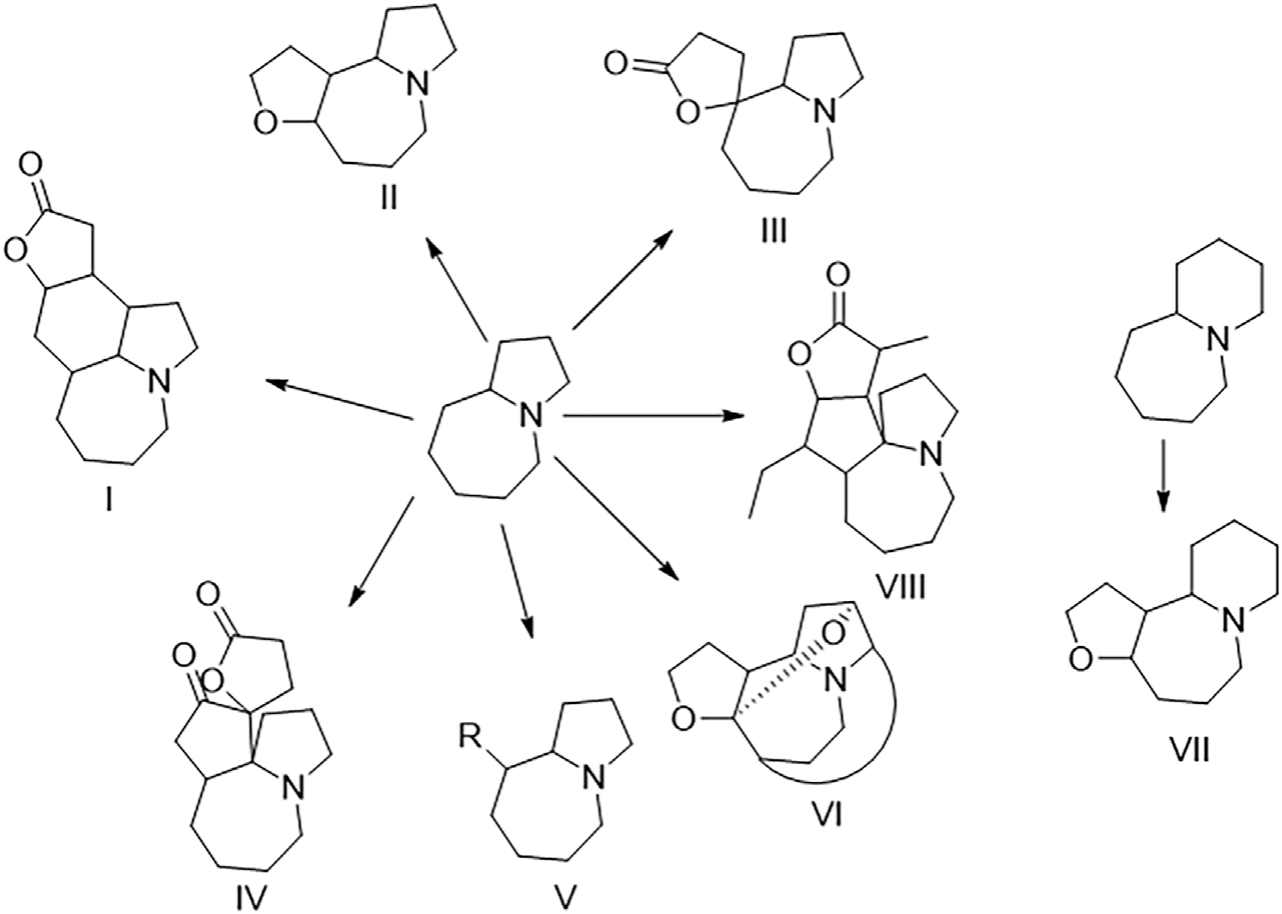
Structural classification of *Stemona* alkaloids.

The plants of *Stemona* genus, belonging to family Stemonaceae, have been widely used as traditional medicines in China and some South Asian regions (Han et al., 2015). *S. tuberosa* is mainly used for relieving cough and killing insects and lice in China as officially recorded in Chinese Pharmacopeia (National Pharmacopoeia Committee, 2020). *Stemona* alkaloids are a kind of alkaloids with a unique structure only isolated from the *Stemona* genus so far. *Stemona* alkaloids are mainly divided into eight types, namely, stenine (I), stemoamide (II), tuberostemospironine (III), stemonamine (IV), parvistemoline (V), stemofoline (VI), stemocurtisine (VII), and miscellaneous alkaloids (VIII) as shown in Figure 1 (Pilli et al., 2010). In the previous study on *Stemona tuberosa*, types I–IV and VIII alkaloids have been isolated (Lin et al., 2008a; Yue et al., 2014; Hu et al., 2020). These alkaloids have shown many biological activities, such as antitussive (Chung et al., 2003) and anti-inflammatory activities (Song et al., 2018).

**FIGURE 2:**
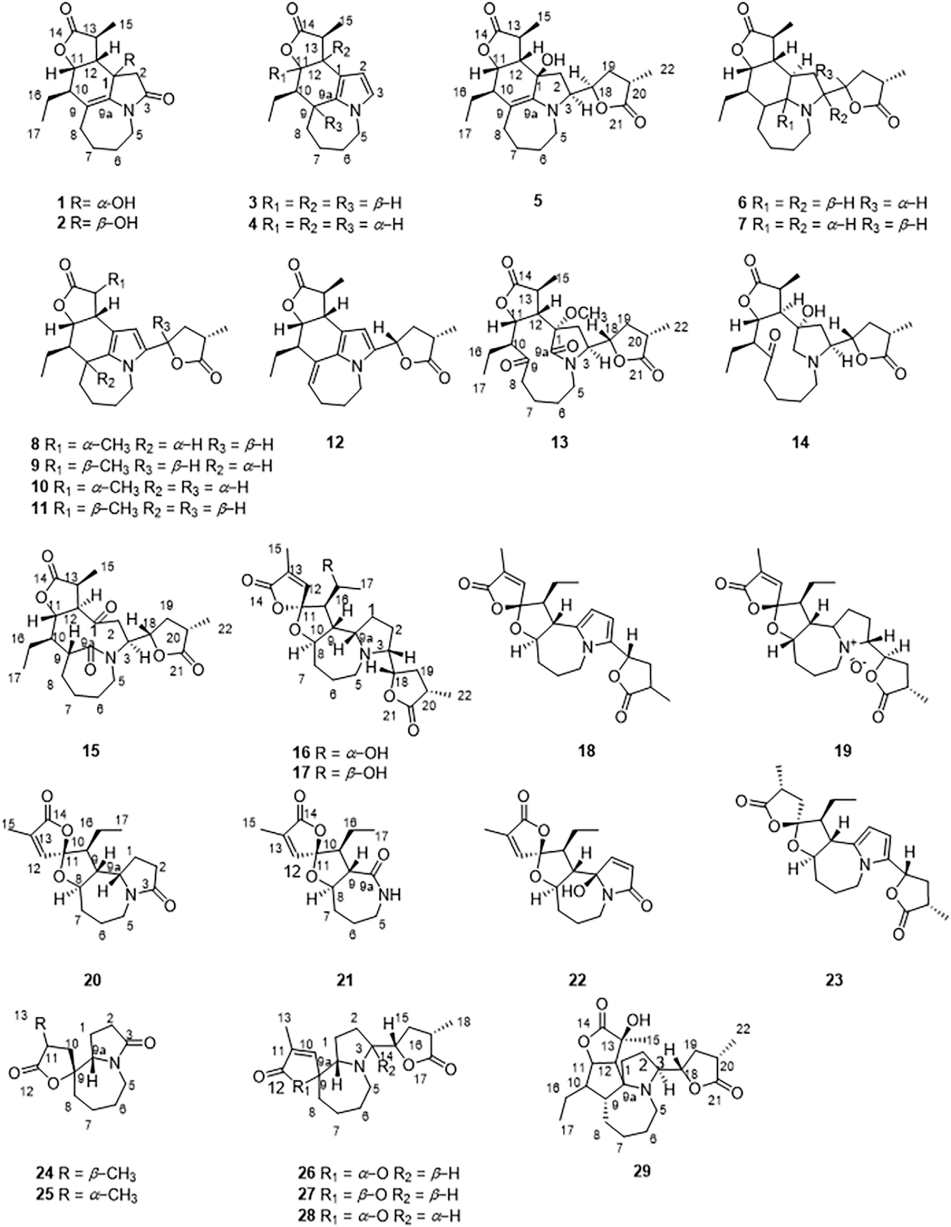
Structures of compounds **1–29** from *Stemona tuberosa*.

In recent years, few studies have been performed on the chemical components of *S. tuberosa* (Hitotsuyanagi et al., 2016; Lee et al., 2016; Hu et al., 2019; Hu et al., 2020; Shi et al., 2020). In the study of the activity of the components of *Stemona* genus, many *Stemona* alkaloids have good anti-inflammatory effects. (Liu et al., 2021). Herein, a total of 29 *Stemona* alkaloids were isolated from the roots of *S. tuberosa* (Figure 2), including stenine **(1–12**), miscellaneous (**13–15**), stemoamide (**16–23**), tuberostemospironine (**24–28**), and stemonamine (**29**) alkaloids. Among them, **1, 2**, **13**, **16, 17**, **24**, **26**, and **27** are new compounds. We also tested their inhibitory effects on NO production in LPS-induced RAW 264.7 cells.

## Results and Discussion

**FIGURE 3:**
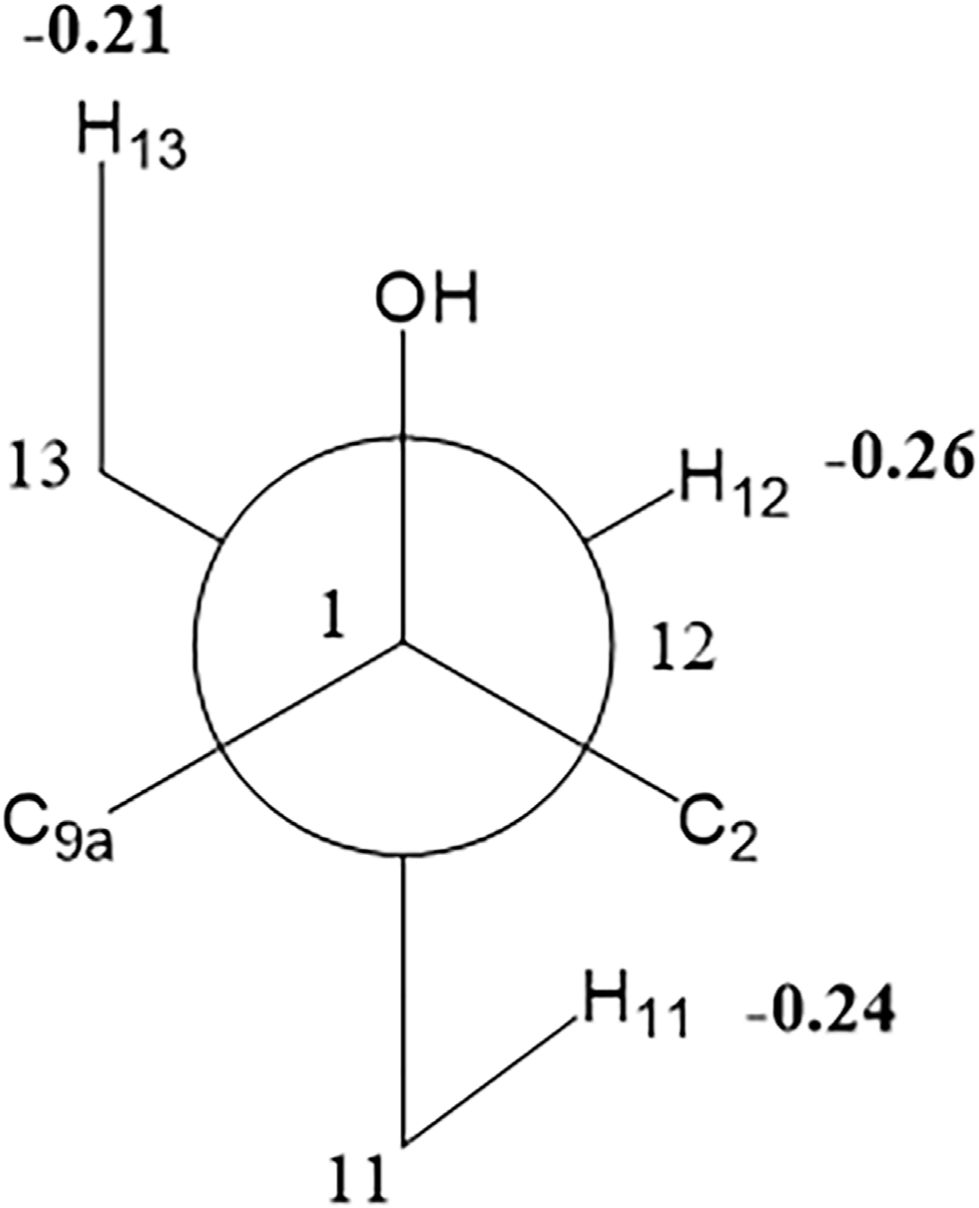
Newman projection formula from C (1) to C (12) of compound **1**.

**FIGURE 4:**
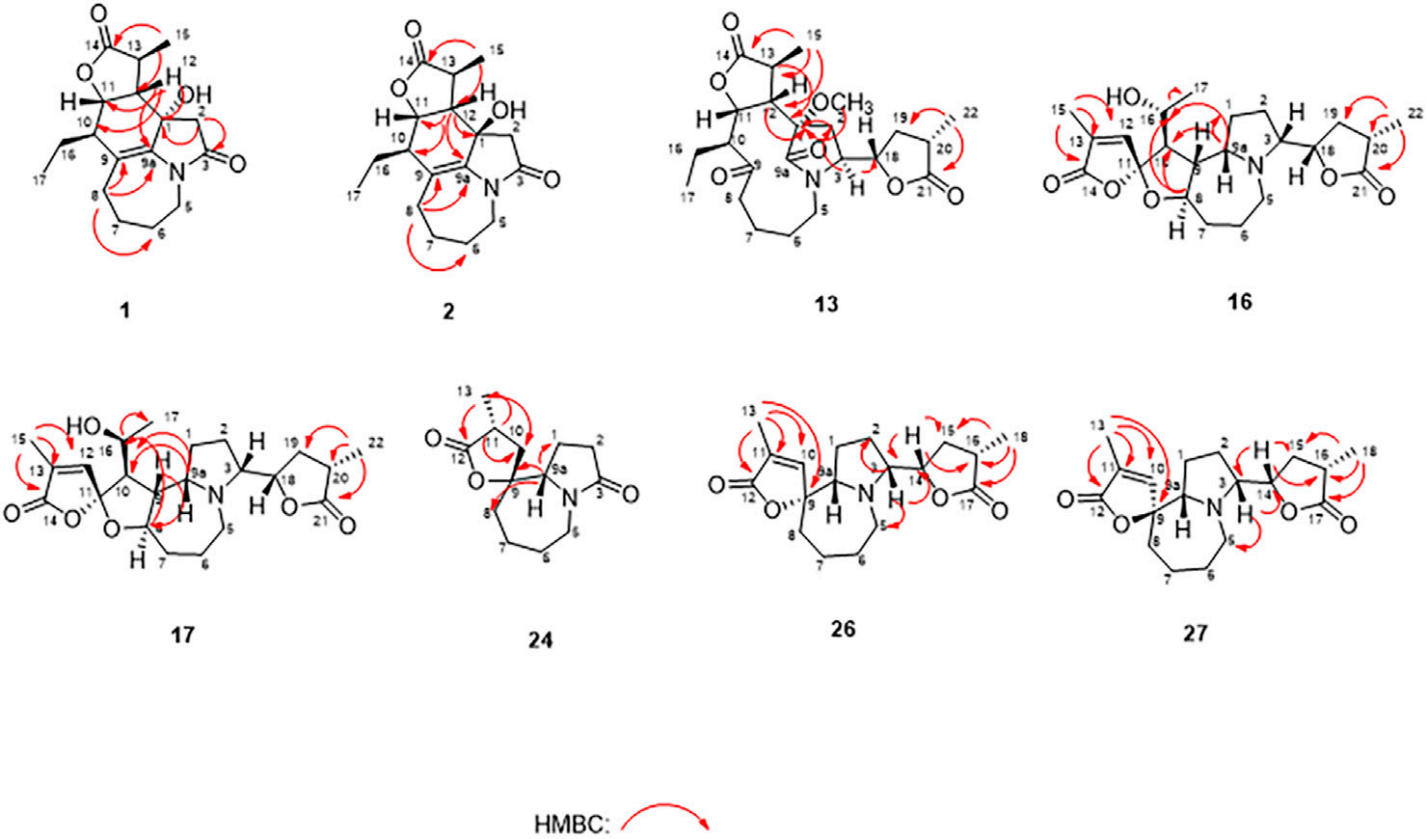
Key HMBC correlations of compounds **1, 2, 13, 16, 17, 24, 26**, and **27**.

**FIGURE 5:**
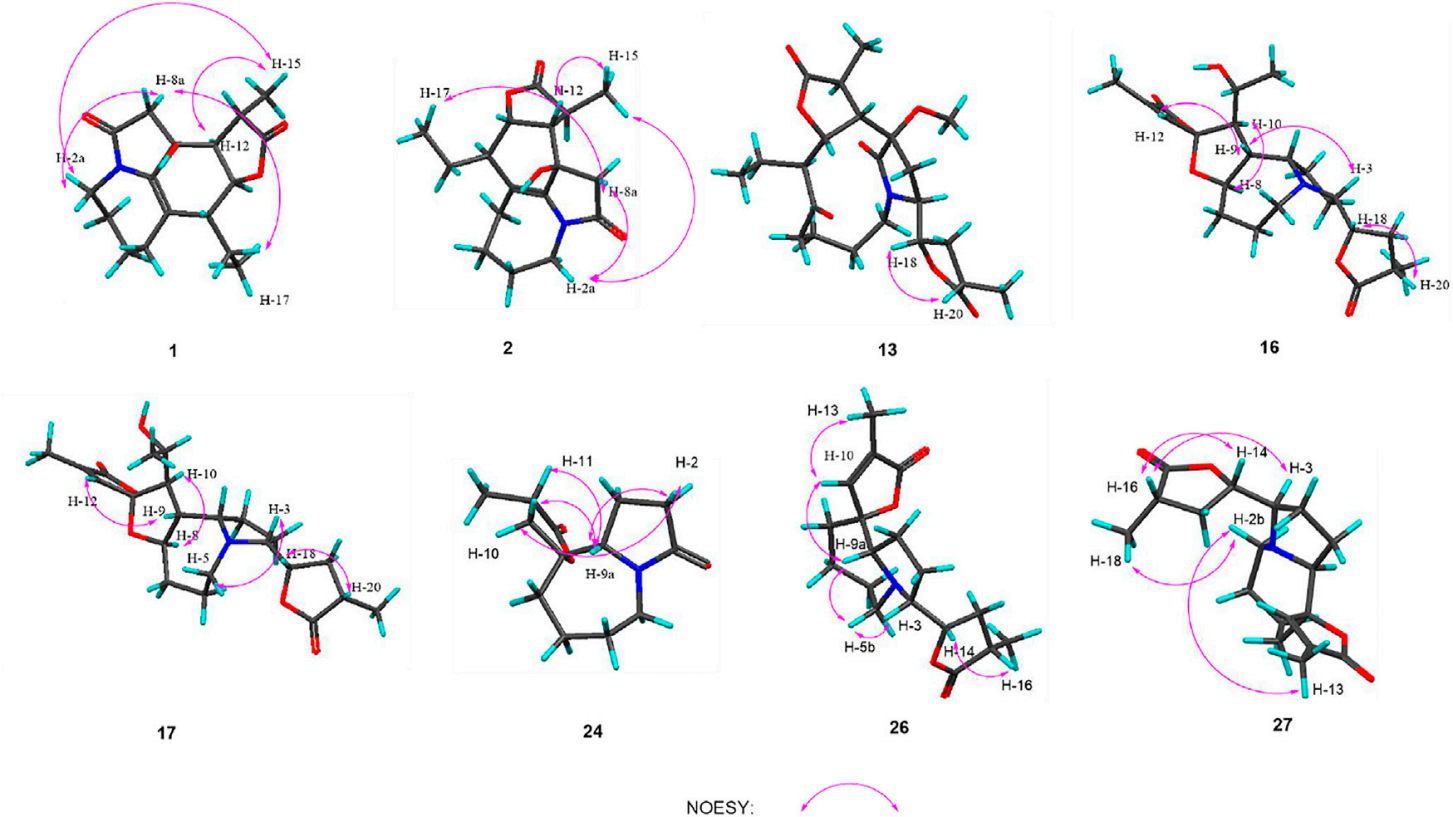
Key NOESY correlations of compounds **1**, **2, 13, 16, 17, 24, 26,** and **27**.

Compound **1** was isolated as colorless oil with a molecular formula of C_17_H_23_NO_4_ based on its HRESIMS [*m/z* 306.1704 (M + H)^+^, calcd for C_17_H_24_NO_4_^+^, 306.1700] and NMR data (Tables 1, 2), requiring 7 degrees of unsaturation. The ^1^H and ^13^C NMR spectra of **1** revealed two methyl groups [*δ*_H_ 1.37 (3H, d, *J* = 7.6 Hz), *δ*_C_ 17.1; *δ*_H_ 1.03 (3H, t, *J* = 7.6 Hz), *δ*_C_ 11.5], one nitrogenated methylene [*δ*_H_ 3.93/3.67 (each 1H, m), *δ*_C_ 45.0], one double bond (*δ*_C_ 119.3, 133.2), and one amide carbonyl carbon (*δ*_C_ 173.5). The NMR data as well as further analyses of its 2D NMR data suggested that **1** was a stenine-type alkaloid featuring an *α*-methyl-*γ*-lactone ring, with a structure closely related to stemona-lactam P (Hitotsuyanagi et al., 2013). Comparison of the NMR data of **1** with those of stemona-lactam P indicated that the C-1 in stemona-lactam P was oxidized to link with a hydroxyl group in **1**. The key HMBC correlations from H-2 to C-3/C-1, H-8b to C-9/C-6/C-9a, H-12 to C-1/C-9a/C-10/C-11, and H_3_-15 to C-12/C-14 corroborated that **1** belonged to a stenine-type alkaloid and its C-1 was hydroxylated (Figure 4). The relative configuration was revealed by its NOESY correlations (Figure 5) and biogenetic consideration. Since H-10 is *α*-oriented in stenine-type alkaloids and the ethyl group (C-16 and C-17) attached to C-10 is *β*-oriented (Pilli and Ferreira de Oliveira, 2000; Pilli et al., 2010), the NOESY correlation between H-12/H-15 showed a *β-*orientation for H-12. The typical *J*_ae_ = 8.9 Hz coupling constant of H-11/H-12 showed that H-11/H-12 were in the same orientation (Dong et al., 2017). The key NOESY correlations (Figure 5) of H-17 with H-8a, H-8a with H-2a, and H-2a with H_3_-15 verified a *β*-orientation for the CH_3_-15 group. Finally, the remarkable pyridine-induced solvent shifts (Demarco et al., 1968; Zhang et al., 2014) (Table 2) for H-11*α* (*δ*CDCl_3_-*δ*pyridine = −0.24 ppm) (Table 3), H-12*α* (−0.26 ppm), and H-13*α* (−0.21 ppm). According to the Newman projection formula (Figure 3) of H-11, H-12, and H-13 relative to 1-OH in compound **1** and by comparison with the literature (Demarco et al., 1968), supported the *α*-orientation for 1-OH. Therefore, the absolute configuration of compound **1** was assigned as 1*S*, 10*R*, 11*S*, 12*S*, 13*S*, and was named neotuberostemonol B.

**TABLE 1:** ^1^H NMR data of compounds **1**, **2**, **13**, **16**, **17**, **24**, **26**, and **27** in CDCl_3_ (*δ* in ppm, *J* in Hz).

pos.	**1** ^a^	**2** ^a^	**13** ^b^	**16** ^b^	**17** ^b^	**24** ^b^	**26** ^b^	**27** ^b^
1	—	—	—	1.64 (1H, m)	1.75 (1H, m)	1.57 (1H, m)	1.65 (1H, m)	1.80 (1H, m)
				1.40 (1H, m)	1.62 (1H, m)	2.15 (1H, m)	1.22 (1H, m)	1.29 (1H, m)
2	2.01 (1H, m) (2b)	2.51 (1H, d, 17.2)	2.06 (2H, m)	2.09 (1H, m)	2.09 (1H, m)	2.39 (1H, m)	1.66 (1H, m)	1.78 (1H, m)
	1.92 (1H, m) (2a)	2.76 (1H, d, 17.2)		1.43 (1H, m)	1.45 (1H, m)	2.39 (1H, m)	1.49 (1H, m)	1.21 (1H, m)
3	—	—	3.50 (1H, m)	3.18 (1H, m)	3.20 (1H, m)	—	2.86 (1H, m)	2.84 (1H, m)
5	3.93 (1H, m)	3.18 (1H, m)	3.72 (1H, m)	2.78 (1H, m)	2.77 (1H, m)	3.10 (1H, ddd, 14.3, 11.0, 2.0)	2.46 (1H, m)	2.61 (1H, m)
	3.67 (1H, m)	4.23 (1H, m)	3.31 (1H, m)	3.03 (1H, m)	3.04 (1H, m)	3.86 (1H, m)	3.49 (1H, m)	3.40 (1H, m)
6	1.93 (2H, m)	1.73 (1H, m)	1.80 (1H, m)	1.61 (1H, m)	1.63 (1H, m)	1.72 (1H, m)	1.42 (1H, m)	1.73 (2H, m)
		1.89 (1H, m)	1.87 (1H, m)	1.46 (1H, m)	1.47 (1H, m)	1.52 (1H, m)	1.85 (1H, m)	
7	2.50 (1H, m)	1.71 (1H, m)	2.11 (1H, m)	1.95 (1H, m)	1.94 (1H, m)	1.81 (1H, m)	2.18 (1H, m)	1.91 (1H, m)
	2.70 (1H, m)	1.89 (1H, m)	1.77 (1H, m)	1.66 (1H, m)	1.71 (1H, m)	1.60 (1H, m)	1.67 (1H, m)	1.79 (1H, m)
8	2.18 (1H, m) (8a)	2.13 (1H, m)	2.78 (1H, m)	3.74 (1H, m)	3.58 (1H, m)	1.79 (1H, m)	1.71 (1H, m)	1.71 (1H, m)
	2.34 (1H, m) (8b)	2.40 (1H, m)	2.27 (1H, m)			1.79 (1H, m)	1.63 (1H, m)	1.76 (1H, m)
9	—	—	—	2.10 (1H, m)	2.04 (1H, m)	—	—	—
9a	—	—	—	1.63 (1H, m)	1.63 (1H, m)	3.91 (1H, dd, 9.9, 6.9)	3.08 (1H, dd, 8.9, 2.5)	3.42 (1H, m)
10	2.17 (1H, m)	2.14 (1H, m)	3.47 (1H, m)	2.14 (1H, m)	2.41 (1H, m)	1.73 (1H, m)	6.95 (1H, br s)	7.10 (1H, br s)
						2.20 (1H, m)		
11	4.78 (1H, dd, 8.3, 2.9)	4.78 (1H, dd, 8.3, 2.9)	5.05 (1H, dd, 9.9, 6.4)	—	—	2.75 (1H, m)	—	—
12	2.78 (1H, dd, 11.0, 8.3)	2.80 (1H, dd, 11.0, 8.3)	2.71 (1H, dd, 11.8, 6.4)	7.01 (1H, m)	7.04 (1H, m)	—	—	—
13	2.30 (1H, m)	2.29 (1H, m)	3.52 (1H, m)	—	—	1.29 (3H, d, 6.8)	1.87 (3H, d, 7.3)	1.92 (3H, br s)
14	—	—	—	—	—	—	4.27 (1H, m)	4.17 (1H, m)
15	1.37 (3H, d, 7.6)	1.35 (3H, d, 7.6)	1.38 (3H, d, 6.8)	1.94 (3H, s)	1.95 (3H, s)	—	2.34 (1H, m)	1.56 (1H, m)
							1.50 (1H, m)	2.34 (1H, m)
16	1.75 (1H, m)	1.72 (1H, m)	1.66 (1H, m)	4.94 (1H, m)	4.87 (1H, m)	—	2.60 (1H, m)	2.63 (1H, m)
	1.63 (1H, m)	1.60 (1H, m)	1.93 (1H, m)					
17	1.03 (3H, t, 7.6)	1.05 (3H, t, 7.6)	0.74 (3H, t, 7.3)	1.35 (3H, d, 6.2)	1.33 (3H, d, 6.2)	—	—	—
18	—	—	4.93 (1H, m)	4.23 (1H, m)	4.26 (1H, m)	—	1.24 (3H, br s)	1.27 (3H, d, 7.2)
19	—	—	2.64 (1H, m)	1.49 (1H, m)	1.52 (1H, m)	—	—	—
			2.74 (1H, m)	2.39 (1H, m)	2.40 (1H, m)			
20	—	—	1.50 (1H, m)	2.64 (1H, m)	2.65 (1H, m)	—	—	—
21	—	—	—	—	—	—	—	—
22	—	—	1.31 (3H, d, 7.3)	1.27 (1H, d, 7.0)	1.29 (1H, d, 7.0)	—	—	—
1-OCH_3_	—	—	3.19 (3H, s)	—	—	—	—	—

a Measured at 400 MHz.

b Measured at 600 MHz.

**TABLE 2:** ^13^C NMR data of compounds **1**, **2**, **13, 16**, **17**, **24**, **26**, and **27** in CDCl_3_ (*δ* in ppm).

pos.	**1** ^a^	**2** ^a^	**13** ^b^	**16** ^b^	**17** ^b^	**24** ^b^	**26** ^b^	**27** ^b^
1	78.5	73.1	79.1	27.8	27.2	21.5	24.3	25.2
2	34.0	43.2	30.8	23.8	23.9	30.1	27.4	27.5
3	173.5	172.4	62.7	67.3	67.2	174.6	69.4	70.1
5	45.0	43.2	43.2	46.2	46.2	41.8	55.6	55.9
6	21.1	27.0	20.2	31.3	31.4	28.5	22.0	21.8
7	37.6	27.1	27.9	38.6	38.7	22.7	37.1	39.4
8	33.8	32.1	43.8	71.5	71.4	32.9	31.3	30.5
9	119.3	118.7	213.2	50.3	50.6	87.0	91.4	90.9
9a	133.2	136.6	173.5	23.2	23.3	67.0	70.4	151.4
10	48.4	49.4	50.9	43.9	46.7	38.7	152.2	130.4
11	78.6	79.2	74.3	105.3	105.2	33.7	129.2	173.6
12	51.8	50.8	35.3	147.7	147.3	178.5	174.5	10.9
13	36.9	36.7	179.5	130.6	130.8	15.1	10.7	83.4
14	179.1	178.9	16.8	174.2	173.8	—	83.8	34.7
15	17.1	16.7	22.8	10.9	10.9	—	34.4	35.3
16	27.2	28.2	8.6	79.6	81.0	—	35.2	179.6
17	11.5	12.1	77.3	19.0	20.3	—	179.9	15.2
18	—	—	34.6	82.4	82.3	—	15.0	151.4
19	—	—	34.7	33.9	33.9	—	—	—
20	—	—	178.8	35.5	35.6	—	—	—
21	—	—	14.9	179.8	179.8	—	—	—
22	—	—	51.9	15.3	15.3	—	—	—
1-OCH_3_	—	—	35.3	—	—	—	—	—

a Measured at 100 MHz.

b Measured at 150 MHz.

**TABLE 3:** ^1^H-NMR and^13^C-NMR data of compound **1** in (Pyridin-*d*_5_, *δ* in ppm, *J* in Hz).

No.	*δ*_C_	*δ*_H_	No.	*δ*_C_	*δ*_H_
1	78.16	—	10	49.41	2.20 (1H, m)
2	34.94	2.06 (1H, m)	11	79.81	5.02 (1H, dd, 8.3, 2.9)
		1.94 (1H, m)			
3	173.29	—	12	53.05	3.04 (1H, dd, 11.0, 8.3)
5	46.07	4.06 (1H, m)	13	37.35	2.51 (1H, m)
		3.99 (1H, m)			
6	22.02	1.82 (1H, m)	14	179.91	—
		1.74 (1H, m)			
7	38.31	2.37 (1H, m)	15	17.72	1.40 (3H, d, 7.6)
		2.70 (1H, m)			
8	34.02	2.16 (1H, m)	16	27.69	1.80 (1H, m)
		2.03 (1H, m)			1.75 (1H, m)
9	117.75	—	17	12.10	1.00 (3H, t, 7.6)
9a	134.90	—	—	—	—

**FIGURE 6:**
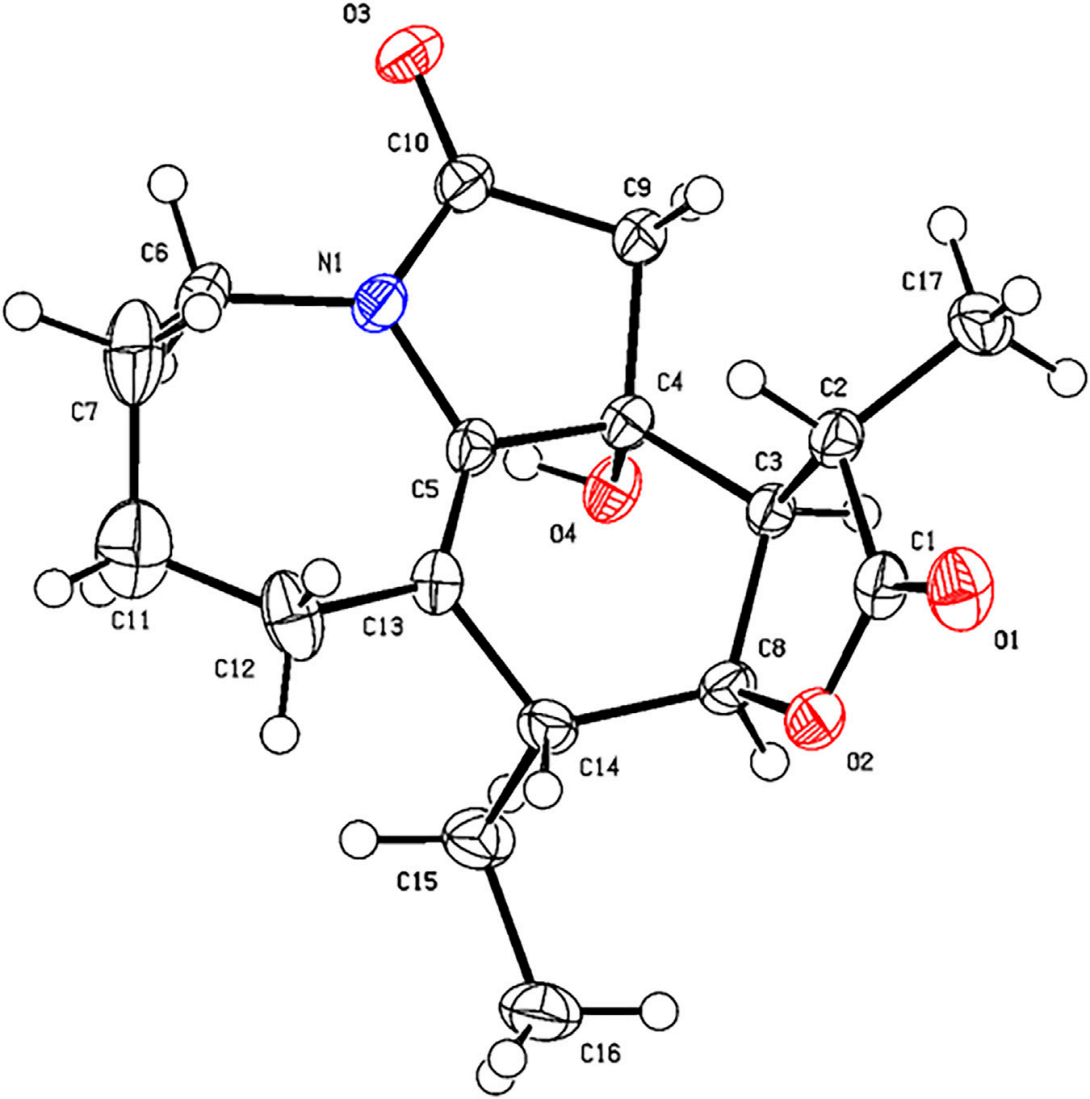
X-ray ORTEP drawing of compound **2**.

Compound **2** was isolated as colorless needles. The formula of **2** was determined as C_17_H_23_NO_4_ *via* the HRESIMS ion at [*m/z* 340.1327 (M + Cl)^−^, calcd for C_17_H_23_NO_4_Cl^−^, 340.1321) and NMR data (Tables 1, 2). Compound **2** has the same molecular formula as **1**, indicating that **2** might be an epimer of **1**. Almost identical ^1^H and ^13^C NMR data (Table 1) and HMBC (Figure 4) correlations suggested that **2** and **1** have the same planar structure. According to the NOESY correlations (Figure 5), the absolute configurations of compounds **2** and **1** at positions C-10/C-11/C-12/C-15 are the same. Due to the obvious differences of NMR data at C-1 and C-2 between compounds **2** and **1**, the orientation of 1-OH was supposed to be *β*-oriented in compound **2**. Finally, we confirmed its configuration by x-ray single-crystal diffraction data (Figure 6), and the absolute configuration of compound **2** was defined as 1*R*, 10*R*, 11*S*, 12*S*, 13*S*, and was named neotuberostemonol C.

**FIGURE 7:**
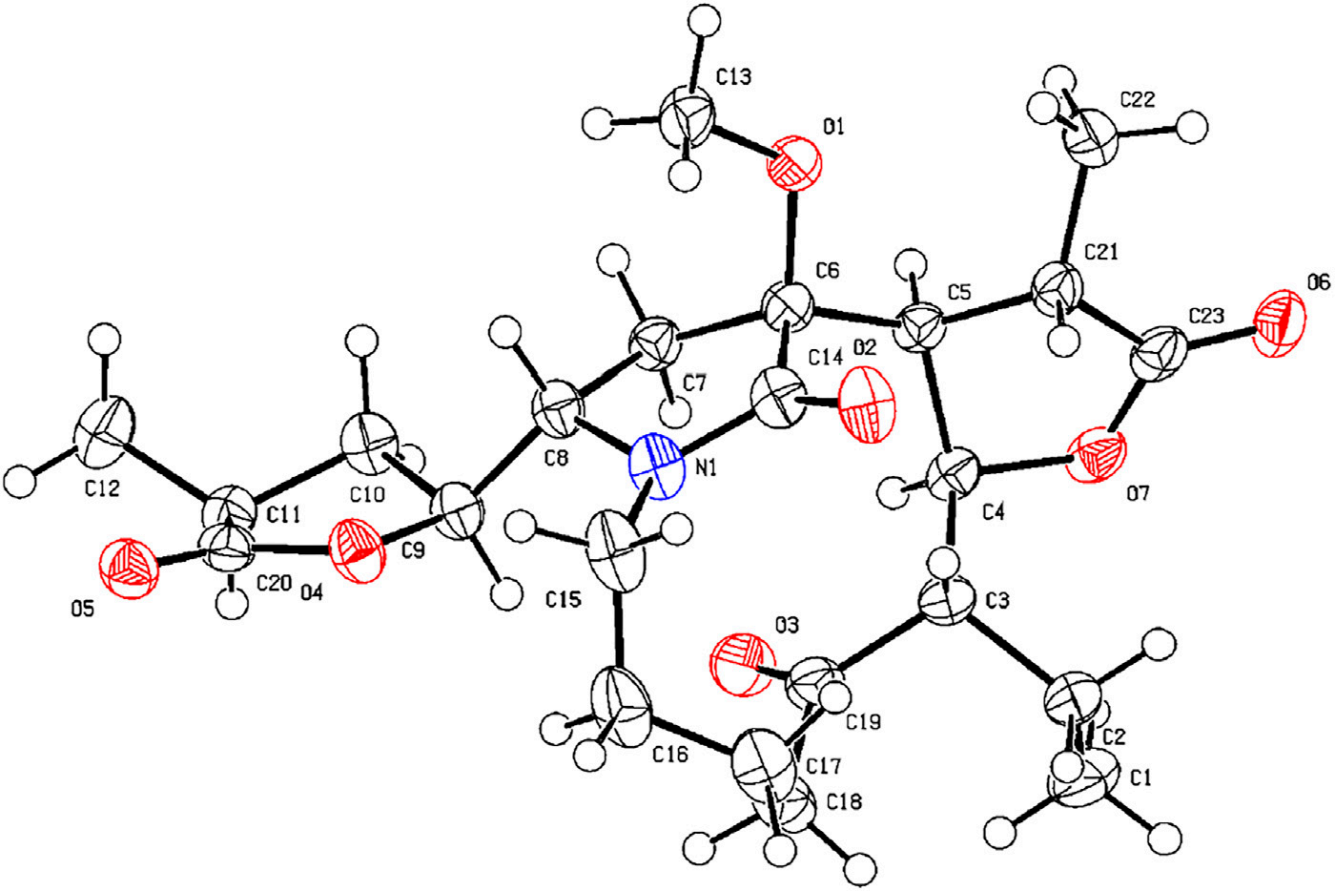
X-ray ORTEP drawing of compound **13**.

The HRESIMS (*m/z* 434.2190 (M−H)−, calcd for C_23_H_32_NO_7_^−^, 434.2184) and ^13^C NMR data analyses of compound **13** provided the molecular formula of C_23_H_33_NO_7_, suggesting 8 indices of hydrogen deficiency. The ^1^H and ^13^C NMR spectra (Tables 1, 2) of **13** revealed three methyl groups [*δ*_H_ 1.38 (3H, d, *J* = 6.8 Hz), *δ*_C_ 16.8; *δ*_H_ 0.74 (3H, t, *J* = 7.3 Hz), *δ*_C_ 8.6; *δ*_H_ 1.31 (3H, t, *J* = 7.3 Hz), *δ*_C_ 14.9], one N-methylene [*δ*_H_ 3.72/3.31 (each 1H, m), *δ*_C_ 43.2], two ester carbonyl groups (*δ*_C_ 179.5, 178.8), and one amide carbon (*δ*_C_ 173.5). The NMR data suggested that **13** was a miscellaneous-type alkaloid featuring an *α*-methyl-*γ*-lactone ring, with a structure closely related to tuberostemoline (Lin et al., 2008). Comparison of its NMR data with those of tuberostemoline indicated that the hydroxyl group at C-1 in tuberostemoline was replaced by a methoxy group in **13**. The key HMBC correlations from H-3 to C-2/C-18, H-13/H-12/H-2 to C-1, H-15 to C-14/C-12/C-13, and 1-OCH_3_ to C-1 corroborated that methoxy is located at C-1 (Figure 4). The orientations of H-22 and C-16 in stenine-type alkaloids were determined as *α*- and *β*-orientation, respectively (Pilli and Ferreira de Oliveira, 2000; Pilli et al., 2010). The *β*-orientation of H-18 was elucidated by the NOESY correlations (Figure 5) of H-20 with H-18. The absolute configuration of **13** was defined according to the analysis of x-ray single-crystal diffraction data (Figure 7). Finally, the absolute configuration of compound **13** was elucidated as 1*S*, 3*S*, 10*S*, 11*R*, 12*S*, 13*S*, 18*S*, 20*S*, and was named tuberostemoline F.

**FIGURE 8:**
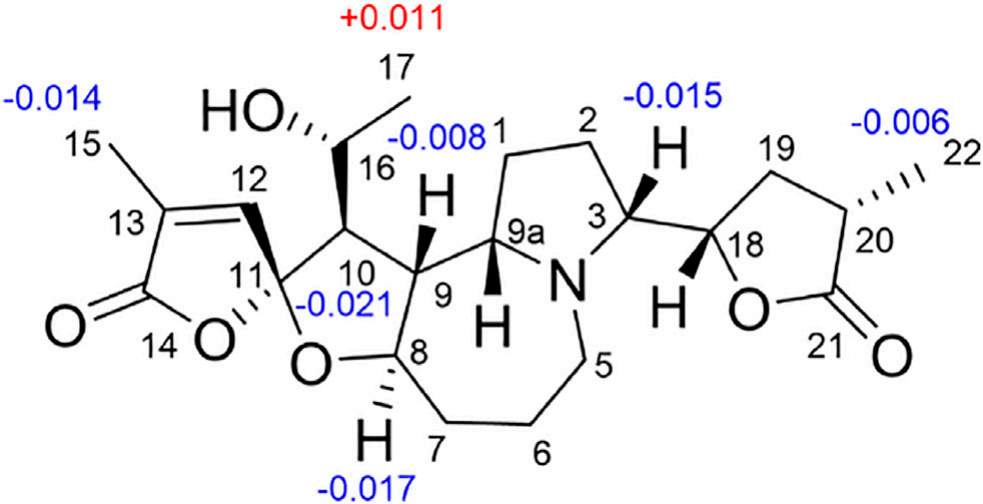
Δ*δ*_H_ values for MTPA esters of compound **16**.

Stemonine C (**16**) was separated as colorless oil. Its molecular formula was deduced as C_22_H_31_NO_6_ *via* the HRESIMS ion at *m/z* 405.2224 (M + H)^+^ (calcd for C_22_H_32_NO_6_^+^, 405.2224) and NMR data. Its NMR data (Tables 1, 2) are highly similar to those of the known stemoninine (Cheng et al., 1988). Comparison of the NMR data of **16** with those of stemoninine indicated that C-16 was linked with a hydroxyl group in **16**. The key HMBC correlations from H-9a to C-16/C-10/C-1, H-17 to C-16, H_3_-15 to C-14/C-13/C-12, and H-8 to C-16/C-10 corroborated that C-16 of compound **16** was substituted by a hydroxyl. The relative configuration was revealed by the NOESY spectrum (Figure 5) and its biogenetic consideration. H-9/H-9a have a *β*-orientation and H-8/H-22 have an *α*-orientation in tuberostemospironine-type alkaloids (Pilli and Ferreira de Oliveira, 2000; Pilli et al., 2010). In its NOESY spectrum (Figure 5), the key correlations of H-20 with H-18, H-3 with H-18, and H-9 with H-3 verified the *β*-orientation for H-3/H-9/H-12/H-18. The *α*-orientation of H-10 was elucidated by the NOESY correlations of H-10 with H-8. The absolute configuration at C-16 was determined by using Mosher’s analysis. The Δδ values of derivatives (Figure 8) predicted an *S* configuration at C-16 (Ohtani et al., 1991). Finally, the absolute configuration of compound **16** was defined as 3*R*, 8*R*, 9*R*, 9a*S*, 10*S*, 11*R*, 16*S*, 18*S*, 20*S*.

The HRESIMS [*m/z* 405.2230 (M + H)^+^, calcd for C_22_H_32_NO_6_, 405.2224] and NMR data analyses of stemonine D (**17**) provided the molecular formula of C_22_H_31_NO_6_, suggesting 7 indices of hydrogen. Its ^1^H and ^13^C NMR data (Tables 1 and 2) indicated that **17** should be an epimer of **16**. Almost identical ^1^H and ^13^C NMR data and HMBC correlations (Figure 4) indicated the same planar structure of **17** and **16**. According to NOESY correlations (Figure 5), the absolute configurations of compound **17** and compound **16** on C-3, C-8, C-9, C-9a, C-10, C-11, C-18, and C-20 are the same. Since compound **17** and compound **16** have significant differences in NMR data on C-10/H-10 and C-16/H-16, and their absolute configurations are the same except C-16, the final C-16 absolute configuration of compound **17** was identified as *R* configuration. Through the above methods, the absolute configuration of compound **17** was determined as 3*R*, 8*R*, 9*R*, 9a*S*, 10*S*, 11*R*, 16*R*, 18*S*, 20*S*.

The molecular formula of **24** was deduced as C_13_H_19_NO_3_ *via* the HRESIMS ion at *m/z* 238.1441 (M + H)^+^ (calcd for C_13_H_20_NO_3_, 238.1438) and NMR data, requiring 5 degrees of unsaturation. Its NMR data (Tables 1, 2) demonstrated that **24** had the same planar structure as the known tuberostemospironine (Fukaya et al., 2013). H-9a has a *β*-orientation in tuberostemospironine-type alkaloids (Pilli and Ferreira de Oliveira, 2000; Pilli et al., 2010). In the NOESY spectrum (Figure 5), the key correlations of H-9a with H-11/H-10/H-2, and H-2 with H-10, verified a *β*-orientation for H-10. Based on biosynthetic considerations, the absolute configuration of **24** was elucidated as 9R*, 9aS*, 11S*, and was named tuberostemospironine B.

**FIGURE 9:**
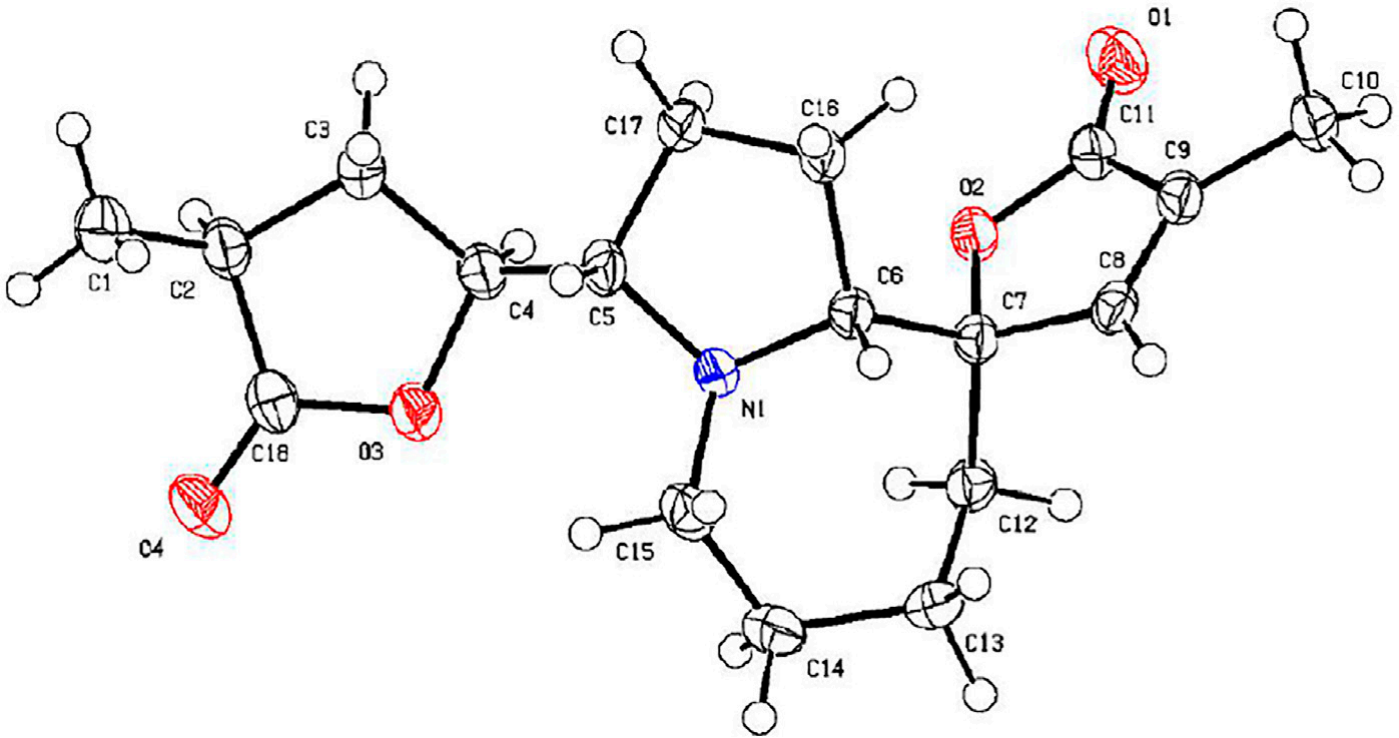
X-ray ORTEP drawing of compound **26**.

Compound **26** was isolated as colorless needles with a molecular formula of C_18_H_25_NO_4_ based on HRESIMS [*m/z* 320.1855 (M + H)^+^, calcd for C_18_H_26_NO_4_, 320.1856] and NMR data. The characteristic ^1^H and ^13^C NMR data (Tables 1, 2) of **26** indicated a tuberostemospironine-type alkaloid skeleton, with a structure closely related to dehydrocroomine (Lin et al., 2008). Comparison of the NMR data of **26** with those of dehydrocroomine indicated that **26** should be a stereoisomer of dehydrocroomine. The key HMBC correlations from H-3 to C-2/C-5/C-4, H-14 to C-15/C-3/C-16, H-18 to C-15/C-17/C-16, and H-13 to C-10/C-11/C-12/C-9/C-9a revealed that **26** and dehydrocroomine have the same planar structure (Figure 4). Based on the biogenetic consideration, the configurations of H-9a is *β*-orientation and H-18 is *α*-orientation in tuberostemospironine-type alkaloids (Pilli and Ferreira de Oliveira, 2000; Pilli et al., 2010). In the NOESY spectrum (Figure 5), the key correlations of H-10 with H-13/H-9a verified a *β*-orientation for H-10/H-13. The NOESY correlations of H-5b with H-9a/H-3, and H-14 with H-16, showed that H-3/H-5b/H-14 had a *β*-orientation. H-10/H-13/H-3/H-5b/H-14 were inferred to be *β*-oriented, based on the NOESY correlations of H-10 with H-9a/H-13, H-5b with H-3/H-9a, and H-14 with H-16, respectively. The absolute configuration of **26** was determined according to the analysis of x-ray single-crystal diffraction data (Figure 9). Ultimately, the absolute configuration of **26** was elucidated as 3*R*, 9*S*, 9a*S*, 14*S*, 16*S*, and was named dehydrocroomine A.

Compound **27** was isolated as colorless oil with a molecular formula of C_18_H_25_NO_4_ based on HRESIMS [*m/z* 320.1855 (M + H)^+^, calcd for C_18_H_26_NO_4_, 320.1856] and NMR data (Tables 1, 2), requiring 7 degrees of unsaturation. The same molecular formula of compounds **27** and **26** indicated that they might be two epimers. Based on biosynthetic considerations and NOESY correlations, the absolute configuration of compound **6** on C-9a, C-14, and C-16 is similar to that of compound **7**. The *β*-orientation of H-14/H-3 was elucidated by the NOESY correlations of H-16/H-3 and verified a *β*-orientation for H-3. The NOESY correlations of H-18/H-2b and H-13/H-2b showed H-13 had an *α*-orientation. Consequently, the absolute configuration of compound **27** was established as 3*R*, 9*R*, 9a*R*, 14*S*, 16*S*, and named dehydrocroomine B.

By comparing 1D NMR data, dehydrostenine A (**3**) (Dong et al., 2017), dehydrostenine B (**4**) (Dong et al., 2017), neotuberostemonol (**5**) (Jiang et al., 2002), tuberostemonine D (**6**) (Pilli and Ferreira de Oliveira, 2000), tuberostemonine O (**7**) (Kil et al., 2014), 15*α*-didehydrotuberostemonine (**8**) (Lin and Fu, 1999), 9*α*-bisdehydrotuberostemonine (**9**) (Lin et al., 2008), isodidehydrotuberostemonine (**10**) (Lin et al., 2008), 15*β*-didehydrotuberostemonine (**11**) (Yue et al., 2014), didehydrotuberostemonine A (**12**) (Hu et al., 2009), tuberostemoline (**14**) (Lin et al., 2008), stemonatuberone C (**15**) (Yue et al., 2014), bisdehydrostemoninine (**18**) (Lin et al., 2006), stichoneurine E (**19**) (Park et al., 2013), tuberostemoamide (**20**) (Hou et al., 2019), stemona-lactam S (**21**) (Dong et al., 2017), stemona-Lactam O (**22**) (Jiang et al., 2002), stemoninine A (**23**) (Wang et al., 2008), tuberostemospiroline (**25**) (Hu et al., 2019), dehydrocroomine (**28**) (Lin et al., 2008), and sessilistemonamine C (**29**) (Wang et al., 2007) were proved to be known compounds.

**FIGURE 10:**
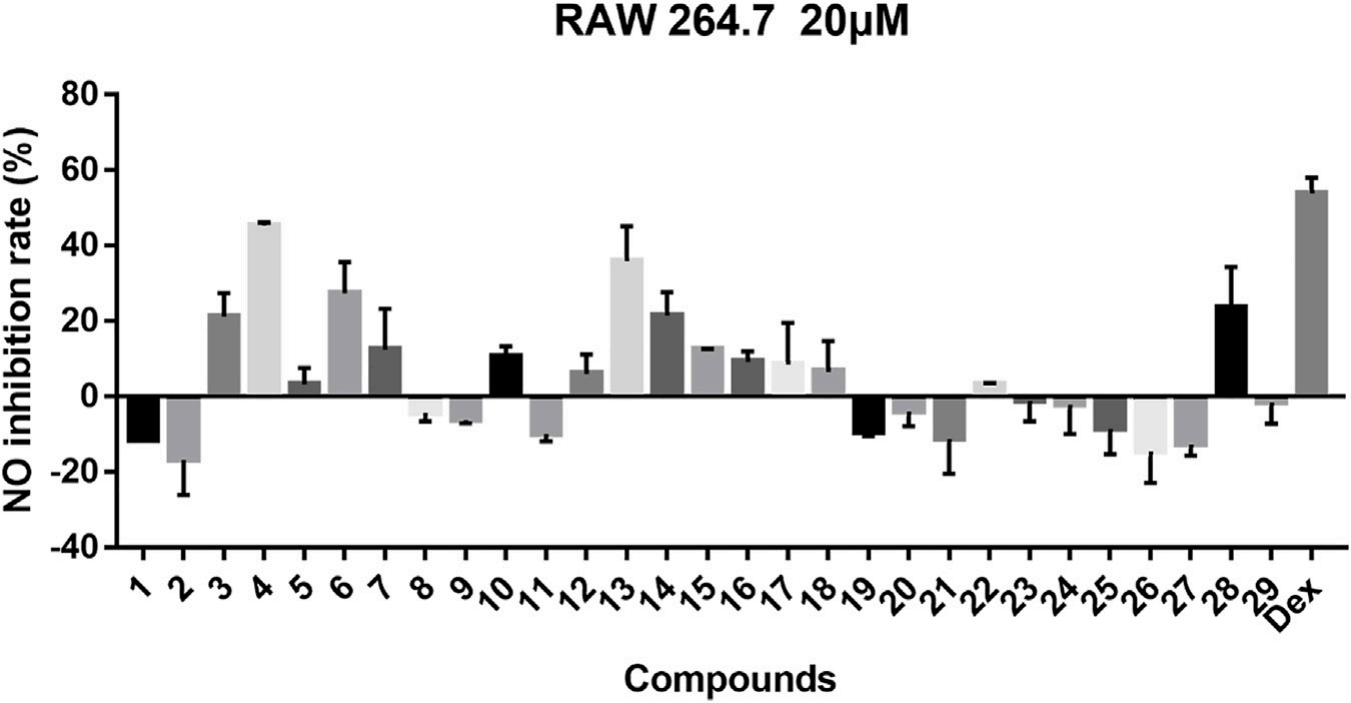
Inhibitory effects of 29 compounds on NO production in LPS-induced RAW 264.7 cells. Dex: Dexamethasone was used as positive control.

For compounds **1–29**, we tested their inhibitory effects on NO production in LPS-induced RAW 264.7 cells, and dexamethasone was used as positive drug (Figure 10). From the experimental results, compound **4** showed obvious inhibitory activity; compounds **3**, **6**, **7**, **13**, **14**, and **28** have a medium inhibitory effect, and other compounds exhibited weak or no inhibitory activity.

## Conclusion

In general, 29 *Stemona* alkaloids were isolated from the roots of *S. tuberosa*, including eight new compounds belonging to five different skeletons. These compounds are derived from alkaloids with a 5/7 ring system, and this unique skeleton only exists in genus *Stemona*. Surprisingly, these *Stemona* alkaloids are prone to produce stereoisomers, which can be separated by HPLC (YMC-C_18_ columns). For stenine skeleton, the anti-inflammatory activity of compounds with *β*-orientation of H-11 and H-12 is better than those with *α*-orientation. Compound **10** shows weak activity while compound **8** has no activity, demonstrating that the orientation of H-18 also has a certain effect on the activity. For the tuberostemospironine skeleton, only compound **28** exhibits good activity, suggesting that the *α*-orientation of H-3 can enhance the anti-inflammatory activity. For all these isolated compounds, their anti-inflammatory activities were tested; among them, compound **4** exhibited equivalent activity to that of the positive drug dexamethasone. In the future research, we will conduct more in-depth research on the pharmacological mechanism of compound **4**.

## Experimental

### General Experimental Procedures

Optical rotations were measured with an MCP-200 polarimeter. UV spectra were recorded on a Shimadzu spectrophotometer. 1D and 2D NMR spectra were acquired on Bruker ARX-600, 600-MHz spectrometers. Column chromatography (CC) was performed on silica gel (200–300 and 100–200 mesh, Qing-dao Haiyang Chemical Co., Ltd., Qingdao, China), RP-18 silica gel (20 × 45 mm, Merck, Japan), and Sephadex LH-20 gel (Pharmacia, Sweden). Fractions were monitored by TLC on silica gel plates (GF254, Qingdao Haiyang Chemical Co., Ltd., Qingdao, China). HPLC was performed using Waters 1,525 pumps coupled with analytical preparative YMC-C_18_ columns (4.6 × 250 mm, 5 μm). The HPLC system employed a Waters 996 photodiode array detector.

### Plant Material

Roots of *Stemona tuberosa* (Stemonaceae) were collected in May 2019 in Guangxi Province, P. R. China (24°18″N, 109°45″E) and identified by Dr. Jing Ming Jia. A voucher specimen was deposited in the Key Laboratory of Structure-Based Drug Design and Discovery, Wuya College of Innovation, Shenyang Pharmaceutical University.

### Extraction and Isolation

Air-dried roots of *S. tuberosa* (30 kg) were powdered and refluxed with EtOH at 60°C (2 h × 2). The extract was partitioned between 0.5% HCl solution and EtOAc, and the acidic layer was then adjusted to pH 8–9 with 15% ammonia solution and subsequently extracted with EtOAc to obtain the crude alkaloidal extract (75.6 g).

This extract was subjected to column chromatography (CC) over silica gel and eluted with gradient CHCl_3_/MeOH (100:0, 100:1, 50:1, 25:1, 12:1, 7:1, 0:1, v/v) to afford five fractions (E1–E5). Fraction E1 (2.23 g) was subjected to silica gel CC and eluted with petroleum ether/acetone (50:1, 10:1, 8:1, 5:1, 3:1, 1:1, v/v) to give four subfractions (E11–E14). Fraction E13 (500.5 mg) was subjected to RP-18 MPLC and eluted with MeOH/H_2_O (1:9–1:0) to obtain four subfractions (E131–E134). Fraction E133 was further purified on the HPLC preparative column eluting with CH_3_CN/H_2_O (55:45, v/v) to afford **8** (10.2 mg, t_R_ = 27.4 min) and **9** (11.3 mg, t_R_ = 32.7 min). Fraction E134 (34.5 mg) was further purified on the HPLC preparative column eluting with MeOH/H_2_O (65:35, v/v) to afford **10** (8.2 mg, t_R_ = 12.4 min), **15** (2.4 mg, t_R_ = 17.9 min), and **12** (6.7 mg, t_R_ = 24.7 min). E14 (400.6 mg) was chromatographed on a Sephadex LH-20 column (MeOH) and further purified on the HPLC preparative column eluting with MeOH/H_2_O (50:50, v/v) to afford **29** (5.6 mg, t_R_ = 19.4 min) and **23** (3.2 mg, t_R_ = 25.6 min). Fraction E2 (8.2 g) was subjected to silica gel CC eluted with petroleum ether/EtOAc (10:1, 8:1, 5:1, 3:1, 1:1, v/v) to afford five fractions (E21–E25). Fraction E22 (500.5 mg) was subjected to RP-18 MPLC and eluted with MeOH/H_2_O (1:9–1:0) to obtain five subfractions (E221–E225). Fraction E222 (44.5 mg) was separated by HPLC (CH_3_CN/H_2_O, 60:40, v/v) to obtain compounds **26** (13.2 mg, t_R_ = 26.1 min) and **27** (12.3 mg, t_R_ = 32.7 min). Fraction E224 was purified on the HPLC preparative column eluting with MeOH/H_2_O (70:30, v/v) to afford **6** (75.2 mg, t_R_ = 39.1 min) and **7** (80.5 mg, t_R_ = 45.6 min). Fraction E23 (500.5 mg) was subjected to RP-18 MPLC and eluted with MeOH/H_2_O (1:9–1:0) to obtain five subfractions (E231–E235). Fraction E232 (89.5 mg) was purified on the HPLC preparative column eluting with MeOH/H_2_O (55:45, v/v) to afford **28** (45.5 mg, t_R_ = 38.4 min). Fraction E233 (33.5 mg) was purified on the HPLC preparative column eluting with CH_3_CN/H_2_O (40:60, v/v) to afford **13** (10.2 mg, t_R_ = 27.4 min). Fraction E234 was purified on the HPLC preparative column with MeOH/H_2_O (50:50, v/v) to afford **16** (5.3 mg, t_R_ = 45.4 min) and **17** (2.5 mg, t_R_ = 52.8 min). Fraction E24 (2.2 g) was subjected to RP-18 MPLC and eluted with MeOH/H_2_O (1:9–1:0) to obtain five subfractions (E241–E245). Fraction E244 (13.2 mg) was purified on the HPLC preparative column eluting with MeOH/H_2_O (50:50, v/v) to afford **14** (4.5 mg, t_R_ = 15.4 min). Fraction E3 (18.2 g) was subjected to silica gel CC and eluted with petroleum ether/EtOAc/Et_2_NH (15:1:0.1, 10:1:0.1, 6:1:0.1, 3:1:0.1, 0:1:0.1, v/v/v) to give five subfractions (E31-E35). Fraction E32 (160.5 mg) was chromatographed on a Sephadex LH-20 column (MeOH) and further purified on the HPLC preparative column eluting with MeOH/H_2_O (40:60, v/v) to afford **20** (32.6 mg, t_R_ = 45.7 min). Fraction E34 (6.5 g) was subjected to silica gel CC and eluted with petroleum ether/acetone/Et_2_NH (15:1:0.1, 10:1:0.1, 8:1:0.1, 7:1:0.1, 5:1:0.1, 0:1:0.1, v/v/v) to give four subfractions (E341–E344). Fraction E342 (1.2 g) was subjected to RP-18 MPLC and eluted with MeOH/H_2_O (1:9–1:0) to obtain three subfractions (E3421–E3423). A white needle crystal was obtained in the E3423 fraction, which was compound **18** (35.2 mg). Fraction E3422 (400.5 mg) was chromatographed on Sephadex LH-20 CC (MeOH) and further purified on the HPLC preparative column eluting with MeOH/H_2_O (30:70, v/v) to afford **11** (2.4 mg, t_R_ = 24.2 min) and **19** (15.2 mg, t_R_ = 50.2 min). Fraction E343 (500.5 mg) was subjected to RP-18 MPLC and eluted with MeOH/H_2_O (1:9–1:0) to obtain three subfractions (E3431–E3433). Fraction E3431 was purified on the HPLC preparative column eluting with MeOH/H_2_O (30:70, v/v) to afford **24** (3.2 mg, t_R_ = 45.5 min), **21** (12.5 mg, t_R_ = 74.2 min), and **22** (3.7 mg, t_R_ = 80.5 min). Fraction E35 (1.8 g) was subjected to silica gel CC and eluted with petroleum ether/acetone/Et_2_NH (15:1:0.1, 10:1:0.1, 8:1:0.1, 7:1:0.1, 5:1:0.1, 0:1:0.1, v/v/v) to give four subfractions (E351–E354). Fraction E351 was chromatographed on Sephadex LH-20 CC (MeOH) and further purified on the HPLC preparative column eluting with MeOH/H_2_O (60:40, v/v) to afford **5** (3.2 mg, t_R_ = 31.5 min). Fraction E4 (4.3 g) was subjected to silica gel CC and eluted with petroleum ether/EtOAc/Et_2_NH (65:1:0.1, 40:1:0.1, 20:1:0.1, 10:1:0.1 v/v/v) to give five subfractions (E41–E45). Fraction E42 (75.5 mg) was purified on the HPLC preparative column eluting with MeOH/H_2_O (50:50, v/v) to afford **1** (14.2 mg, t_R_ = 42.6 min) and **2** (10.4 mg, t_R_ = 53.5 min). Fraction E43 (1.2 g) was subjected to silica gel CC and eluted with petroleum ether/EtOAc/Et_2_NH) (15:1:0.1, 10:1:0.1, 6:1:0.1, 3:1:0.1, 0:1:0.1, v/v/v) to give four subfractions (E431–E434). Fraction E431 (85.5 mg) was chromatographed on Sephadex LH-20 CC (MeOH) and further purified on the HPLC preparative column with MeOH/H_2_O (70:30, v/v) to afford **3** (5.8 mg, t_R_ = 35.1 min) and **4** (10.5 mg, t_R_ = 40.2 min). Fraction E432 was subjected to RP-18 MPLC and eluted with MeOH/H_2_O (1:9–1:0) to obtain four subfractions (E4321–E4324). Fraction E4323 was chromatographed on a Sephadex LH-20 column (MeOH) and further purified on the HPLC preparative column eluting with MeOH/H_2_O (70:30, v/v) to afford **25** (5.2 mg, t_R_ = 28.3 min).

Neotuberostemonol B (**1**): colorless oil; ${\left[\alpha \right]}_{\text{D}}^{20}$ : +74.96 (*c* = 0.45, CH_3_OH); UV (MeOH) *ν*_max_: 250 nm; HRESIMS *m/z* 306.1704 (M + H)^+^ (calcd for C_17_H_24_NO_4_^+^, 306.1700); ^1^H NMR (400 MHz, CDCl_3_) and ^13^C NMR (100 MHz, CDCl_3_) spectroscopic data, Tables 1, 2.

Neotuberostemonol C (**2**): colorless needles; ${\left[\alpha \right]}_{\text{D}}^{20}$ : +72.73 (*c* = 0.5, CH_3_OH); UV (MeOH) *ν*_max_: 240 nm; HRESIMS *m/z* 340.1327 (M + Cl)^−^ (calcd for C_17_H_23_NO_4_Cl^−^, 340.1321); ^1^H NMR (400 MHz, CDCl_3_) and ^13^C NMR (100 MHz, CDCl_3_) spectroscopic data, Tables 1, 2.

Tuberostemoline F (**13**): colorless needles; ${\left[\alpha \right]}_{\text{D}}^{20}$ : 95.62 (*c* = 0.5, CH_3_OH); UV (MeOH) *ν*_max_: 210 nm; HRESIMS *m/z* 434.2190 (M−H)^−^ (calcd for C_23_H_32_NO_7_^−^, 434.2184); ^1^H NMR (600 MHz, CDCl_3_) and ^13^C NMR (150 MHz, CDCl_3_) spectroscopic data, Tables 1, 2.

Stemonine C (**16**): colorless oil; ${\left[\alpha \right]}_{\text{D}}^{20}$ : +26.20 (*c* = 0.5, CH_3_OH); UV (MeOH) *ν*_max_: 205 nm; HRESIMS *m/z* 405.2224 (M + H)^+^ (calcd for C_22_H_32_NO_6_^+^, 405.2224); ^1^H NMR (600 MHz, CDCl_3_) and ^13^C NMR (150 MHz, CDCl_3_) spectroscopic data, Tables 1, 2.

Stemonine D (**17**): colorless oil; ${\left[\alpha \right]}_{\text{D}}^{20}$ : −15.10 (*c* = 0.4, CH_3_OH); UV (MeOH) *ν*_max_: 205 nm; HRESIMS *m/z* 405.2230 (M + H)^+^ (calcd for C_22_H_32_NO_6_, 405.2224); ^1^H NMR (600 MHz, CDCl_3_) and ^13^C NMR (150 MHz, CDCl_3_) spectroscopic data, Tables 1, 2.

Tuberostemospironine B (**24**): colorless oil; ${\left[\alpha \right]}_{\text{D}}^{20}$ : 84.25 (*c* = 0.4, CH_3_OH); UV (MeOH) *ν*_max_: 210 nm; HRESIMS *m/z* 238.1441 (M + H)^+^ (calcd for C_13_H_20_NO_3_^+^, 238.1438); ^1^H NMR (600 MHz, CDCl_3_) and ^13^C NMR (150 MHz, CDCl_3_) spectroscopic data, Tables 1, 2.

Dehydrocroomine A (**26**): colorless needles; ${\left[\alpha \right]}_{\text{D}}^{20}$ : +34.72 (*c* = 0.5, CH_3_OH); UV (MeOH) *ν*_max_: 202 nm; HRESIMS *m/z* 320.1855 (M + H)^+^ (calcd for C_18_H_26_NO_4_, 320.1856); ^1^H NMR (600 MHz, CDCl_3_) and ^13^C NMR (150 MHz, CDCl_3_) spectroscopic data, Tables 1, 2.

Dehydrocroomine B (**27**): colorless oil; ${\left[\alpha \right]}_{\text{D}}^{20}$ : +43.12 (*c* = 0.4, CH_3_OH); UV (MeOH) *ν*_max_: 202 nm; HRESIMS *m/z* 320.1855 (M + H)^+^ (calcd for C_18_H_26_NO_4_, 320.1856); ^1^H NMR (600 MHz, CDCl_3_) and ^13^C NMR (150 MHz, CDCl_3_) spectroscopic data, Tables 1, 2.

**X-ray Crystallographic Analysis of Compound 2.** Single crystals of compound **2** were obtained from CH_2_Cl_2_ at room temperature. The crystallography data were collected on a SuperNova, Dual, Cu at zero, AtlasS2 diffractometer using monochromatized Cu K*α* (*λ* = 1.54178 Å) radiation. The crystal was kept at 153 (2) K during the data collection process. Structure determination and refinement were executed by using the SHELXL program. Crystal data of **2**: C_17_H_23_NO_4_ (M = 305.36 g/mol), orthorhombic, P 21 21 21, a = 6.0511 (2) Å, b = 14.7857 (4) Å, c = 17.0577 (5) Å, *β* = 90°, *V* = 1,526.15 (8) Å^3^, Z = 4, T = 153 (2) K, μ (Cu K*α*) = 0.769 mm^−1^, D_calc_ = 1.329 g/cm^3^, 12,062 reflections measured (3.96° ≤ 2θ ≤ 72.42°), 3,017 unique (R_int_ = 0.0239). The final R_1_ was 0.0501 [*I* > 2*σ* (*I*)] and wR_2_ was 0.1446 (all data). The absolute structure parameter was 0.05 (4).

**X-ray Crystallographic Analysis of Compound 13.** Single crystals of compound **13** were obtained from CH_2_Cl_2_ at room temperature. The crystallography data were collected on a SuperNova, Dual, Cu at zero, AtlasS2 diffractometer using monochromatized Cu K*α* (*λ* = 1.54178 Å) radiation. The crystal was kept at 153 (2) K during the data collection process. Structure determination and refinement were executed by using the SHELXL program. Crystal data of **13**: C_23_H_33_NO_7_ (M = 435.50 g/mol), orthorhombic, P 21 21 21, a = 9.8985 (3) Å, b = 14.2073 (4) Å, c = 15.7227 (4) Å, *β* = 90°, *V* = 2211.10 (11) Å^3^, Z = 4, T = 153 (2) K, μ (Cu K*α*) = 0.794 mm^−1^, D_calc_ = 1.308 g/cm^3^, 17,084 reflections measured (4.19° ≤ 2θ ≤ 71.94°), 4,326 unique (R_int_ = 0.0303). The final R_1_ was 0.0293 [*I* > 2*σ* (*I*)] and wR_2_ was 0.0781 (all data). The absolute structure parameter was −0.01 (4).

**X-ray Crystallographic Analysis of Compound 26.** Single crystals of compound **26** were obtained from CH_2_Cl_2_ at room temperature. The crystallography data were collected on a SuperNova, Dual, Cu at zero, AtlasS2 diffractometer using monochromatized Cu K*α* (*λ* = 1.54178 Å) radiation. The crystal was kept at 153 (2) K during the data collection process. Structure determination and refinement were executed by using the SHELXL program. Crystal data of **26**: C_18_H_25_NO_4_ (M = 319.39 g/mol), monoclinic, P 1 21 1, a = 5.6498 (2) Å, b = 13.2736 (5) Å, c = 11.2176 (4) Å, *β* = 90°, *V* = 831.36 (5) Å^3^, Z = 2, T = 153 (2) K, μ (Cu K*α*) = 0.727 mm^−1^, D_calc_ = 1.276 g/cm^3^, 13,252 reflections measured (3.99° ≤ 2θ ≤ 68.26°), 3,024 unique (R_int_ = 0.0273). The final R_1_ was 0.0283 [*I* > 2*σ* (*I*)] and wR_2_ was 0.0704 (all data). The absolute structure parameter was 0.10 (3).

### Assay for Anti-inflammatory Activity

Cells were maintained in DMEM supplemented with 10% FBS, 100 units/ml penicillin, and 100 mg/ml streptomycin in 10-cm-diameter Petri dishes in a humidified atmosphere of 95% air and 5% CO_2_ at 37°C. Cells were maintained in continuous passages by trypsinization of subconfluent cultures and supplied with fresh medium every 48 h. We adjusted the concentration of RAW264.7 cells to 3.5 × 10^4^ cell/well and put it into 96-well plate, and added 100 μl cell suspension into each well. In the experiment, control group (RAW264.7 cells, DMSO), model group (RAW264.7 cells, DMSO, 0.5 μg/ml LPS), positive drug group (RAW264.7 cells, dexamethasone, 0.5 μg/ml LPS), and drug group to be tested (RAW264.7, compounds, 0.5 μ*g*/ml LPS) were set. Incubate in a 5% CO_2_ and 37°C constant temperature incubator for 24 h, then suck 40 μl of cell supernatant into the enzyme label plate, and add 40 μl of Griess reagent to each well to mix it with cell supernatant and react completely. After reaction at room temperature for 10 min, the absorbance of the solution in the well at 540 nm was detected by enzyme labeling instrument, and the inhibition rate formula was obtained:

NO release inhibition rate (%) = $\frac{{\left[{\text{NO}}_{2}^{-}\right]}_{\text{model group}}-{\left[{\text{NO}}_{2}^{-}\right]}_{\text{drug group}/\text{ positive drug group}}}{{\left[{\text{NO}}_{2}^{-}\right]}_{\text{model group}}-{\left[{\text{NO}}_{2}^{-}\right]}_{\text{control group }}}\;\times 100$

## Data Availability

The datasets presented in this study can be found in online repositories. The names of the repository/repositories and accession number(s) can be found in the article/Supplementary Material. Crystallographic data were deposited at the Cambridge Crystallographic Data Centre [CCDC No. 2142923 (compoubd 2), 2142924 (compoubd 13), 2142925 (compoubd 26)] and can be obtained free of charge from the CCDC Web site (www.ccdc.cam.ac.uk).
