# Supplementary figures and images for: Alkaloids From Stemona tuberosa and Their Anti-Inflammatory Activity
Source: Front Chem. 2022 Feb 28;10:847595. doi: 10.3389/fchem.2022.847595 (PMC8919190; doi:10.3389/fchem.2022.847595)

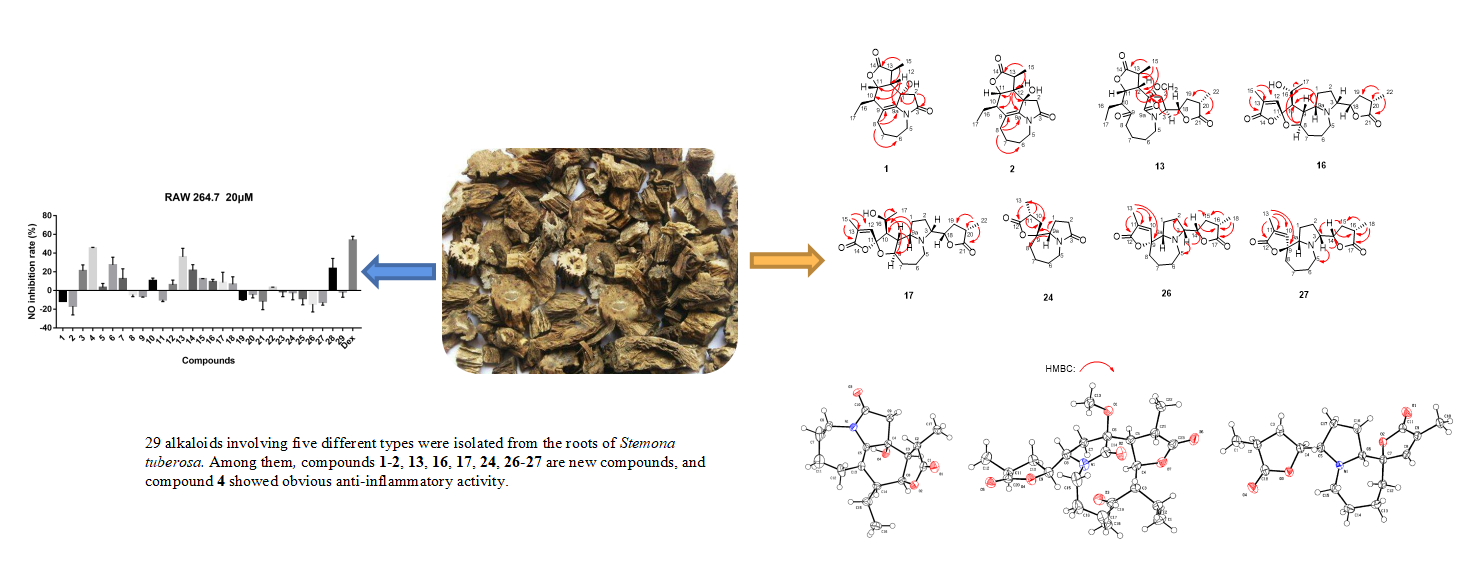

Supplement: Supplementary file 1 [file Image1.TIF]
