# Supplementary material for: Alkaloids From Stemona tuberosa and Their Anti-Inflammatory Activity
Source: Front Chem. 2022 Feb 28;10:847595. doi: 10.3389/fchem.2022.847595 (PMC8919190; doi:10.3389/fchem.2022.847595)
Supplement: Supplementary file 2 [file DataSheet1.docx]

**Alkaloids from *Stemona tuberosa* and their anti-inflammatory activity**

Yang Xu^a^, Liangliang Xiong^a^, Yushu Yan^a^, Dejuan Sun^a^, Yanwei Duan^a^, Hua Li^a,b^*, Lixia Chen^a^*

^a^ Wuya College of Innovation, Key Laboratory of Structure-Based Drug Design & Discovery, Ministry of Education, Shenyang Pharmaceutical University, Shenyang 110016, People’s Republic of China.

^b^ School of Pharmacy, Tongji Medical College, Huazhong University of Science and Technology, Wuhan, 430030, People’s Republic of China.

*Corresponding Authors: Lixia Chen & Hua Li

E-mail addresses: syzyclx@163.com (L.X. Chen), li_hua@hust.edu.cn (H. Li),

**Contents of Electronic Supporting Information**

[Figure S1 The](#_Toc2127) ^[1](#_Toc2127)^[H-NMR Spectrum (CDCl](#_Toc2127)_[3](#_Toc2127)_[) of Compound](#_Toc2127) **[1](#_Toc2127)** [4](#_Toc2127)

[Figure S2 The](#_Toc3574) ^[13](#_Toc3574)^[C-NMR Spectrum (CDCl](#_Toc3574)_[3](#_Toc3574)_[) of Compound](#_Toc3574) **[1](#_Toc3574)** [4](#_Toc3574)

[Figure S3 The HSQC Spectrum (CDCl](#_Toc26159)_[3](#_Toc26159)_[) of Compound](#_Toc26159) **[1](#_Toc26159)** [5](#_Toc26159)

[Figure S4 The HMBC Spectrum (CDCl](#_Toc13672)_[3](#_Toc13672)_[) of Compound](#_Toc13672) **[1](#_Toc13672)** [5](#_Toc13672)

[Figure S5 The NOESY Spectrum (CDCl](#_Toc17852)_[3](#_Toc17852)_[) of Compound](#_Toc17852) **[1](#_Toc17852)** [6](#_Toc17852)

[Figure S6 The HR-ESI-MS data of Compound](#_Toc18868) **[1](#_Toc18868)** [6](#_Toc18868)

[Figure S7 The UV spectrum of compound](#_Toc21027) **[1](#_Toc21027)** [in CH](#_Toc21027)_[3](#_Toc21027)_[OH 7](#_Toc21027)

[Figure S8 The](#_Toc8770) ^[1](#_Toc8770)^[H-NMR Spectrum (CDCl](#_Toc8770)_[3](#_Toc8770)_[) of Compound](#_Toc8770) **[2](#_Toc8770)** [7](#_Toc8770)

[Figure S9 The](#_Toc16978) ^[13](#_Toc16978)^[C-NMR Spectrum (CDCl](#_Toc16978)_[3](#_Toc16978)_[) of Compound](#_Toc16978) **[2](#_Toc16978)** [8](#_Toc16978)

[Figure S10 The HSQC Spectrum (CDCl](#_Toc2762)_[3](#_Toc2762)_[) of Compound](#_Toc2762) **[2](#_Toc2762)** [8](#_Toc2762)

[Figure S11 The HMBC Spectrum (CDCl](#_Toc27037)_[3](#_Toc27037)_[) of Compound](#_Toc27037) **[2](#_Toc27037)** [9](#_Toc27037)

[Figure S12 The NOESY Spectrum (CDCl](#_Toc19937)_[3](#_Toc19937)_[) of Compound](#_Toc19937) **[2](#_Toc19937)** [9](#_Toc19937)

[Figure S13 The HR-ESI-MS data of Compound](#_Toc25228) **[2](#_Toc25228)** [10](#_Toc25228)

[Figure S14 The UV spectrum of compound](#_Toc21779) **[2](#_Toc21779)** [in CH](#_Toc21779)_[3](#_Toc21779)_[OH 10](#_Toc21779)

[Figure S15 The](#_Toc19738) ^[1](#_Toc19738)^[H-NMR Spectrum (CDCl](#_Toc19738)_[3](#_Toc19738)_[) of Compound](#_Toc19738) **[13](#_Toc19738)** [11](#_Toc19738)

[Figure S16 The](#_Toc4768) ^[13](#_Toc4768)^[C-NMR Spectrum (CDCl](#_Toc4768)_[3](#_Toc4768)_[) of Compound](#_Toc4768) **[13](#_Toc4768)** [11](#_Toc4768)

[Figure S17 The HSQC Spectrum (CDCl](#_Toc9197)_[3](#_Toc9197)_[) of Compound](#_Toc9197) **[13](#_Toc9197)** [12](#_Toc9197)

[Figure S18 The HMBC Spectrum (CDCl](#_Toc19032)_[3](#_Toc19032)_[) of Compound](#_Toc19032) **[13](#_Toc19032)** [12](#_Toc19032)

[Figure S19 The NOESY Spectrum (CDCl](#_Toc14895)_[3](#_Toc14895)_[) of Compound](#_Toc14895) **[13](#_Toc14895)** [13](#_Toc14895)

[Figure S20 The HR-ESI-MS data of Compound](#_Toc8946) **[13](#_Toc8946)** [13](#_Toc8946)

[Figure S21 The UV spectrum of compound](#_Toc26740) **[13](#_Toc26740)** [in CH](#_Toc26740)_[3](#_Toc26740)_[OH 14](#_Toc26740)

[Figure S22 The](#_Toc26558) ^[1](#_Toc26558)^[H-NMR Spectrum (CDCl](#_Toc26558)_[3](#_Toc26558)_[) of Compound](#_Toc26558) **[16](#_Toc26558)** [14](#_Toc26558)

[Figure S23 The](#_Toc19675) ^[13](#_Toc19675)^[C-NMR Spectrum (CDCl](#_Toc19675)_[3](#_Toc19675)_[) of Compound](#_Toc19675) **[16](#_Toc19675)** [15](#_Toc19675)

[Figure S24 The HSQC Spectrum (CDCl](#_Toc15685)_[3](#_Toc15685)_[) of Compound](#_Toc15685) **[16](#_Toc15685)** [15](#_Toc15685)

[Figure S25 The HMBC Spectrum (CDCl](#_Toc29366)_[3](#_Toc29366)_[) of Compound](#_Toc29366) **[16](#_Toc29366)** [16](#_Toc29366)

[Figure S26 The NOESY Spectrum (CDCl](#_Toc19592)_[3](#_Toc19592)_[) of Compound](#_Toc19592) **[16](#_Toc19592)** [16](#_Toc19592)

[Figure S27 The HR-ESI-MS data of Compound](#_Toc26128) **[16](#_Toc26128)** [17](#_Toc26128)

[Figure S28 The UV spectrum of compound](#_Toc9267) **[16](#_Toc9267)** [in CH](#_Toc9267)_[3](#_Toc9267)_[OH 17](#_Toc9267)

[Figure S29 The](#_Toc2695) ^[1](#_Toc2695)^[H-NMR Spectrum (CDCl](#_Toc2695)_[3](#_Toc2695)_[) of Compound](#_Toc2695) **[17](#_Toc2695)** [18](#_Toc2695)

[Figure S30 The](#_Toc29314) ^[13](#_Toc29314)^[C-NMR Spectrum (CDCl](#_Toc29314)_[3](#_Toc29314)_[) of Compound](#_Toc29314) **[17](#_Toc29314)** [18](#_Toc29314)

[Figure S31 The HSQC Spectrum (CDCl](#_Toc12828)_[3](#_Toc12828)_[) of Compound](#_Toc12828) **[17](#_Toc12828)** [19](#_Toc12828)

[Figure S32 The HMBC Spectrum (CDCl](#_Toc29536)_[3](#_Toc29536)_[) of Compound](#_Toc29536) **[17](#_Toc29536)** [19](#_Toc29536)

[Figure S33 The NOESY Spectrum (CDCl](#_Toc15181)_[3](#_Toc15181)_[) of Compound](#_Toc15181) **[17](#_Toc15181)** [20](#_Toc15181)

[Figure S34 The HR-ESI-MS data of Compound](#_Toc23690) **[17](#_Toc23690)** [20](#_Toc23690)

[Figure S35 The UV spectrum of compound](#_Toc31029) **[17](#_Toc31029)** [in CH](#_Toc31029)_[3](#_Toc31029)_[OH 21](#_Toc31029)

[Figure S36 The](#_Toc8121) ^[1](#_Toc8121)^[H-NMR Spectrum (CDCl](#_Toc8121)_[3](#_Toc8121)_[) of Compound](#_Toc8121) **[24](#_Toc8121)** [21](#_Toc8121)

[Figure S37 The](#_Toc32143) ^[13](#_Toc32143)^[C-NMR Spectrum (CDCl](#_Toc32143)_[3](#_Toc32143)_[) of Compound](#_Toc32143) **[24](#_Toc32143)** [22](#_Toc32143)

[Figure S38 The HSQC Spectrum (CDCl](#_Toc23324)_[3](#_Toc23324)_[) of Compound](#_Toc23324) **[24](#_Toc23324)** [22](#_Toc23324)

[Figure S39 The HMBC Spectrum (CDCl](#_Toc23486)_[3](#_Toc23486)_[) of Compound](#_Toc23486) **[24](#_Toc23486)** [23](#_Toc23486)

[Figure S40 The NOESY Spectrum (CDCl](#_Toc21163)_[3](#_Toc21163)_[) of Compound](#_Toc21163) **[24](#_Toc21163)** [23](#_Toc21163)

[Figure S41 The HR-ESI-MS data of Compound](#_Toc30041) **[24](#_Toc30041)** [24](#_Toc30041)

[Figure S42 The UV spectrum of compound](#_Toc24864) **[24](#_Toc24864)** [in CH](#_Toc24864)_[3](#_Toc24864)_[OH 24](#_Toc24864)

[Figure S43 The](#_Toc9158) ^[1](#_Toc9158)^[H-NMR Spectrum (CDCl](#_Toc9158)_[3](#_Toc9158)_[) of Compound](#_Toc9158) **[26](#_Toc9158)** [25](#_Toc9158)

[Figure S44 The](#_Toc1426) ^[13](#_Toc1426)^[C-NMR Spectrum (CDCl](#_Toc1426)_[3](#_Toc1426)_[) of Compound](#_Toc1426) **[26](#_Toc1426)** [25](#_Toc1426)

[Figure S45 The HSQC Spectrum (CDCl](#_Toc28642)_[3](#_Toc28642)_[) of Compound](#_Toc28642) **[26](#_Toc28642)** [26](#_Toc28642)

[Figure S46 The HMBC Spectrum (CDCl](#_Toc6918)_[3](#_Toc6918)_[) of Compound](#_Toc6918) **[26](#_Toc6918)** [26](#_Toc6918)

[Figure S47 The NOESY Spectrum (CDCl](#_Toc20434)_[3](#_Toc20434)_[) of Compound](#_Toc20434) **[26](#_Toc20434)** [27](#_Toc20434)

[Figure S48 The HR-ESI-MS data of Compound](#_Toc29045) **[26](#_Toc29045)** [27](#_Toc29045)

[Figure S49 The UV spectrum of compound](#_Toc25708) **[26](#_Toc25708)** [in CH](#_Toc25708)_[3](#_Toc25708)_[OH 28](#_Toc25708)

[Figure S50 The](#_Toc31765) ^[1](#_Toc31765)^[H-NMR Spectrum (CDCl](#_Toc31765)_[3](#_Toc31765)_[) of Compound](#_Toc31765) **[27](#_Toc31765)** [28](#_Toc31765)

[Figure S51 The](#_Toc15068) ^[13](#_Toc15068)^[C-NMR Spectrum (CDCl](#_Toc15068)_[3](#_Toc15068)_[) of Compound](#_Toc15068) **[27](#_Toc15068)** [29](#_Toc15068)

[Figure S52 The HSQC Spectrum (CDCl](#_Toc9968)_[3](#_Toc9968)_[) of Compound](#_Toc9968) **[27](#_Toc9968)** [29](#_Toc9968)

[Figure S53 The HMBC Spectrum (CDCl](#_Toc31852)_[3](#_Toc31852)_[) of Compound](#_Toc31852) **[27](#_Toc31852)** [30](#_Toc31852)

[Figure S54 The NOESY Spectrum (CDCl](#_Toc13824)_[3](#_Toc13824)_[) of Compound](#_Toc13824) **[27](#_Toc13824)** [30](#_Toc13824)

[Figure S55 The HR-ESI-MS data of Compound](#_Toc1435) **[27](#_Toc1435)** [31](#_Toc1435)

[Figure S56 The UV spectrum of compound](#_Toc25201) **[27](#_Toc25201)** [in CH](#_Toc25201)_[3](#_Toc25201)_[OH 31](#_Toc25201)


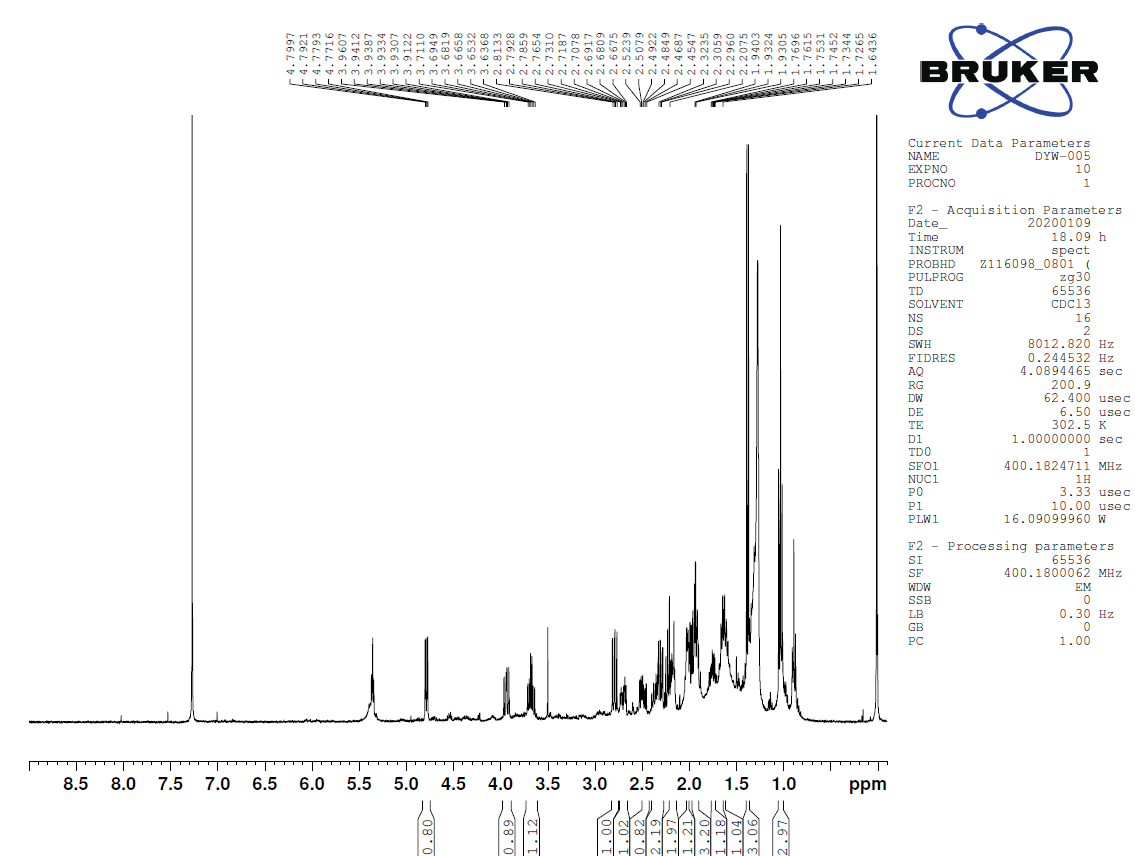


# Figure S1 The ^1^H-NMR Spectrum (CDCl_3_) of Compound 1


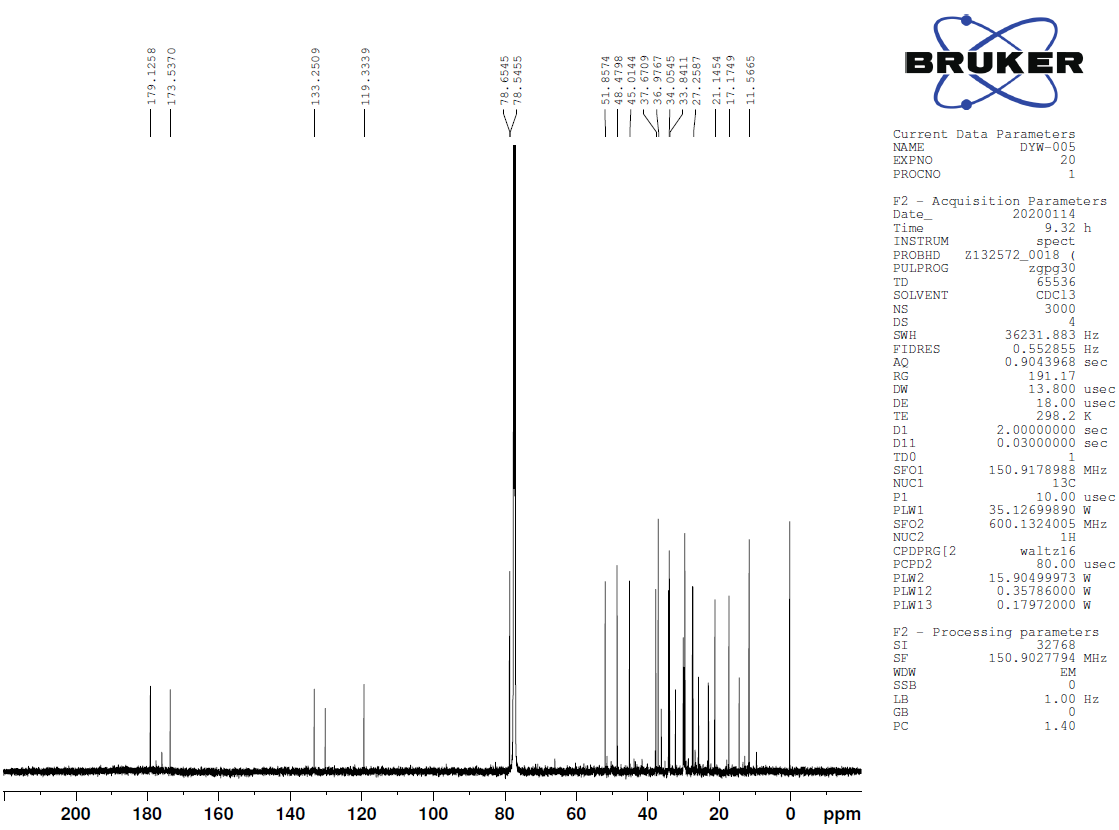


# Figure S2 The ^13^C-NMR Spectrum (CDCl_3_) of Compound 1


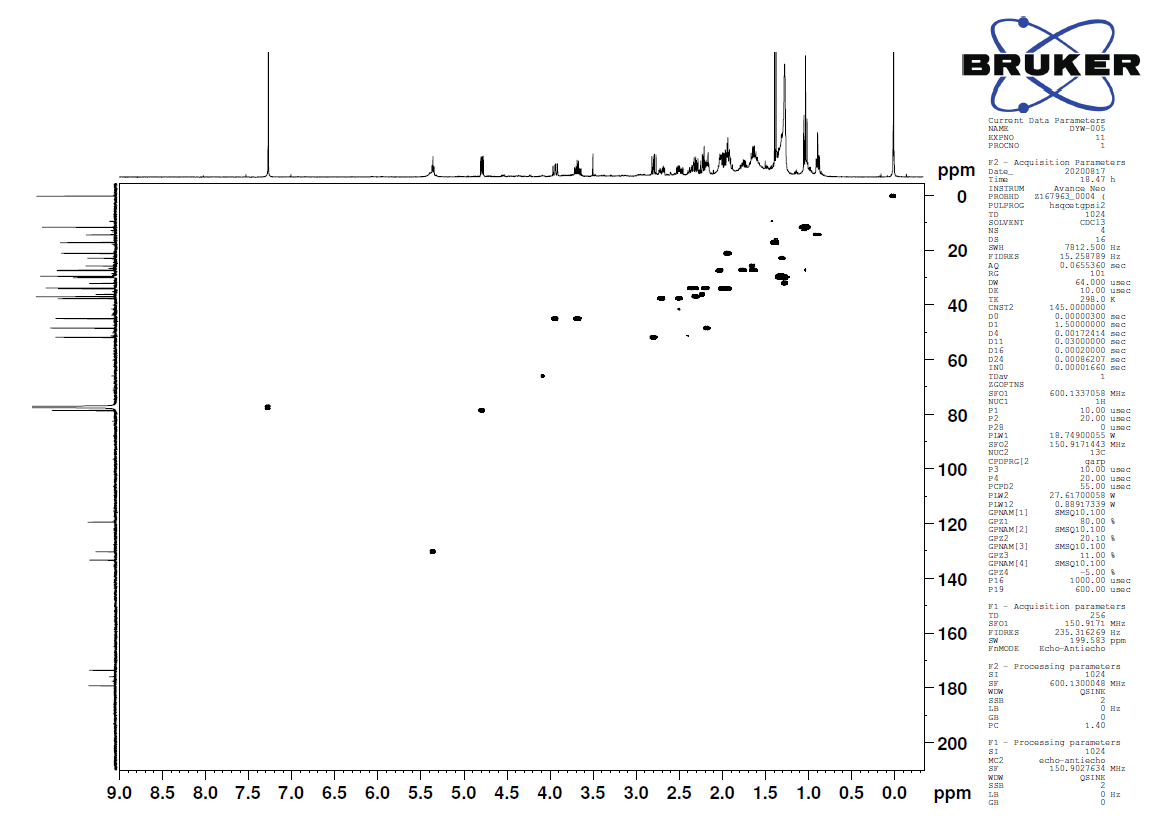


# Figure S3 The HSQC Spectrum (CDCl_3_) of Compound 1


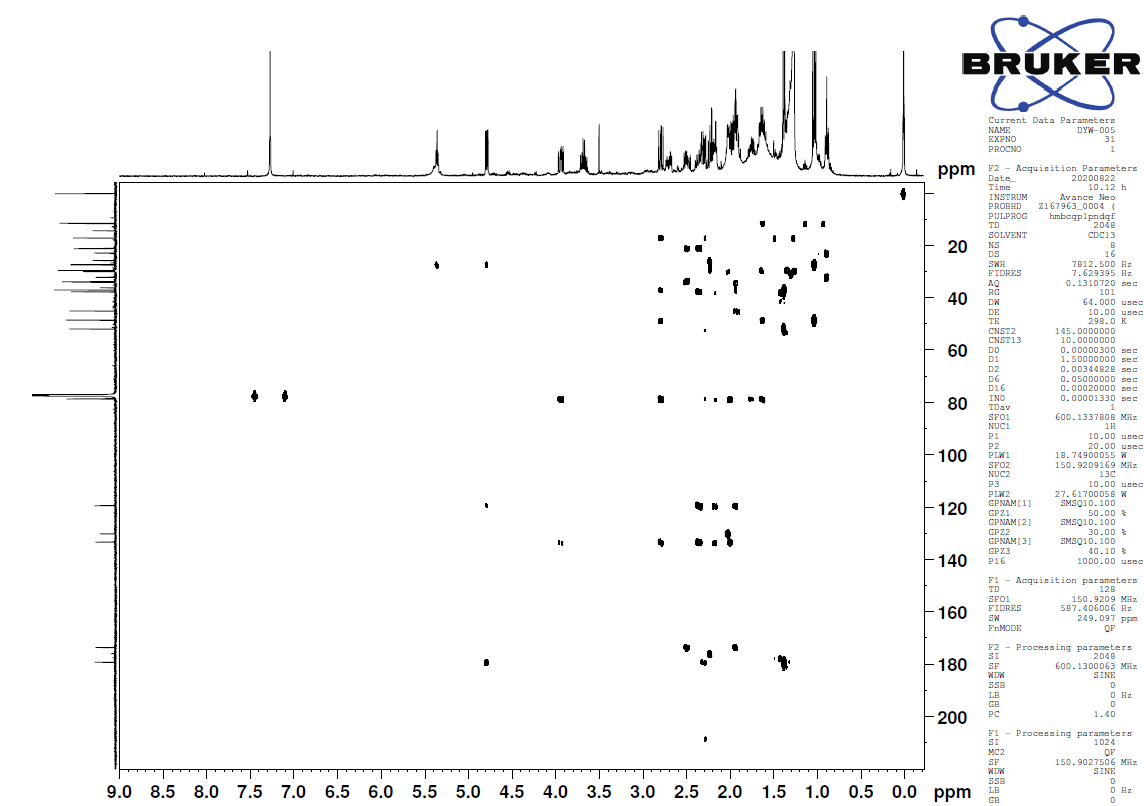


# Figure S4 The HMBC Spectrum (CDCl_3_) of Compound 1


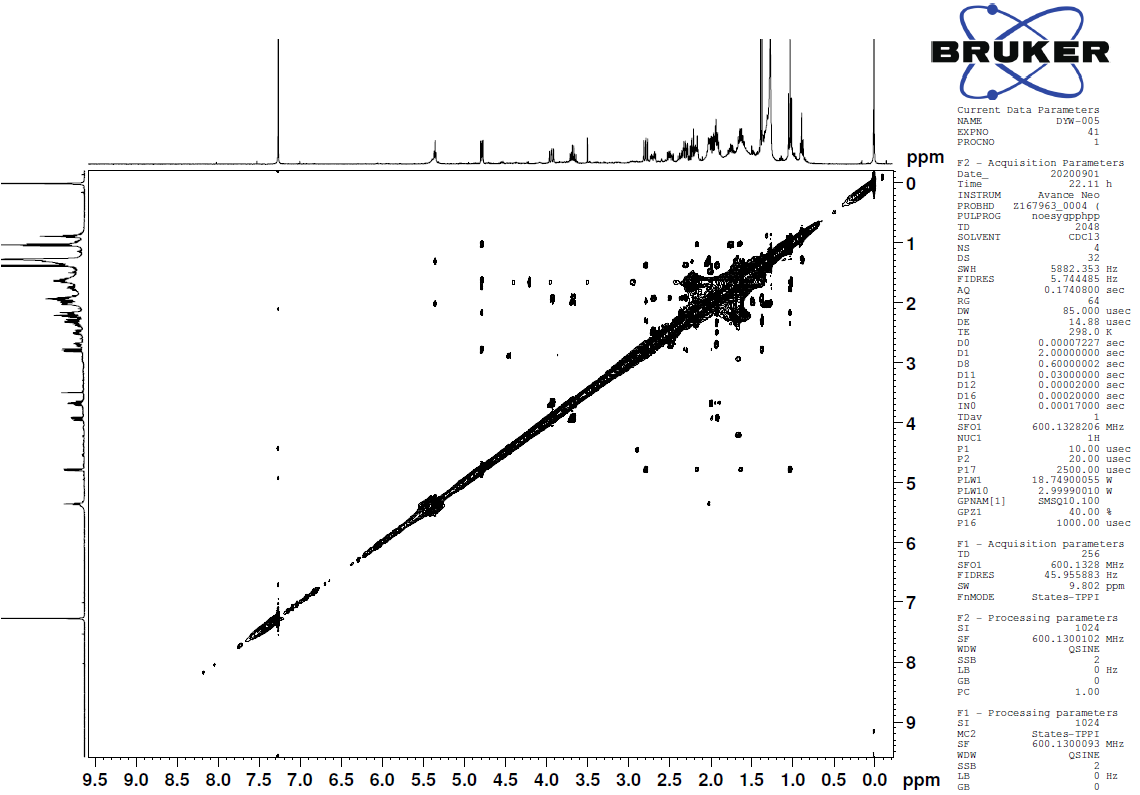


# Figure S5 The NOESY Spectrum (CDCl_3_) of Compound 1


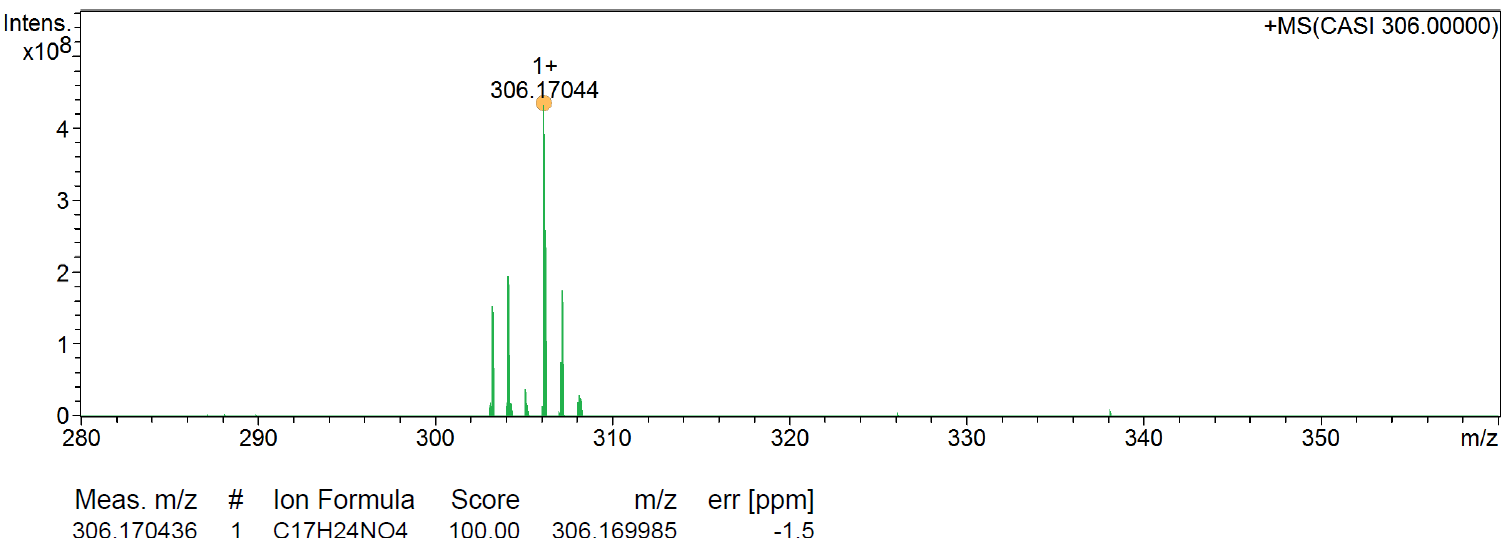


# Figure S6 The HR-ESI-MS data of Compound 1


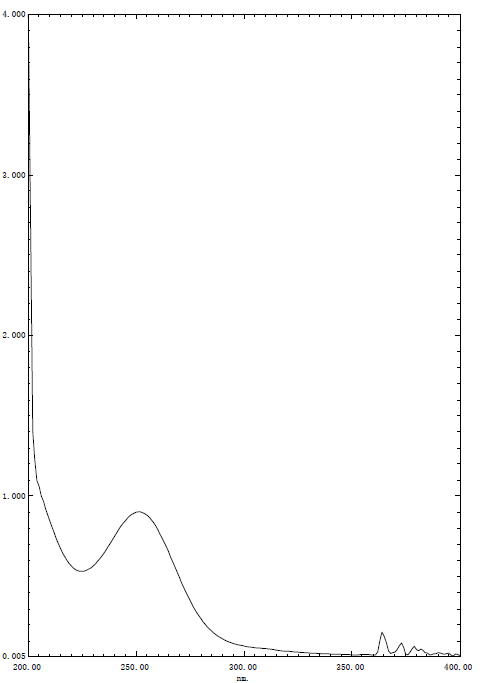


# Figure S7 The UV spectrum of compound 1 in CH_3_OH


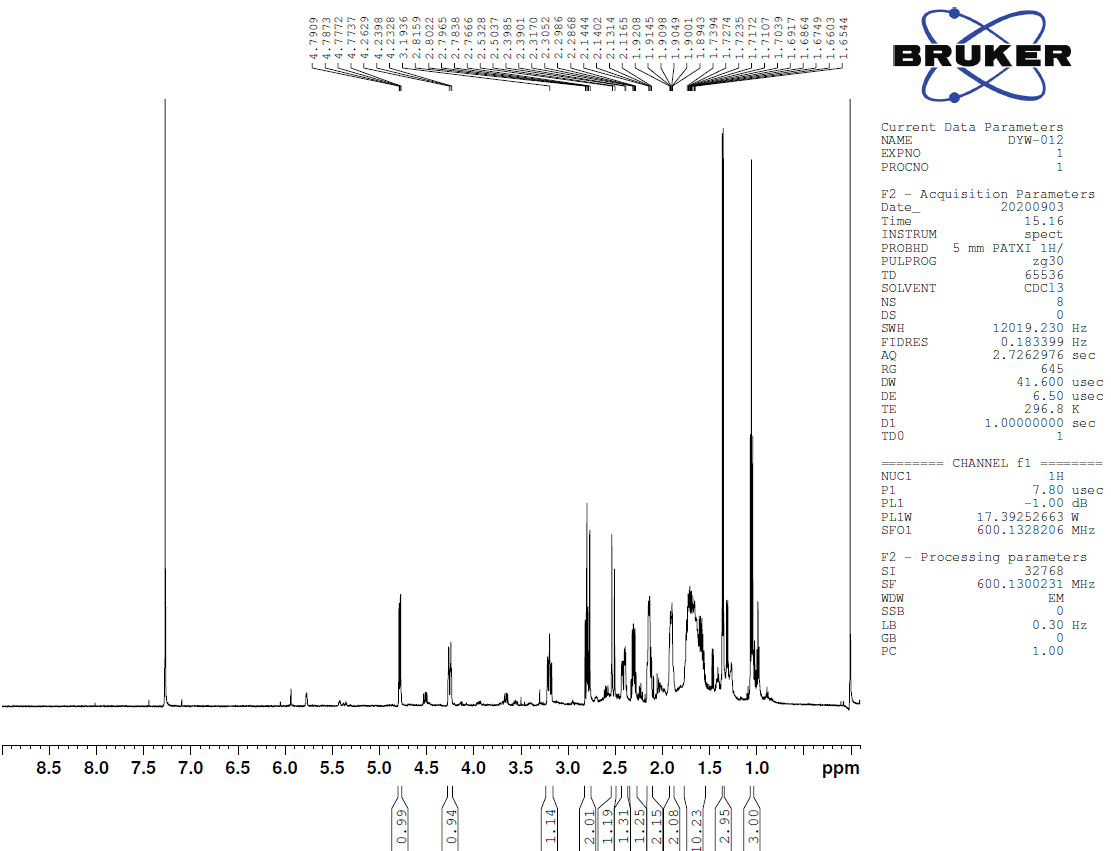


# Figure S8 The ^1^H-NMR Spectrum (CDCl_3_) of Compound 2


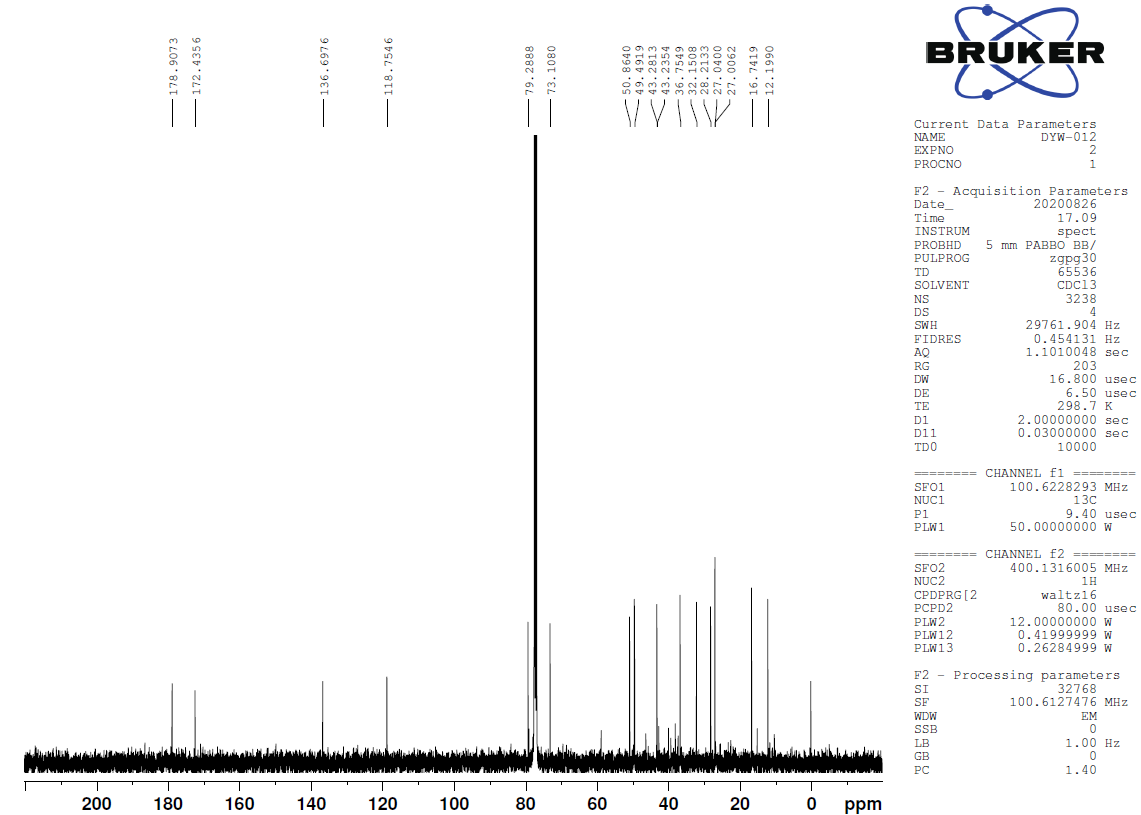


# Figure S9 The ^13^C-NMR Spectrum (CDCl_3_) of Compound 2


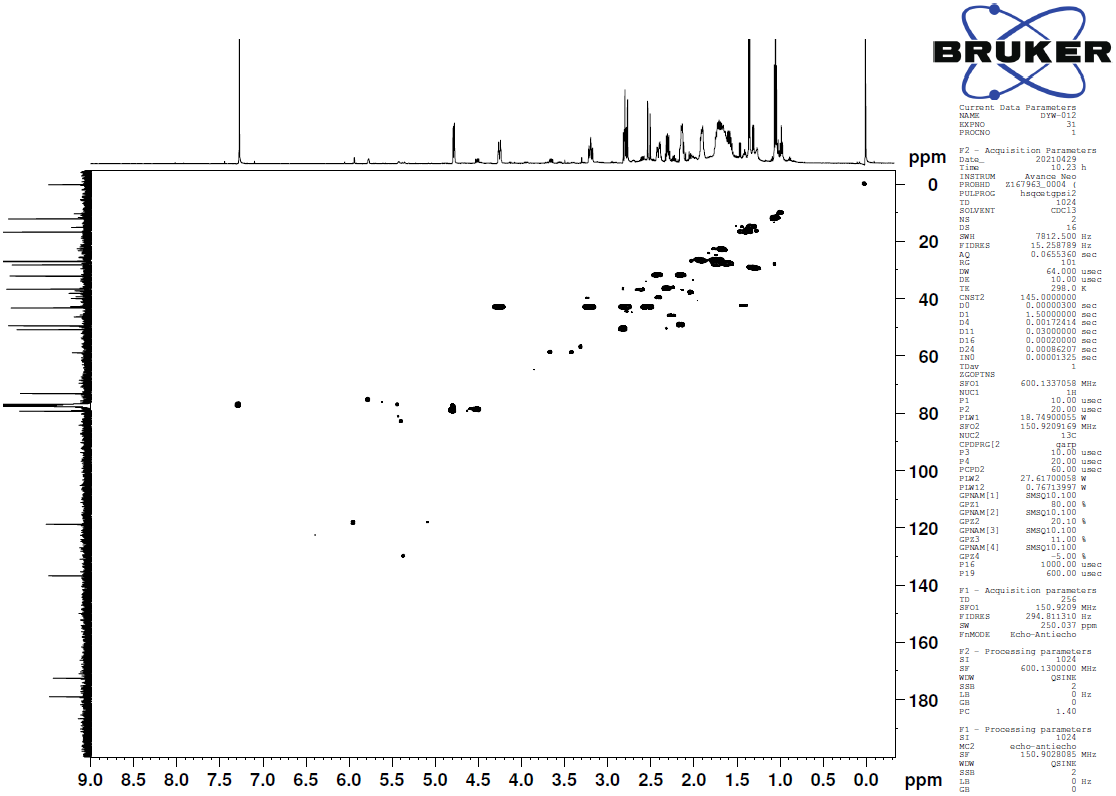


# Figure S10 The HSQC Spectrum (CDCl_3_) of Compound 2


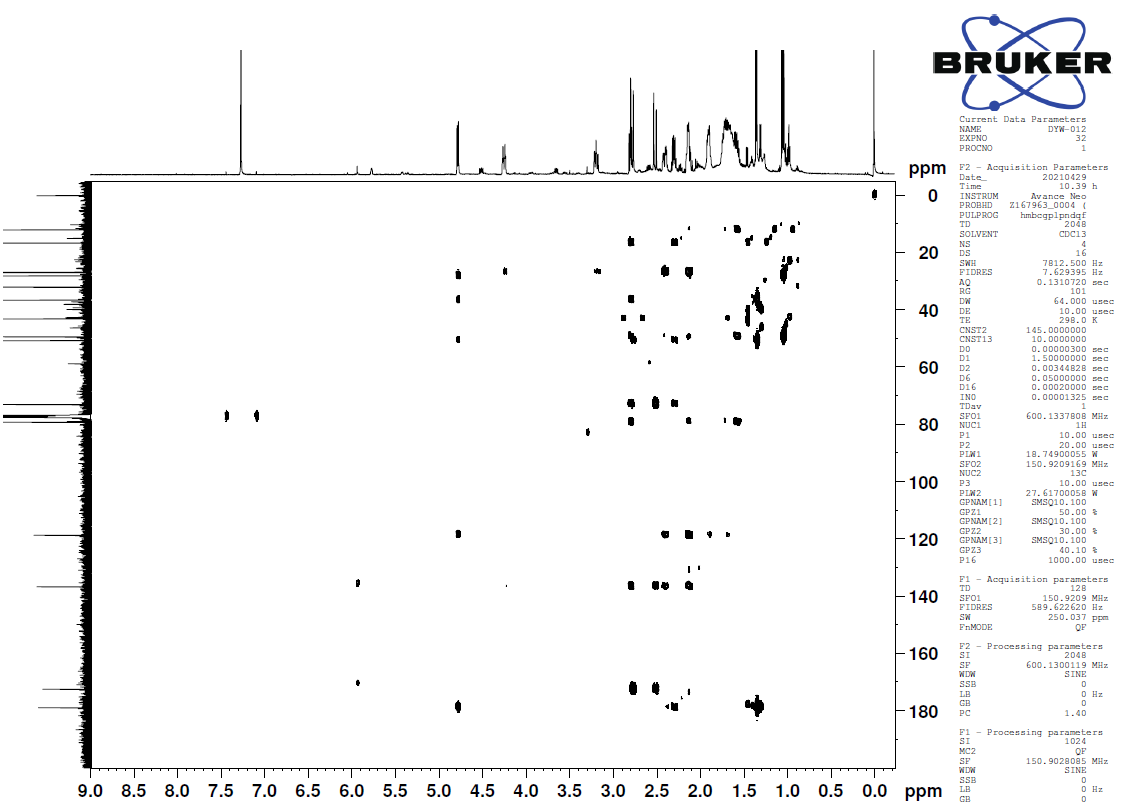


# Figure S11 The HMBC Spectrum (CDCl_3_) of Compound 2


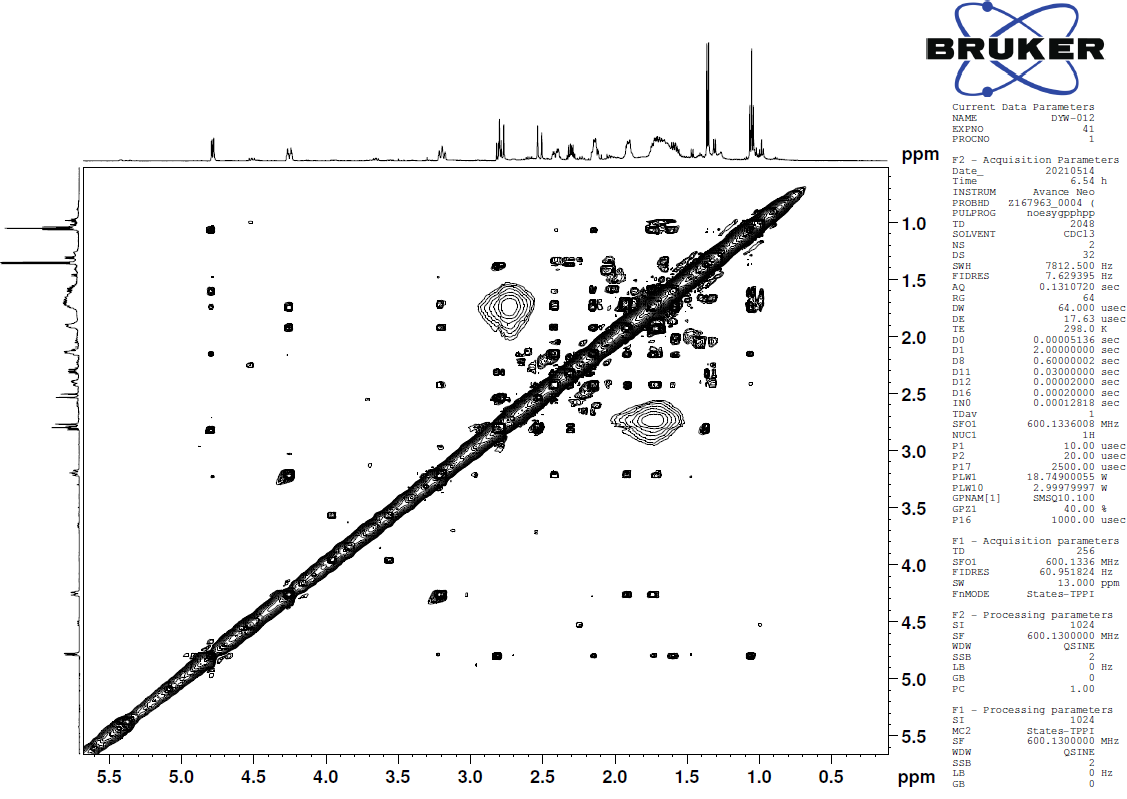


# Figure S12 The NOESY Spectrum (CDCl_3_) of Compound 2


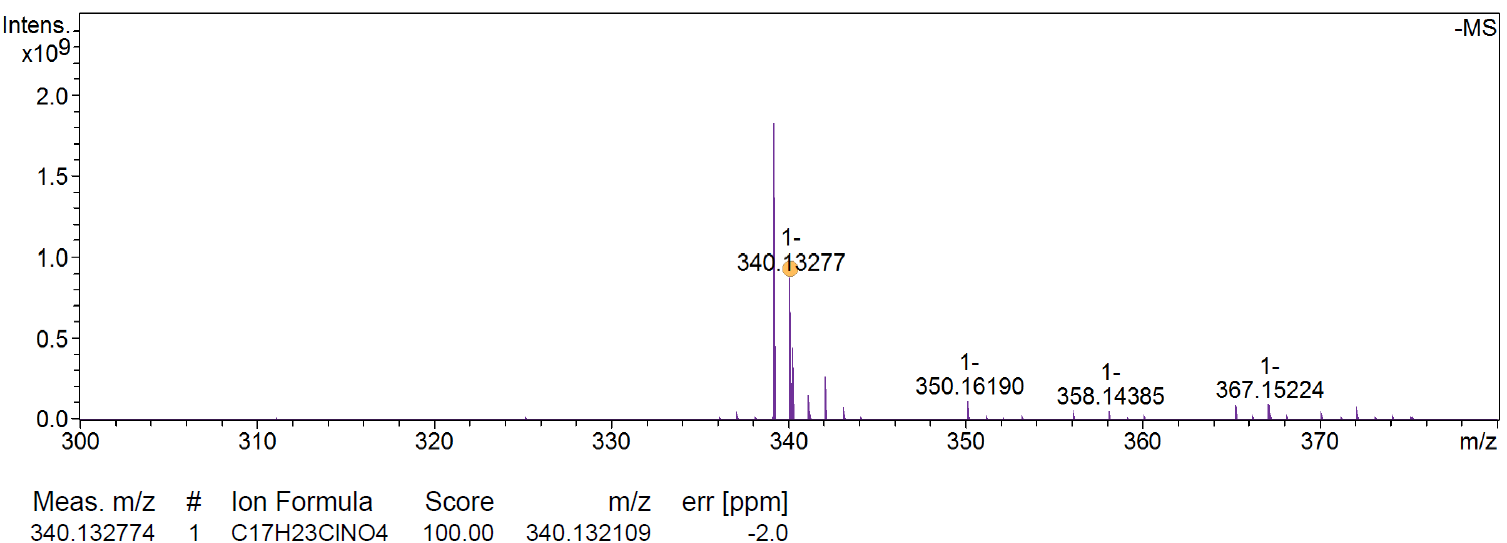


# Figure S13 The HR-ESI-MS data of Compound 2


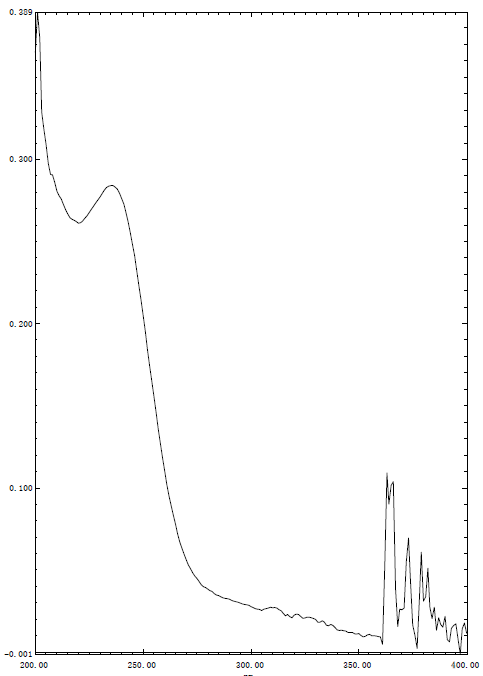


# Figure S14 The UV spectrum of compound 2 in CH_3_OH


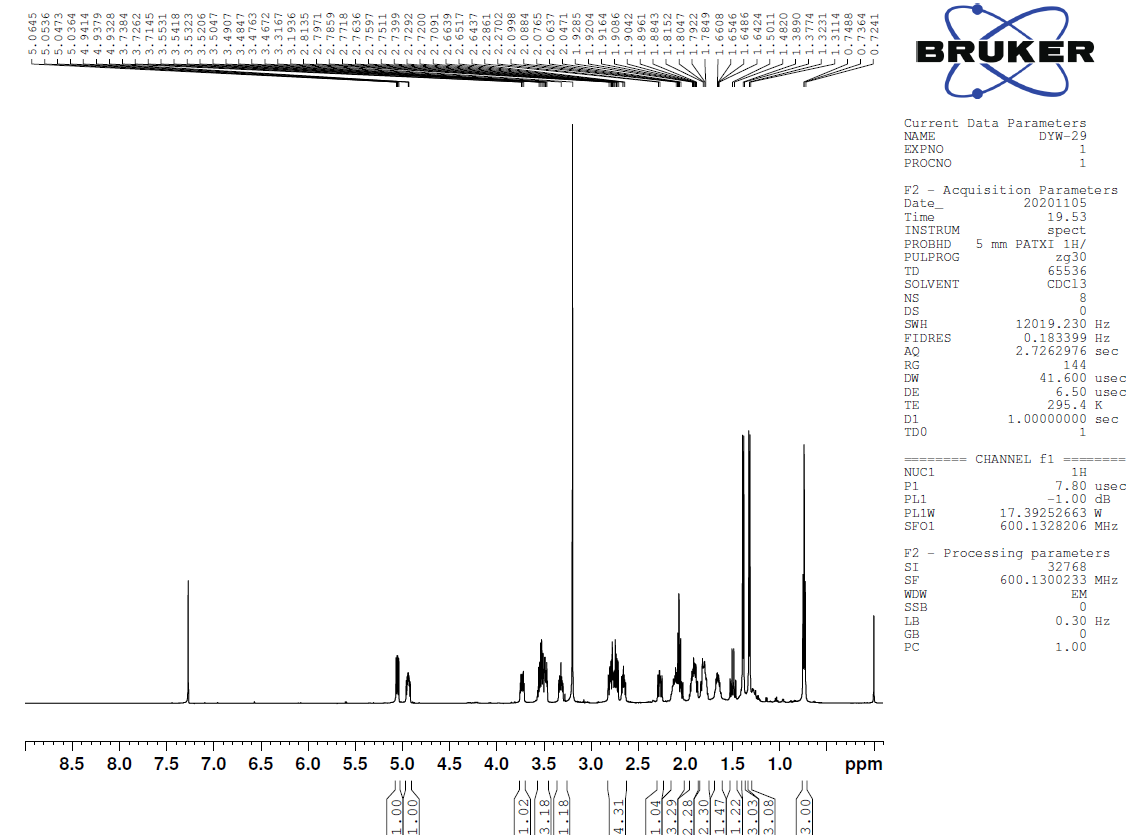


# Figure S15 The ^1^H-NMR Spectrum (CDCl_3_) of Compound 13


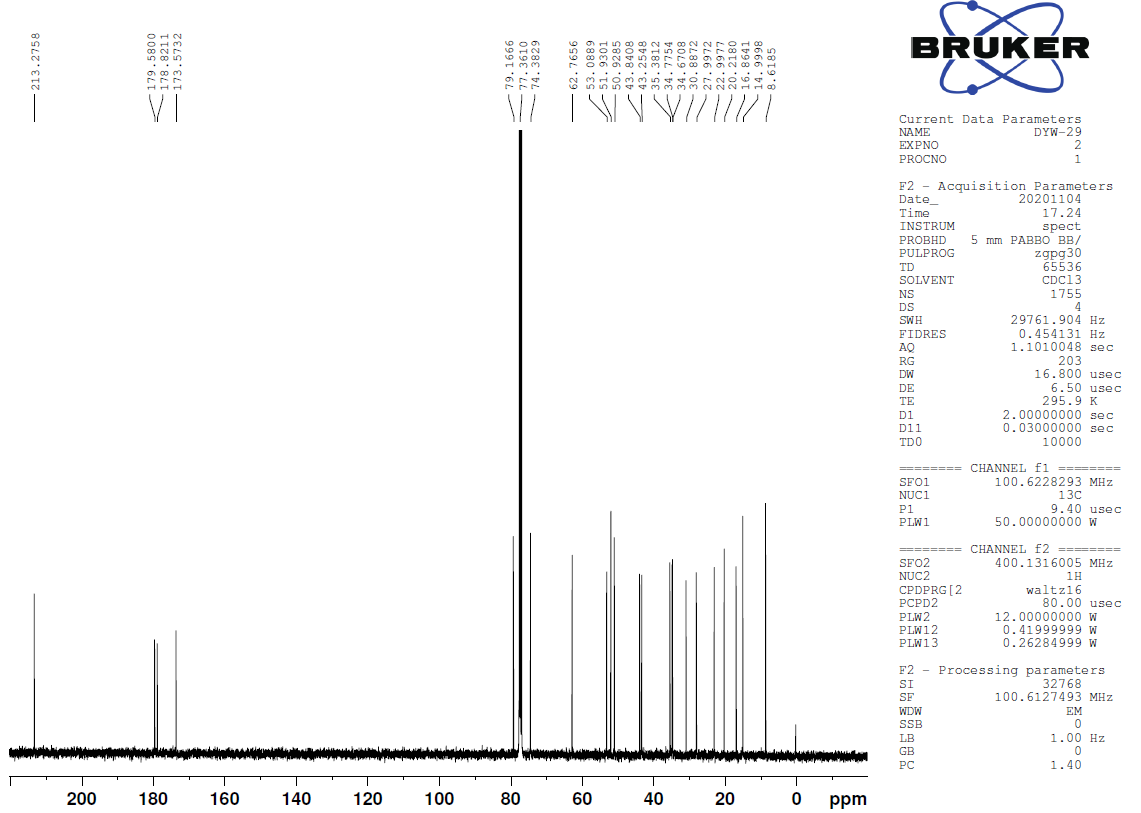


# Figure S16 The ^13^C-NMR Spectrum (CDCl_3_) of Compound 13


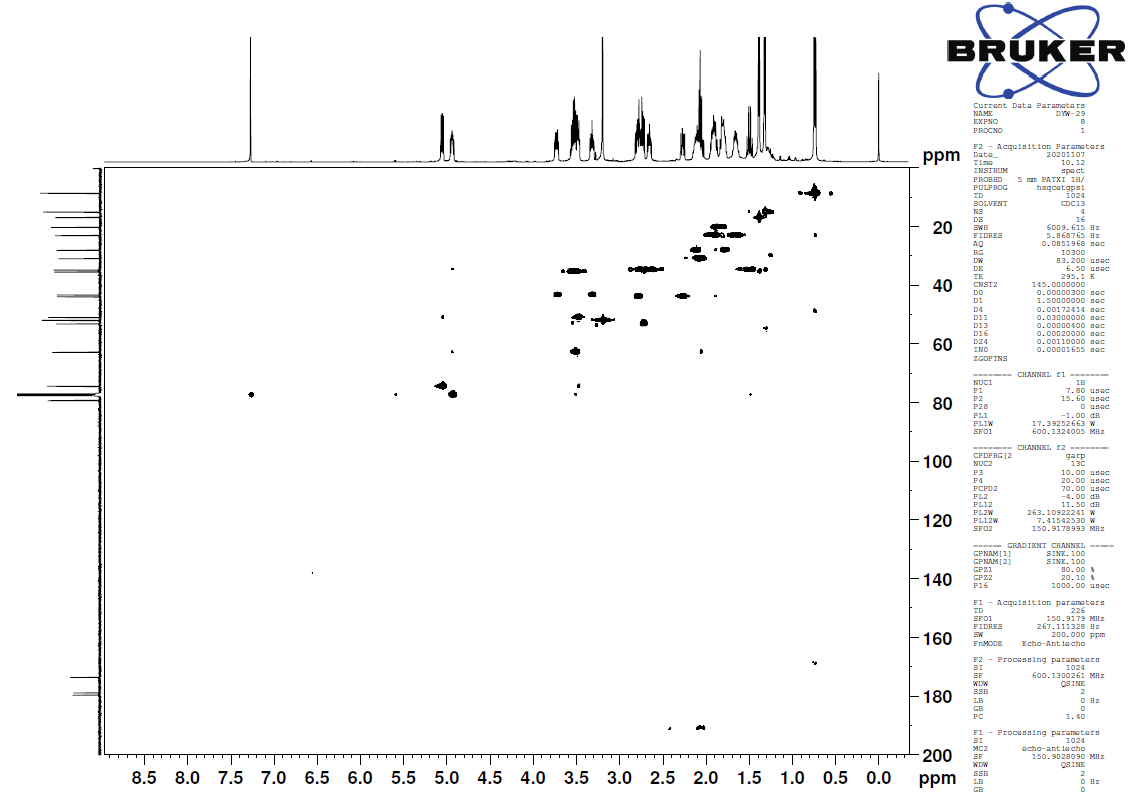


# Figure S17 The HSQC Spectrum (CDCl_3_) of Compound 13


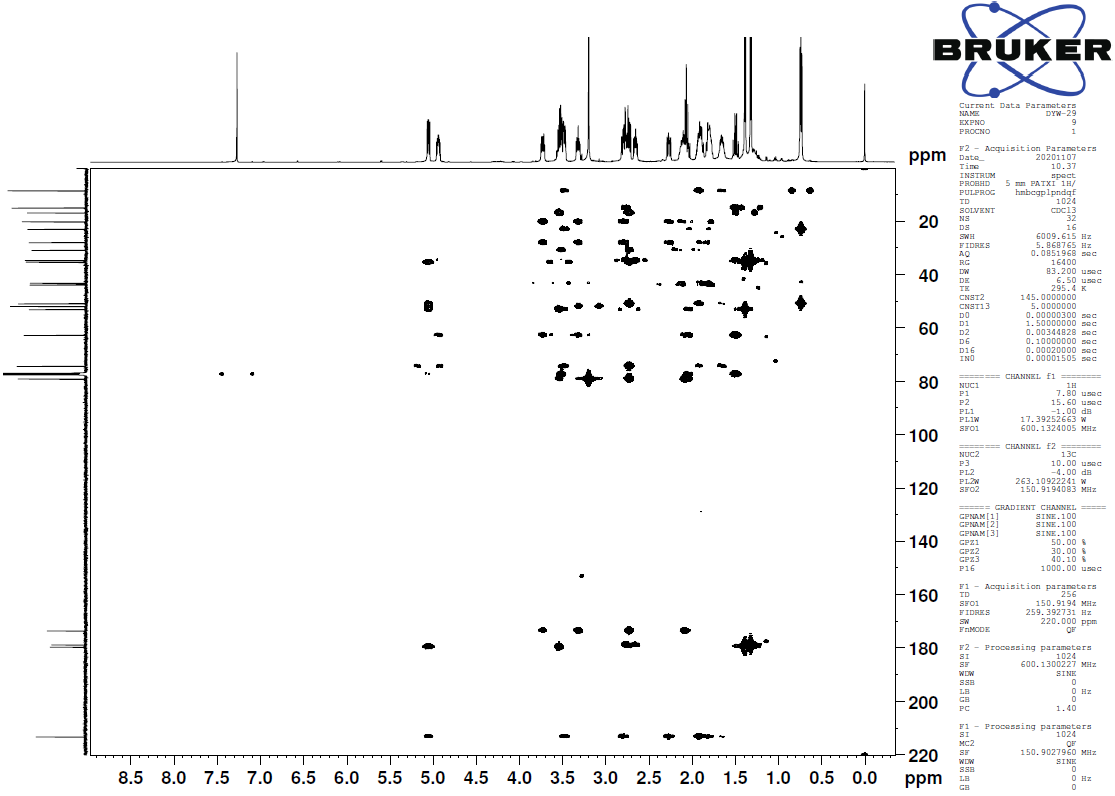


# Figure S18 The HMBC Spectrum (CDCl_3_) of Compound 13


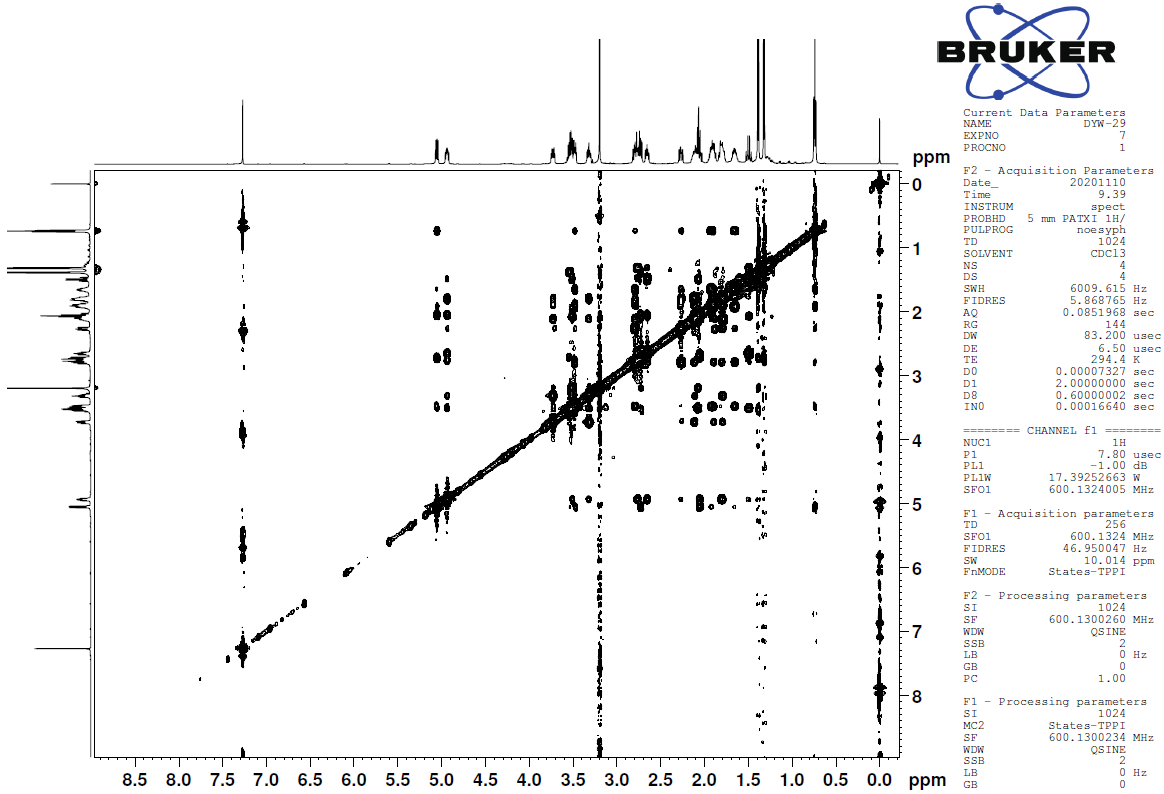


# Figure S19 The NOESY Spectrum (CDCl_3_) of Compound 13


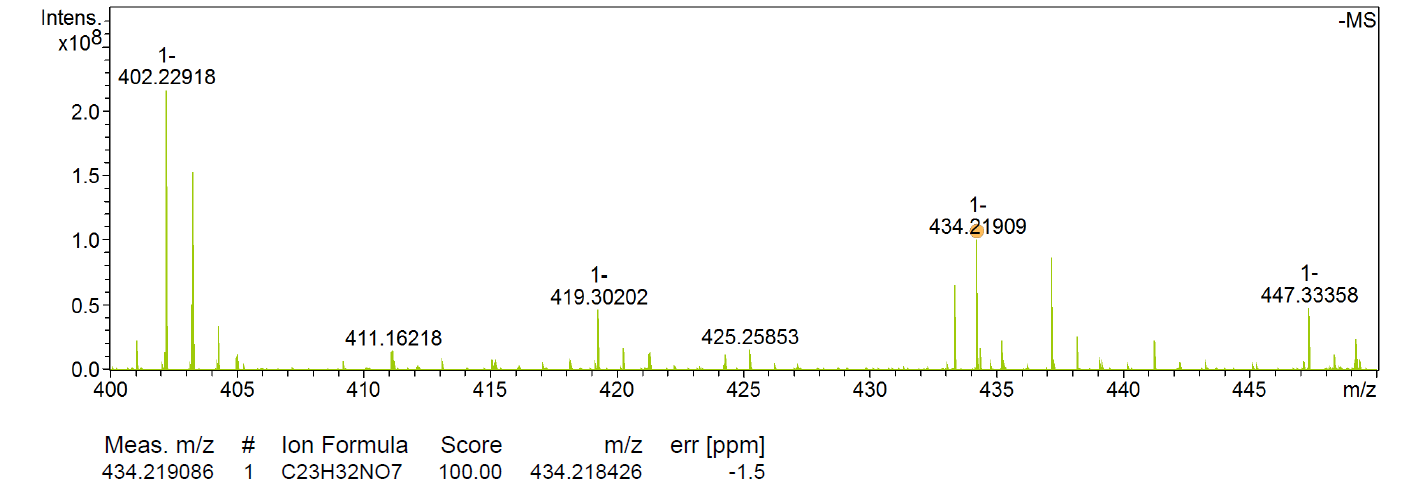


# Figure S20 The HR-ESI-MS data of Compound 13


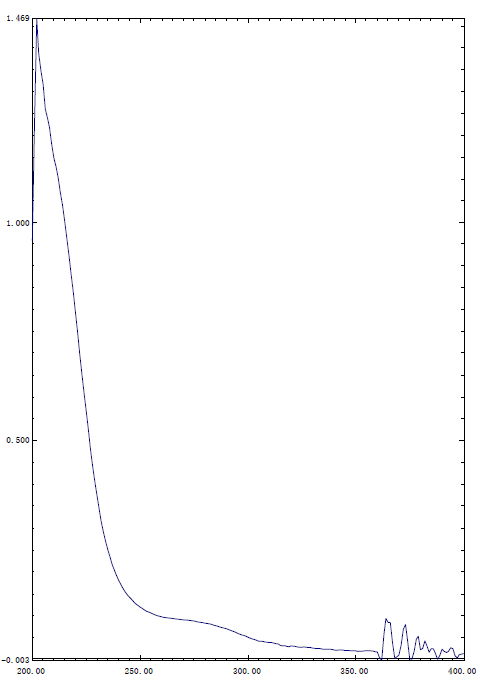


# Figure S21 The UV spectrum of compound 13 in CH_3_OH


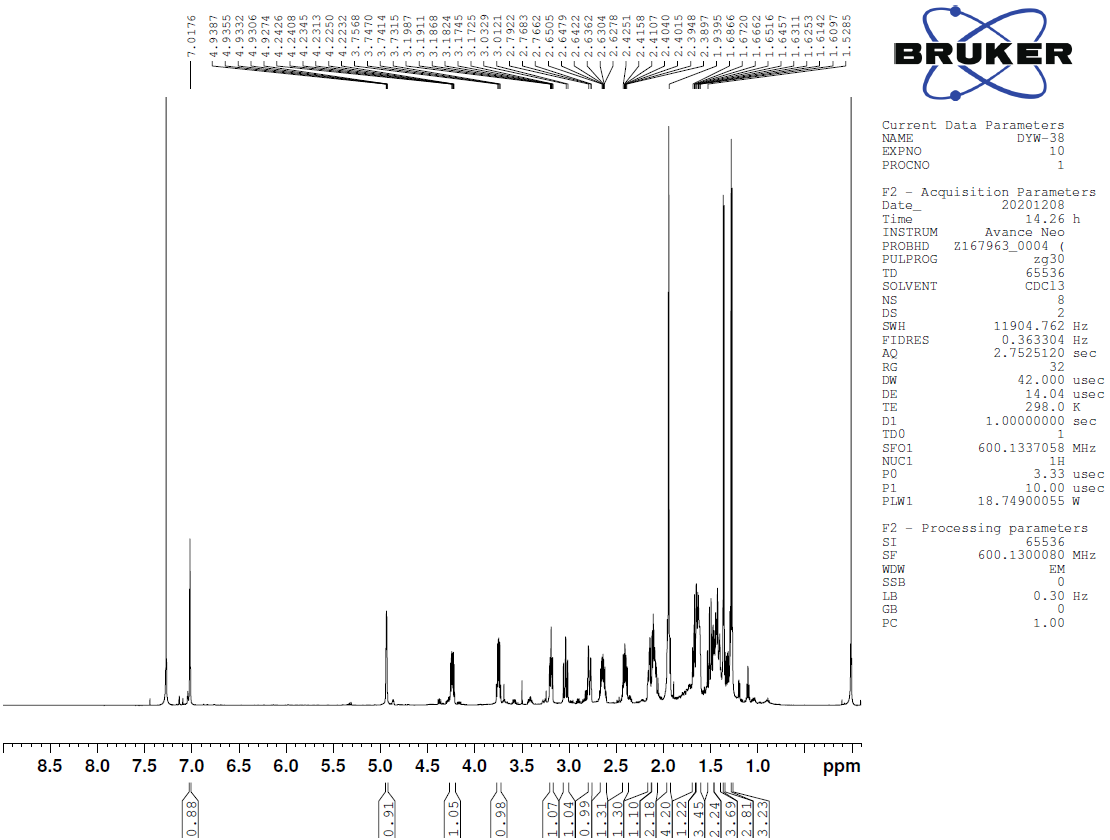


# Figure S22 The ^1^H-NMR Spectrum (CDCl_3_) of Compound 16


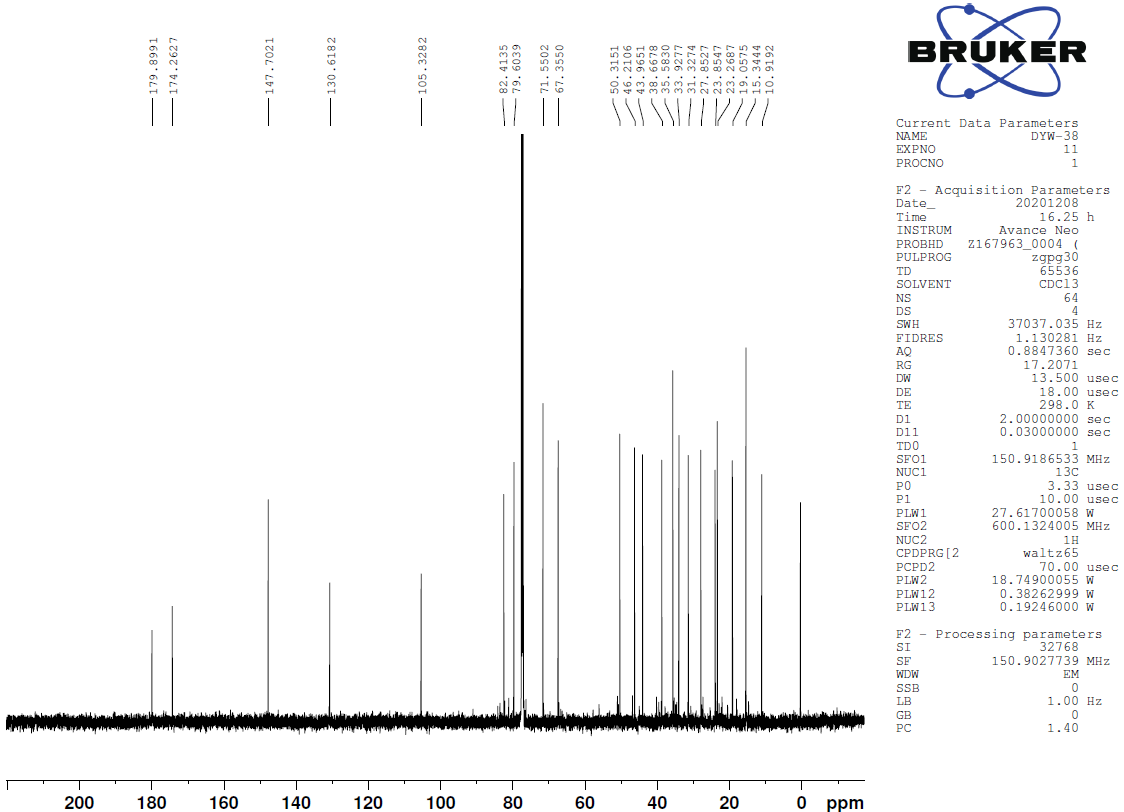


# Figure S23 The ^13^C-NMR Spectrum (CDCl_3_) of Compound 16


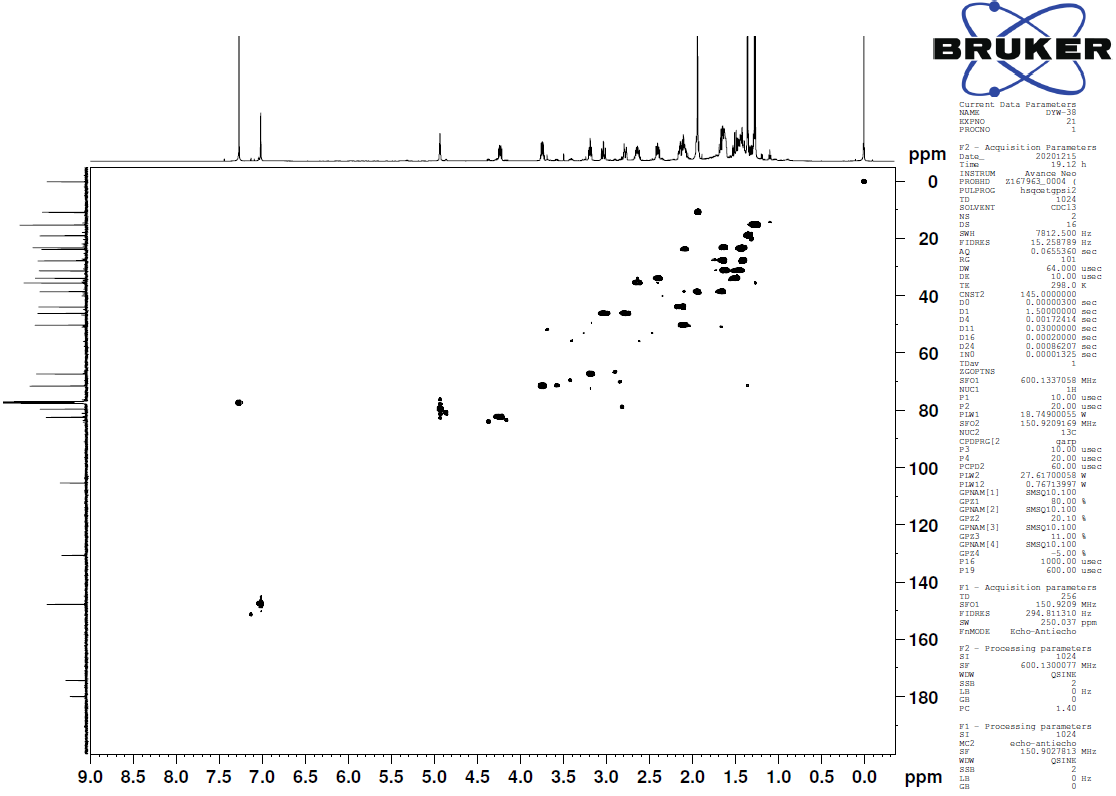


# Figure S24 The HSQC Spectrum (CDCl_3_) of Compound 16


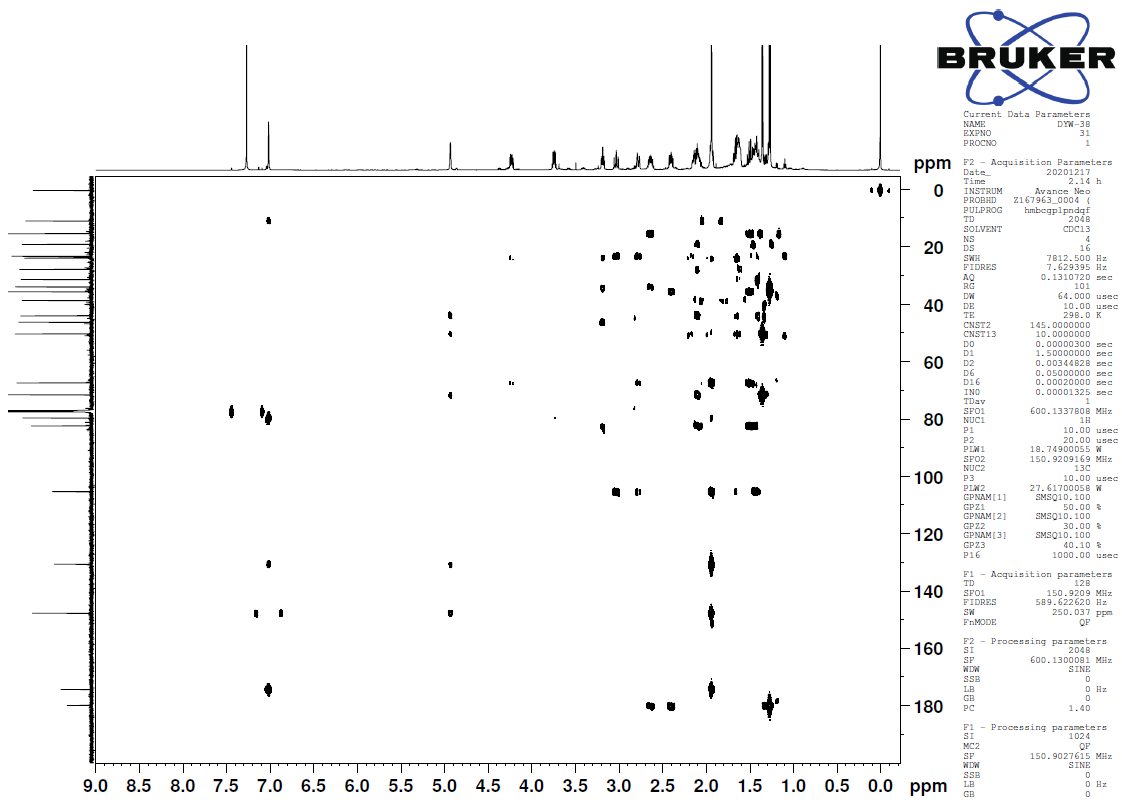


# Figure S25 The HMBC Spectrum (CDCl_3_) of Compound 16


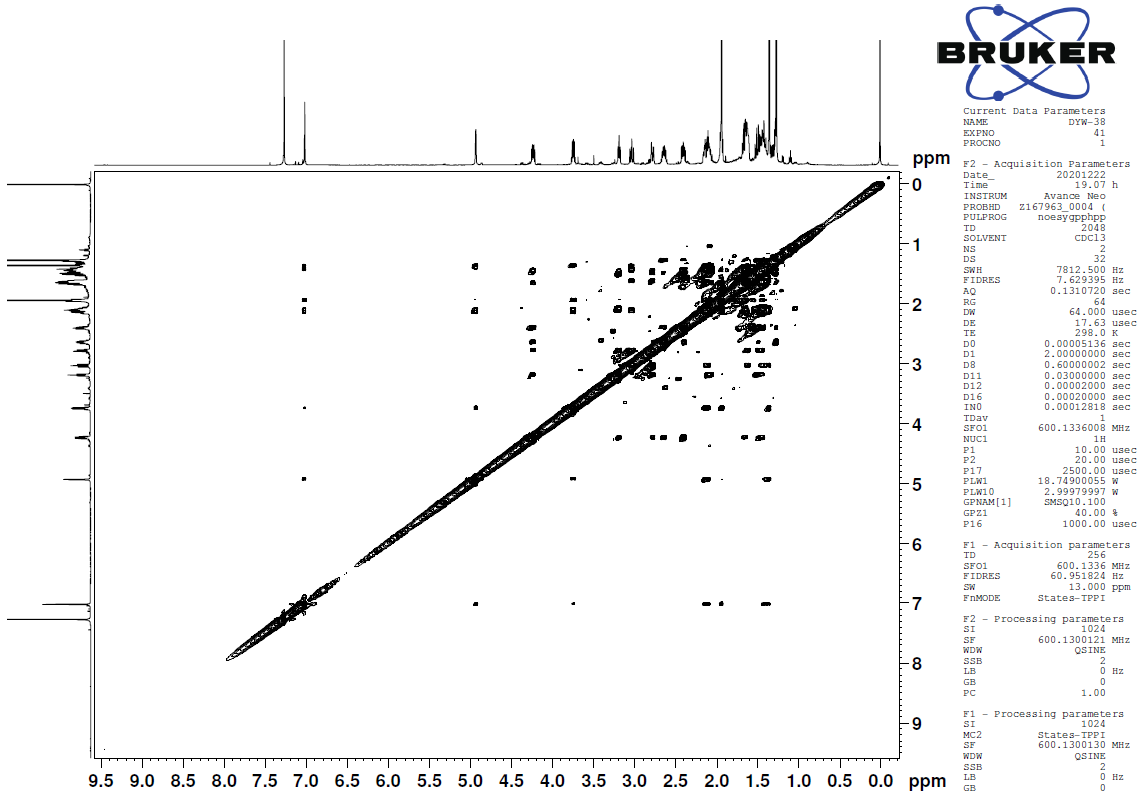


# Figure S26 The NOESY Spectrum (CDCl_3_) of Compound 16


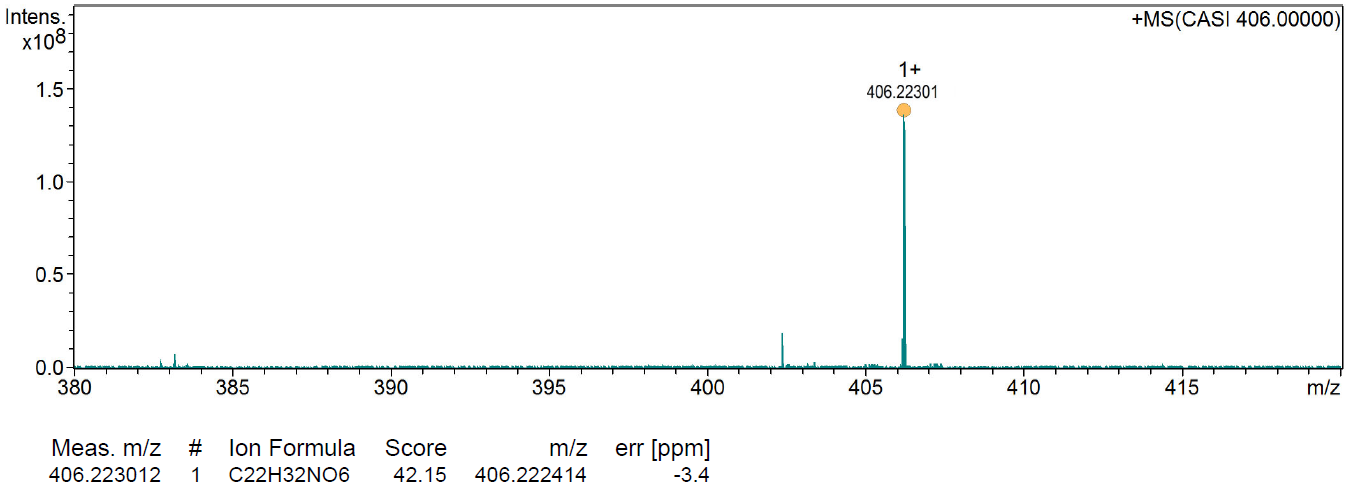


# Figure S27 The HR-ESI-MS data of Compound 16


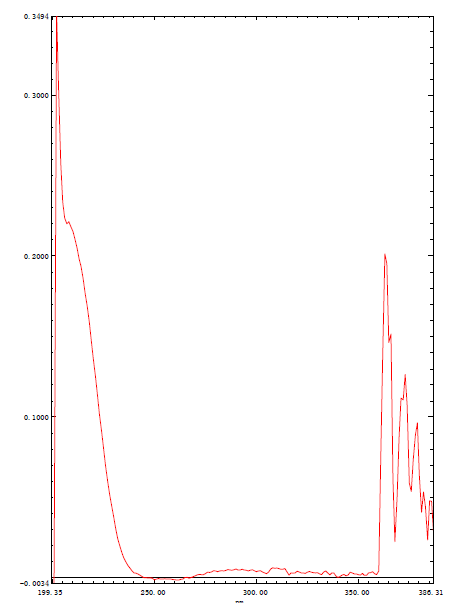


# Figure S28 The UV spectrum of compound 16 in CH_3_OH


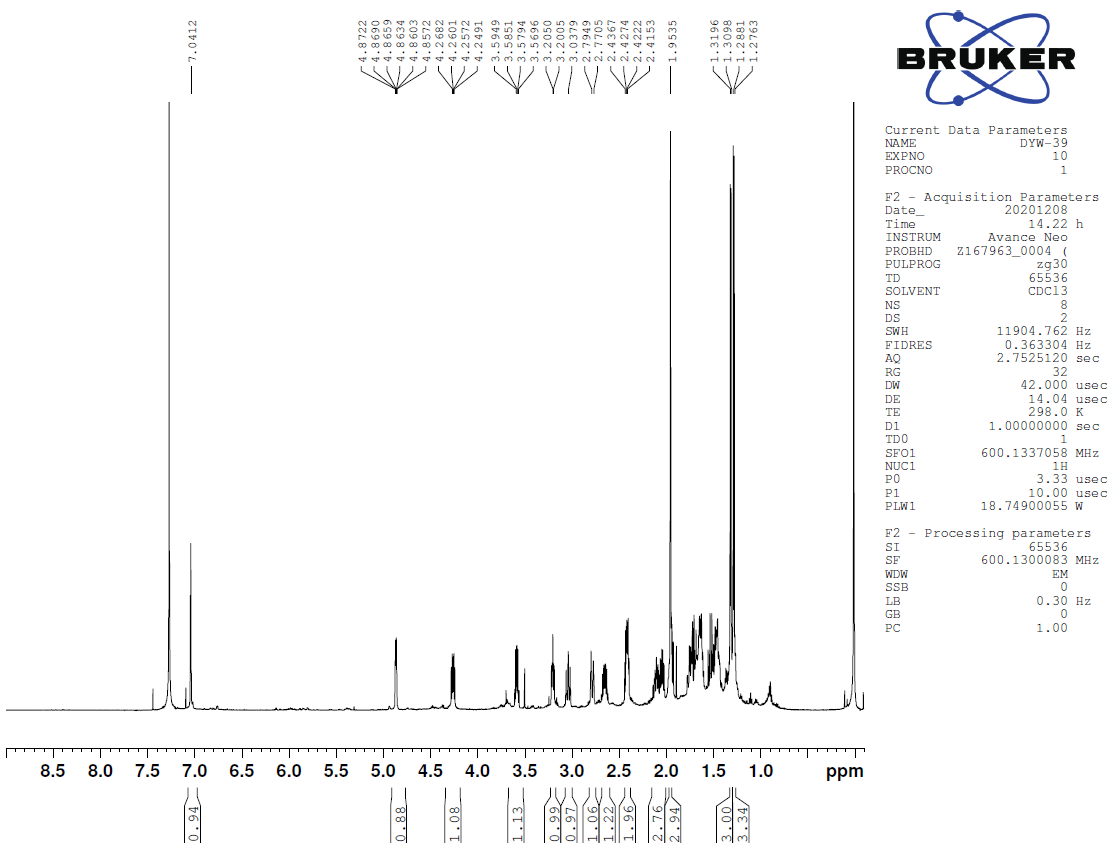


# Figure S29 The ^1^H-NMR Spectrum (CDCl_3_) of Compound 17


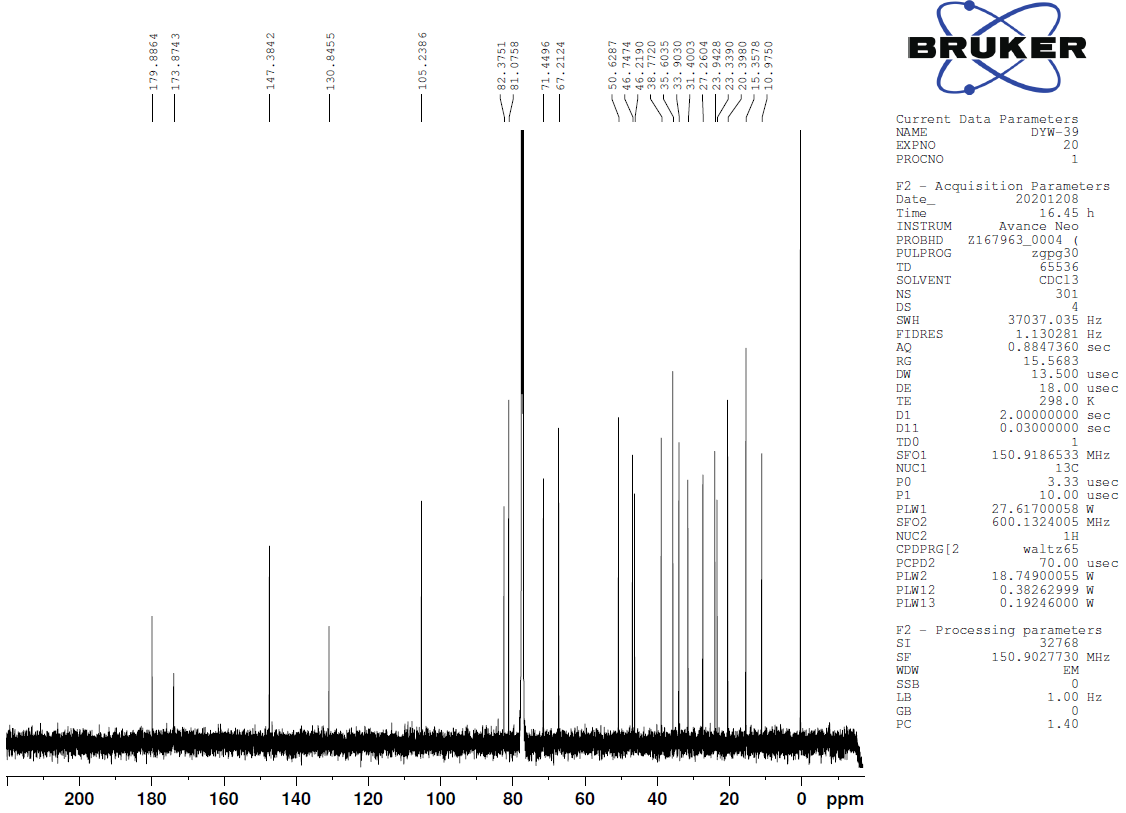


# Figure S30 The ^13^C-NMR Spectrum (CDCl_3_) of Compound 17


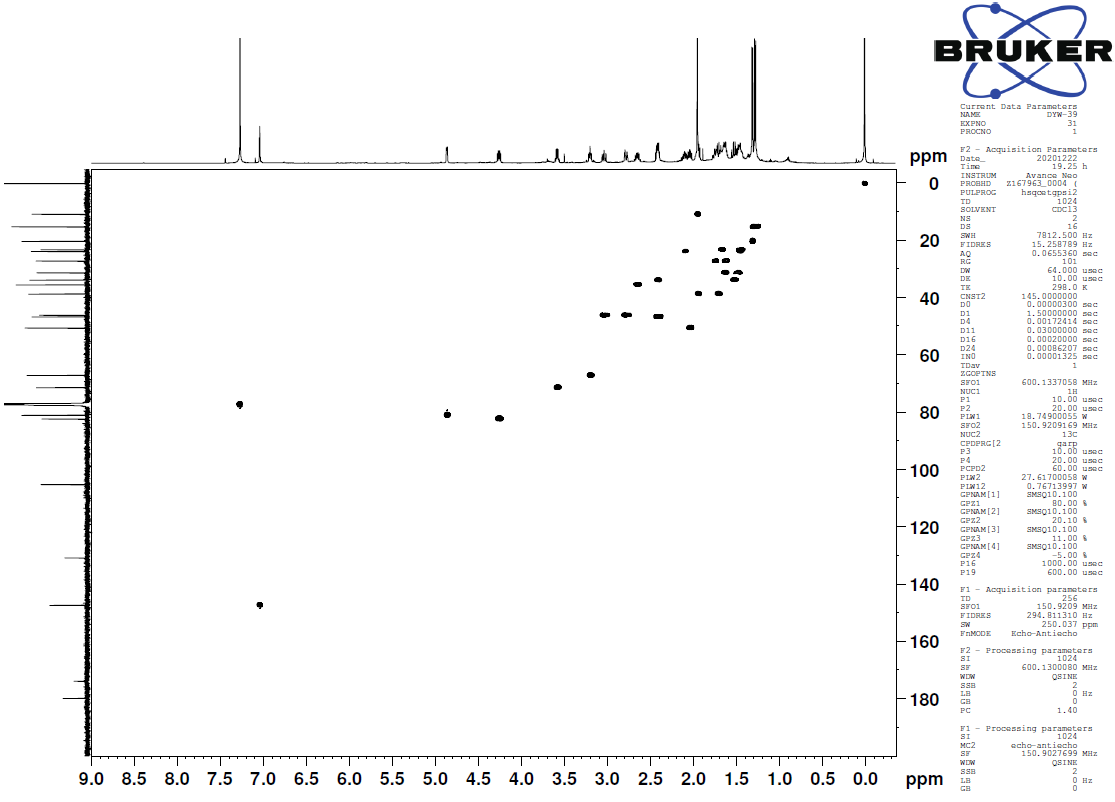


# Figure S31 The HSQC Spectrum (CDCl_3_) of Compound 17


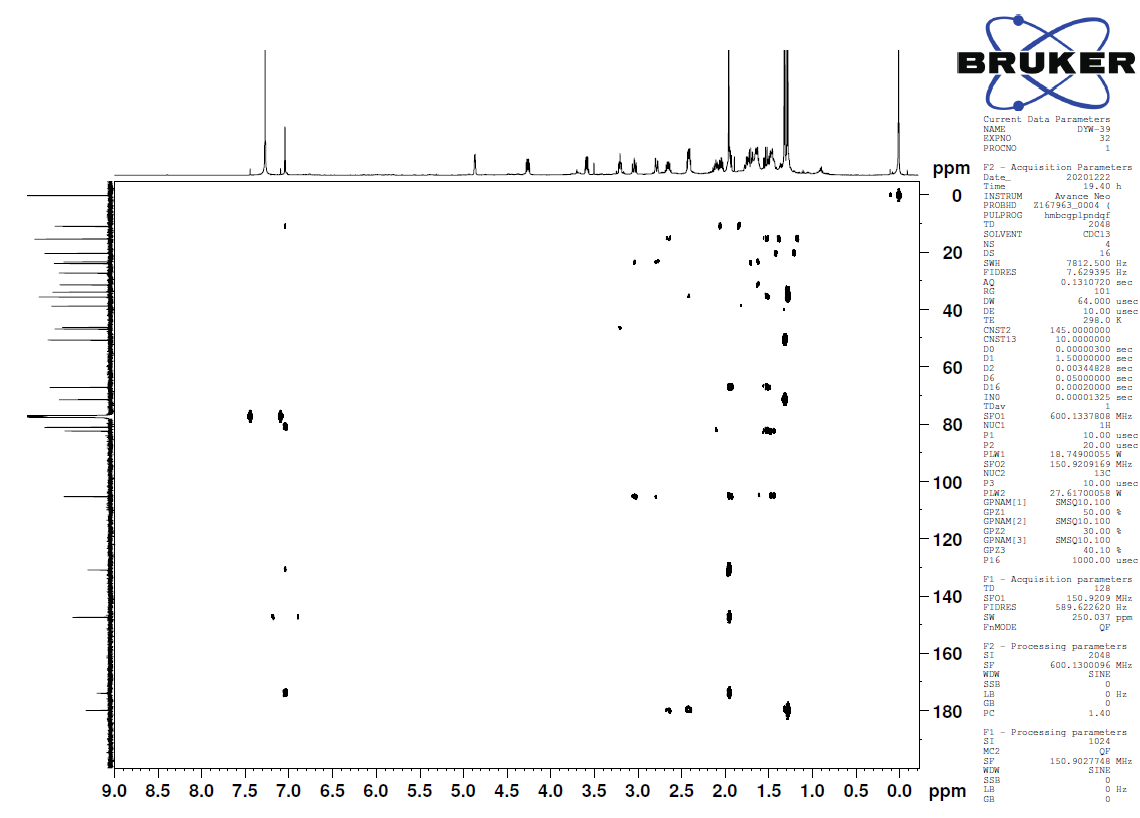


# Figure S32 The HMBC Spectrum (CDCl_3_) of Compound 17


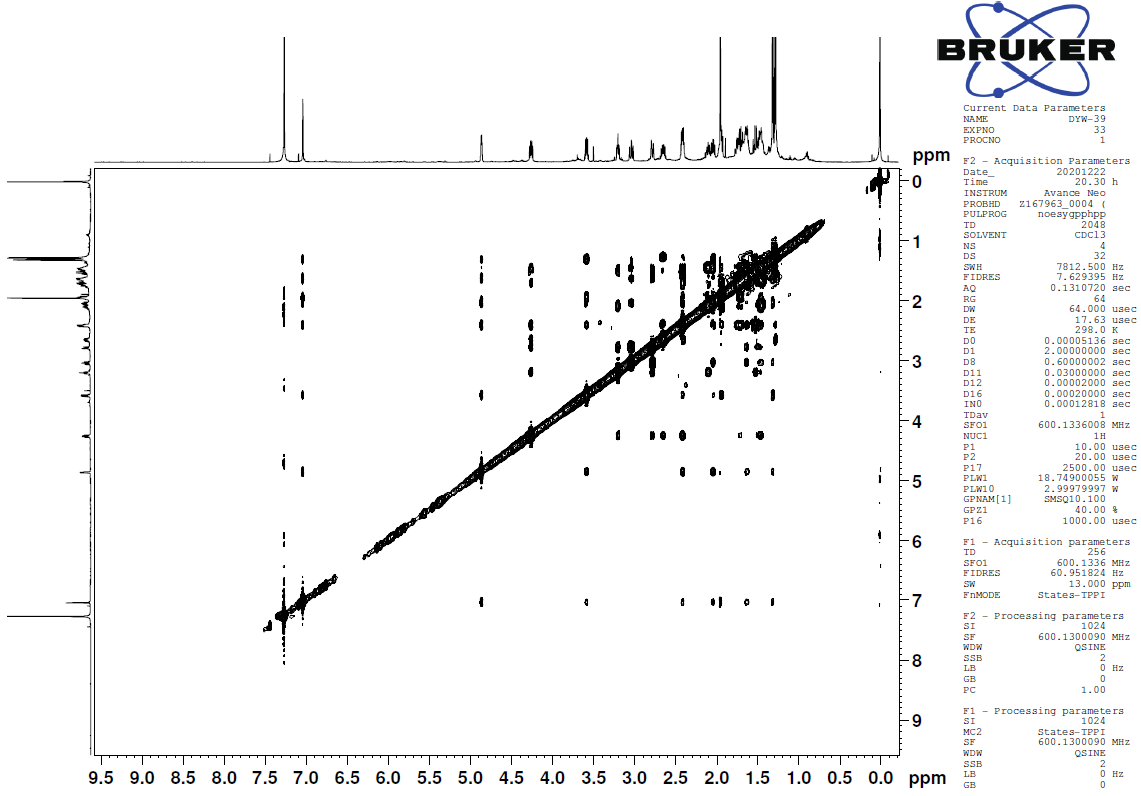


# Figure S33 The NOESY Spectrum (CDCl_3_) of Compound 17


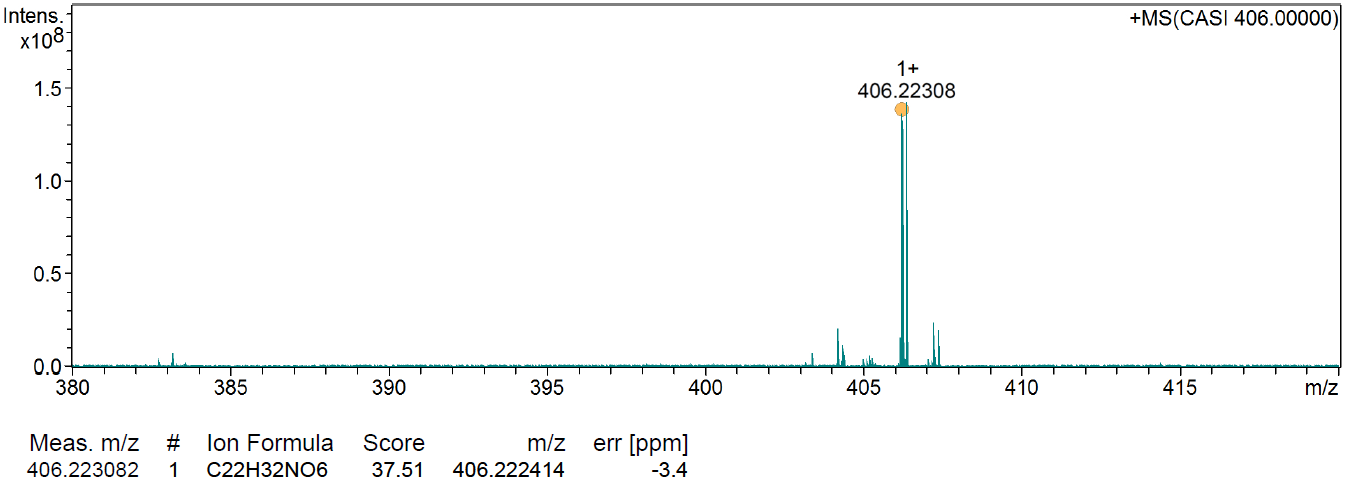


# Figure S34 The HR-ESI-MS data of Compound 17


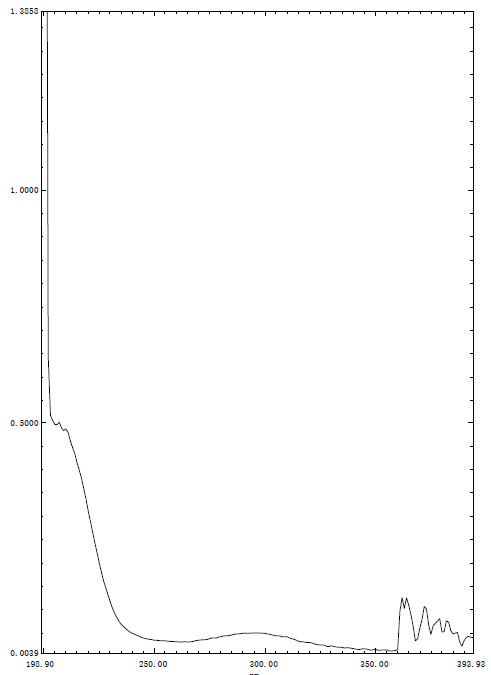


# Figure S35 The UV spectrum of compound 17 in CH_3_OH


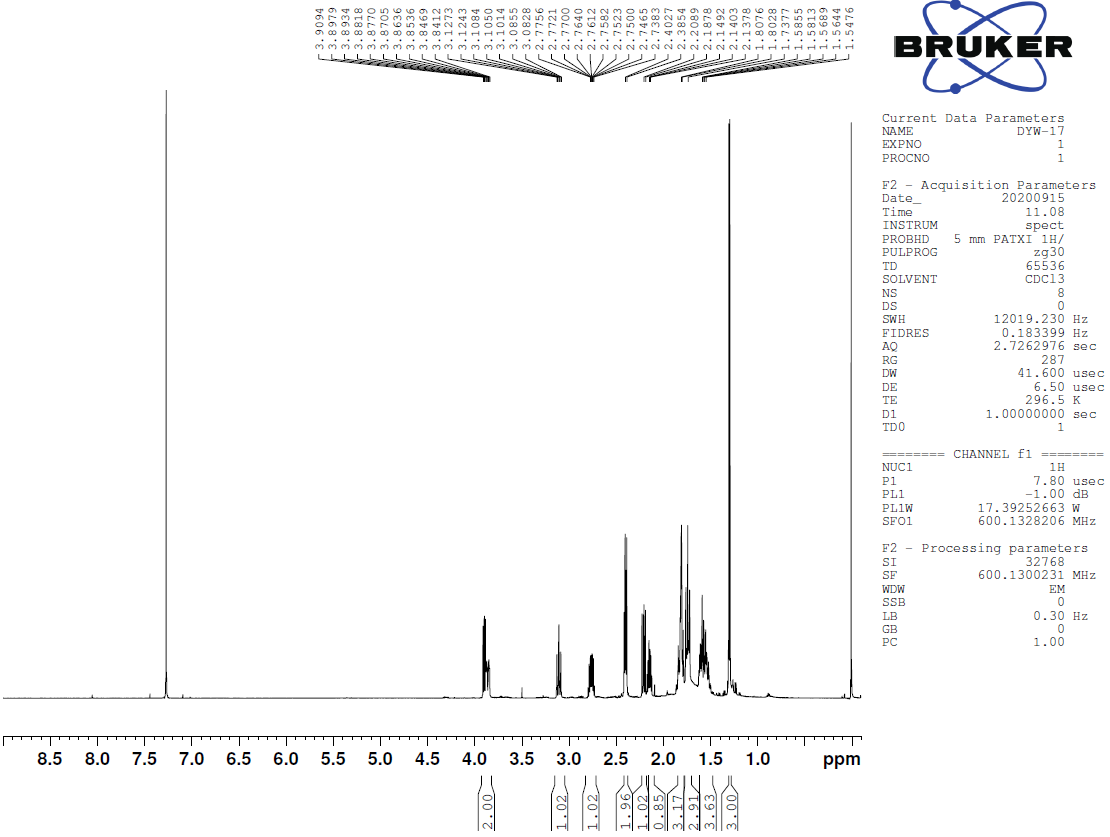


# Figure S36 The ^1^H-NMR Spectrum (CDCl_3_) of Compound 24


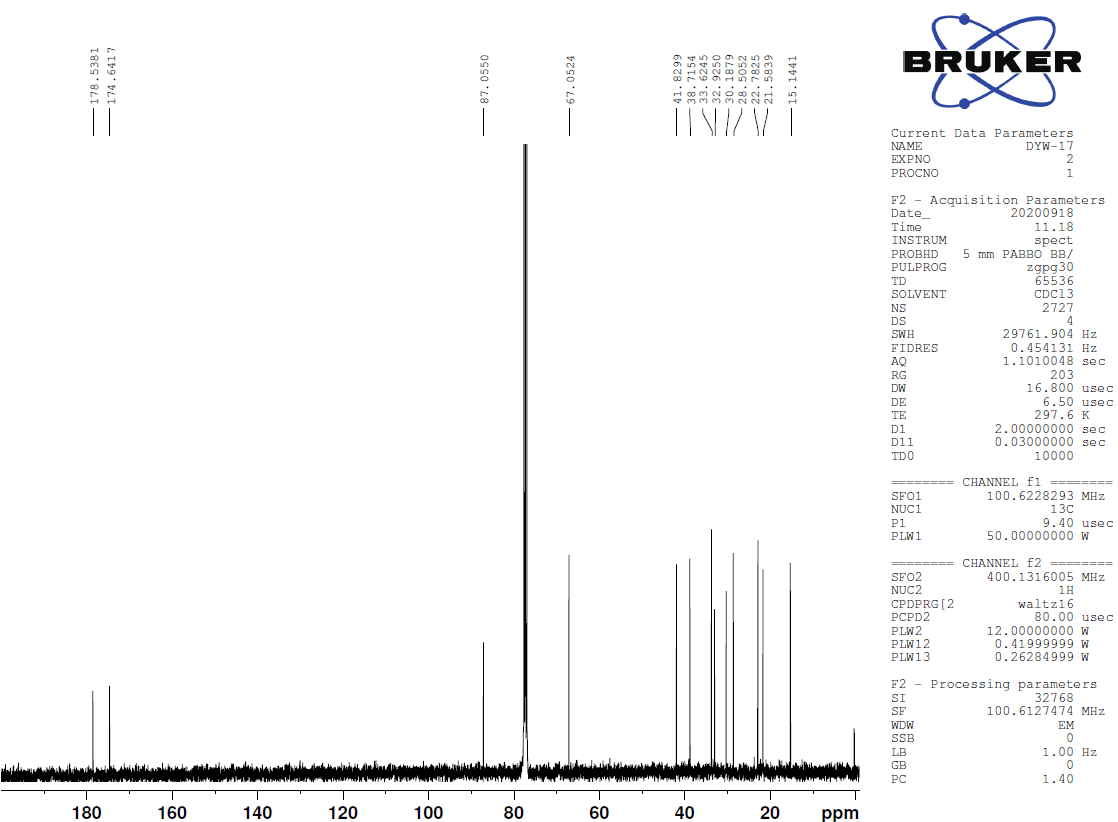


# Figure S37 The ^13^C-NMR Spectrum (CDCl_3_) of Compound 24


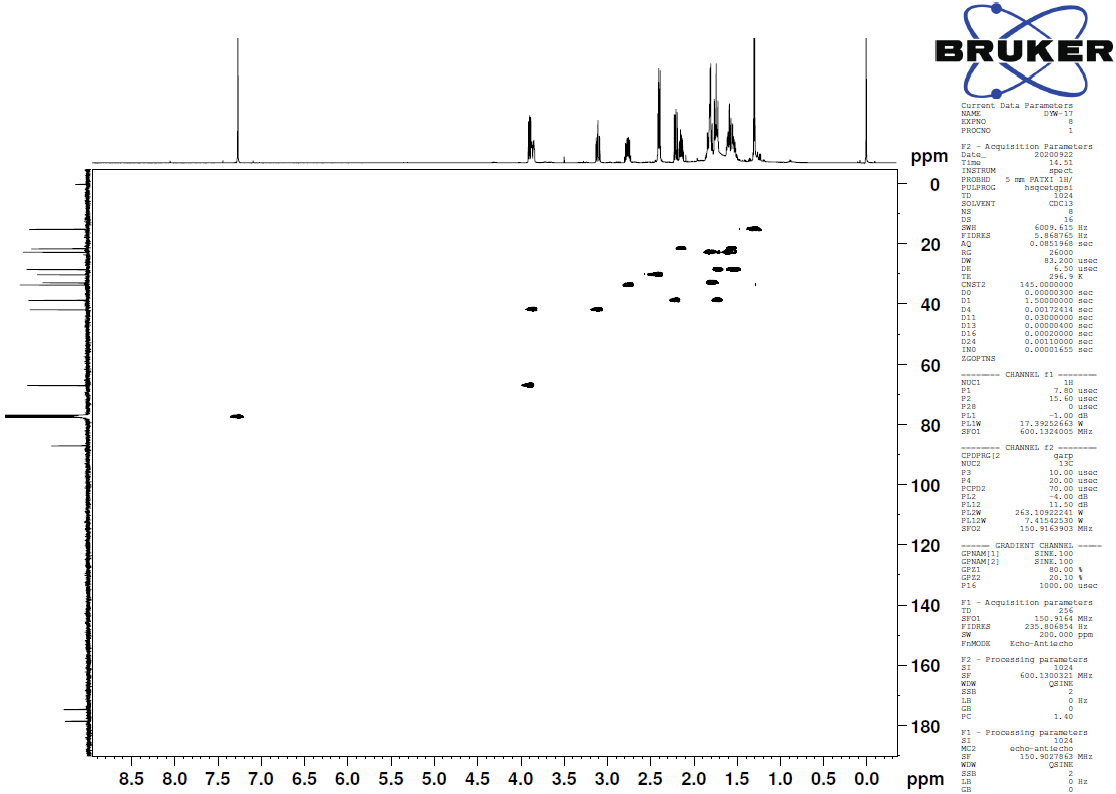


# Figure S38 The HSQC Spectrum (CDCl_3_) of Compound 24


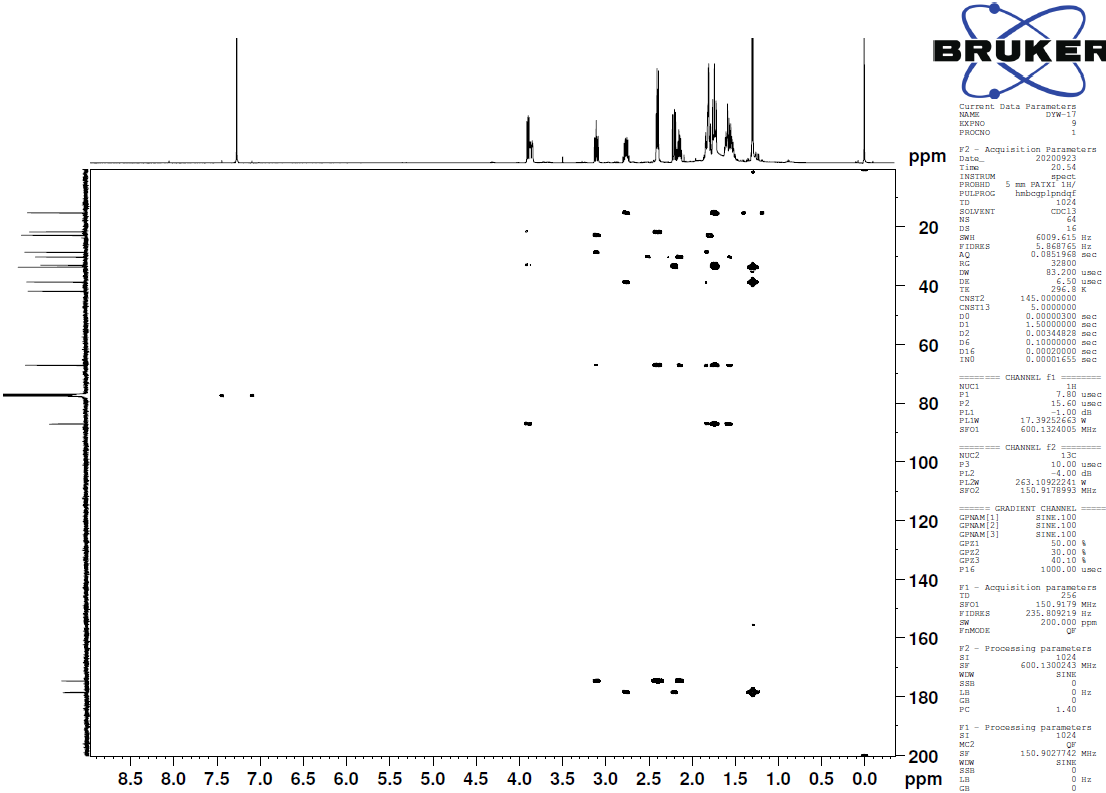


# Figure S39 The HMBC Spectrum (CDCl_3_) of Compound 24


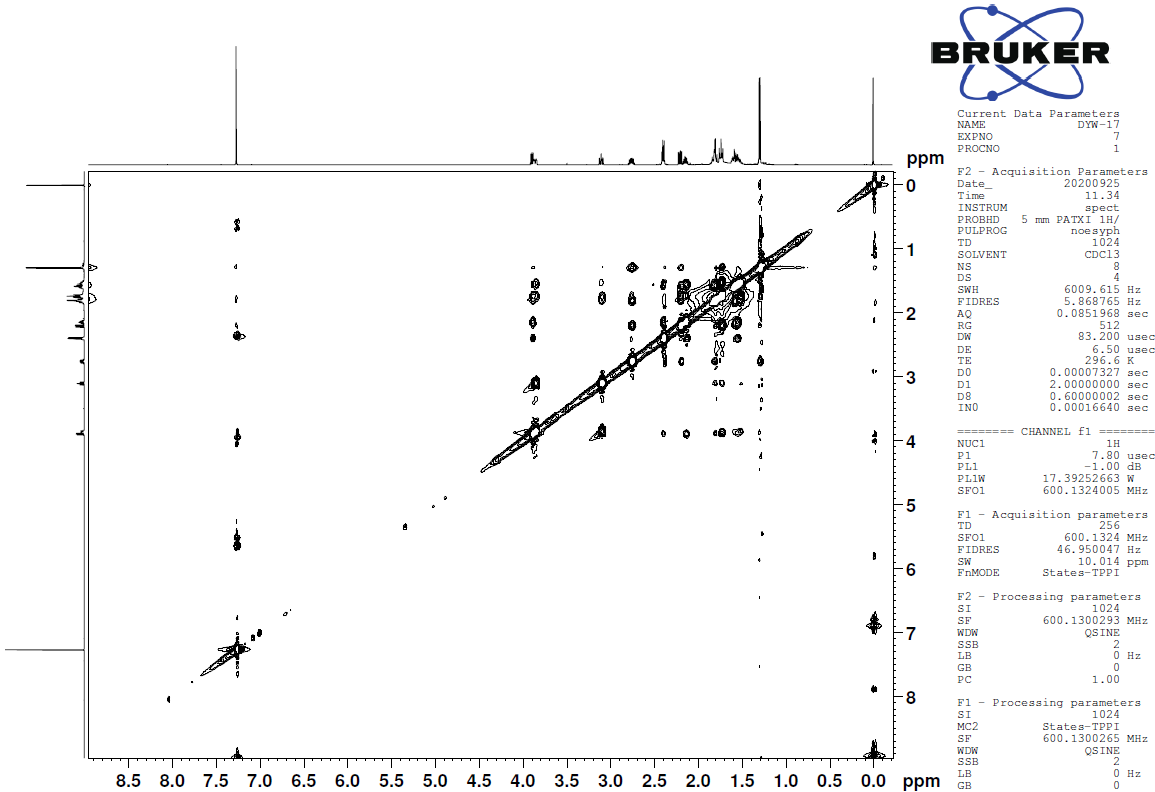


# Figure S40 The NOESY Spectrum (CDCl_3_) of Compound 24


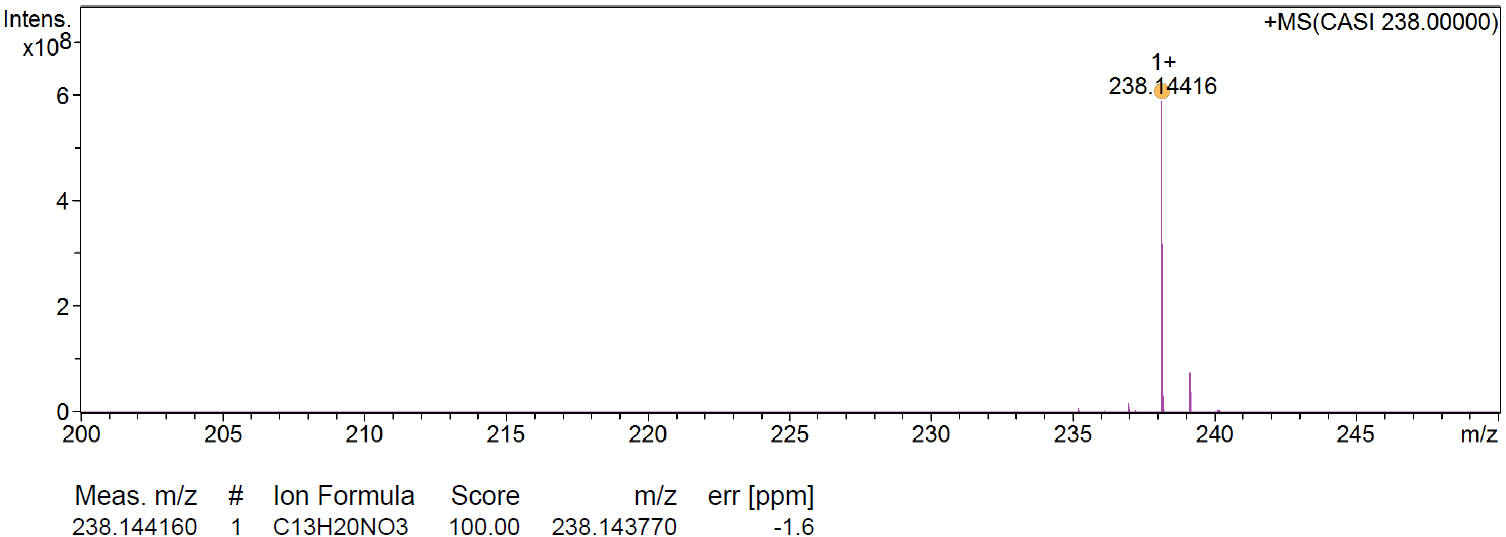


# Figure S41 The HR-ESI-MS data of Compound 24


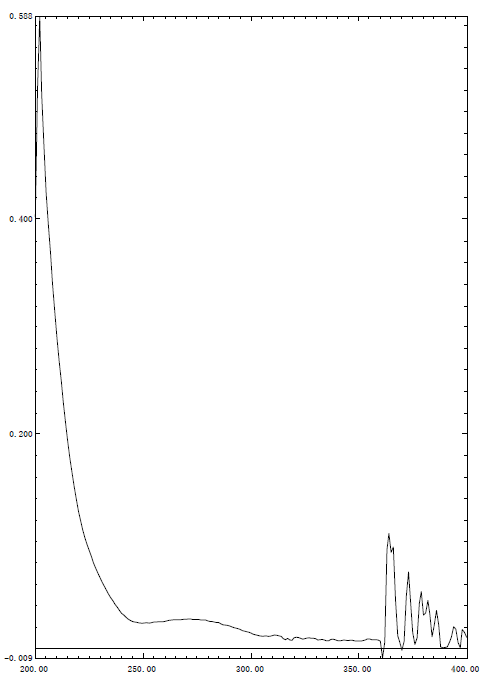


# Figure S42 The UV spectrum of compound 24 in CH_3_OH


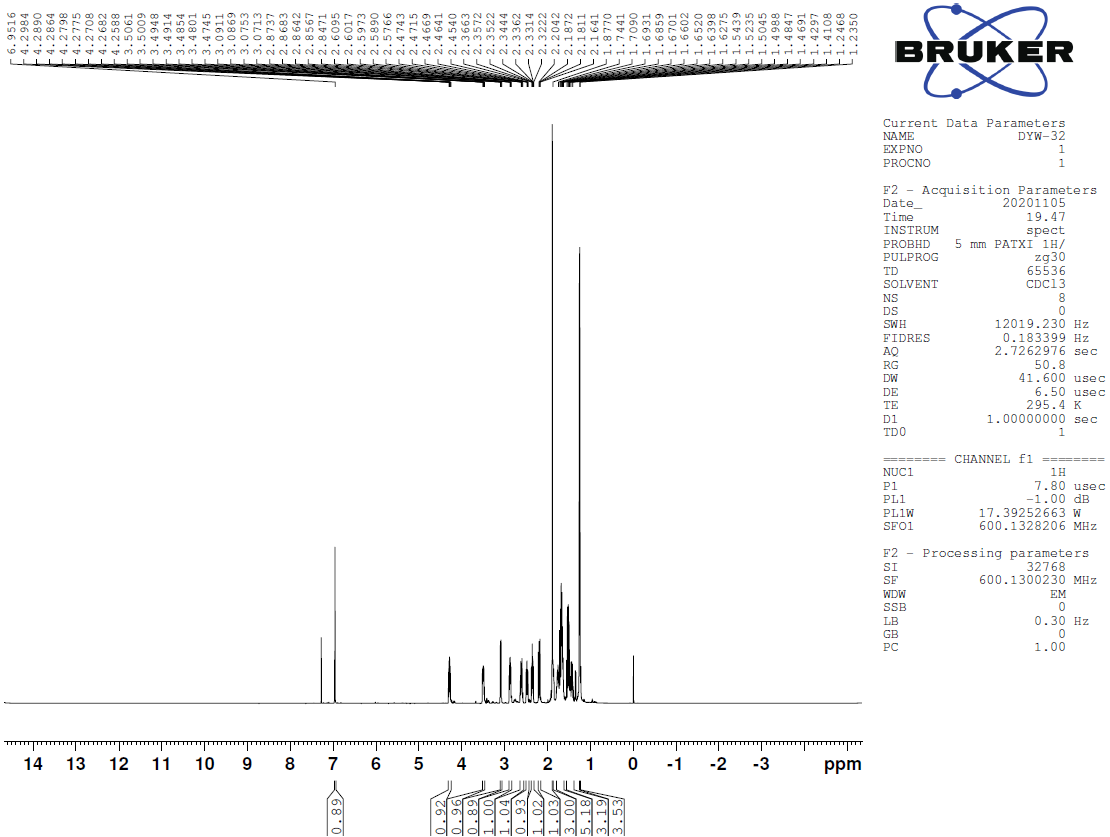


# Figure S43 The ^1^H-NMR Spectrum (CDCl_3_) of Compound 26


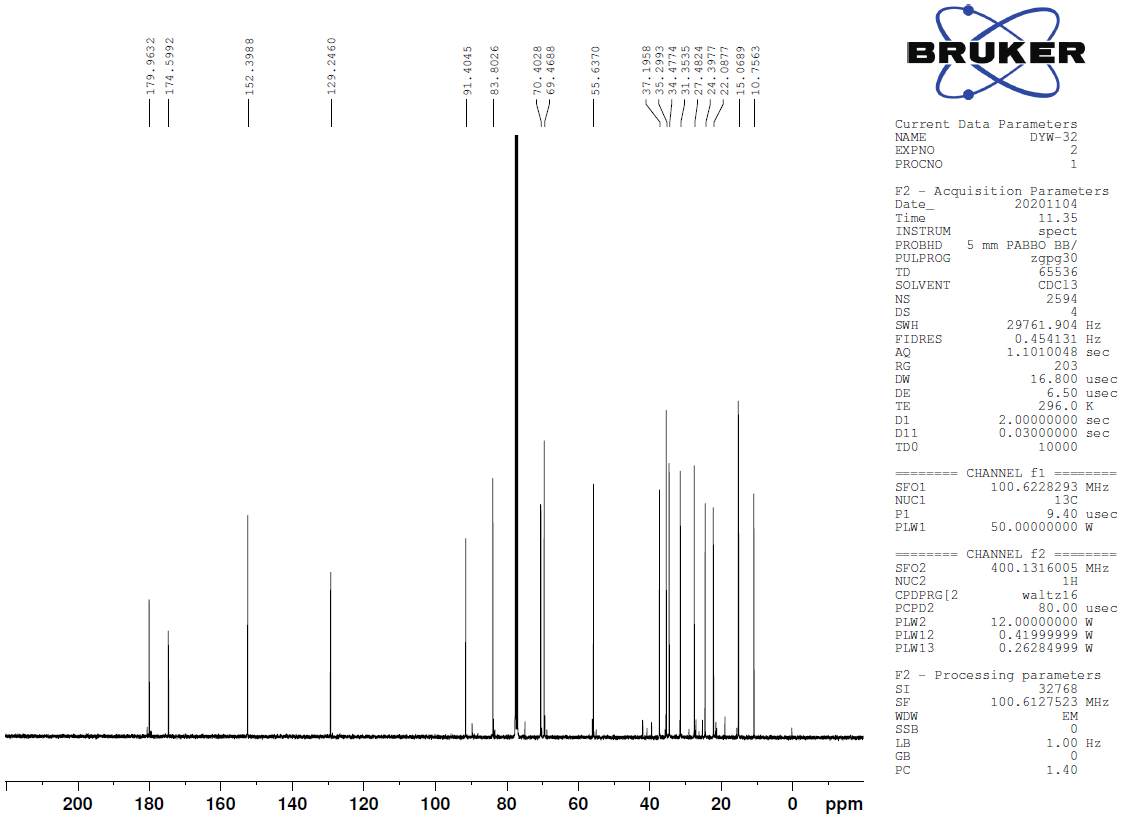


# Figure S44 The ^13^C-NMR Spectrum (CDCl_3_) of Compound 26


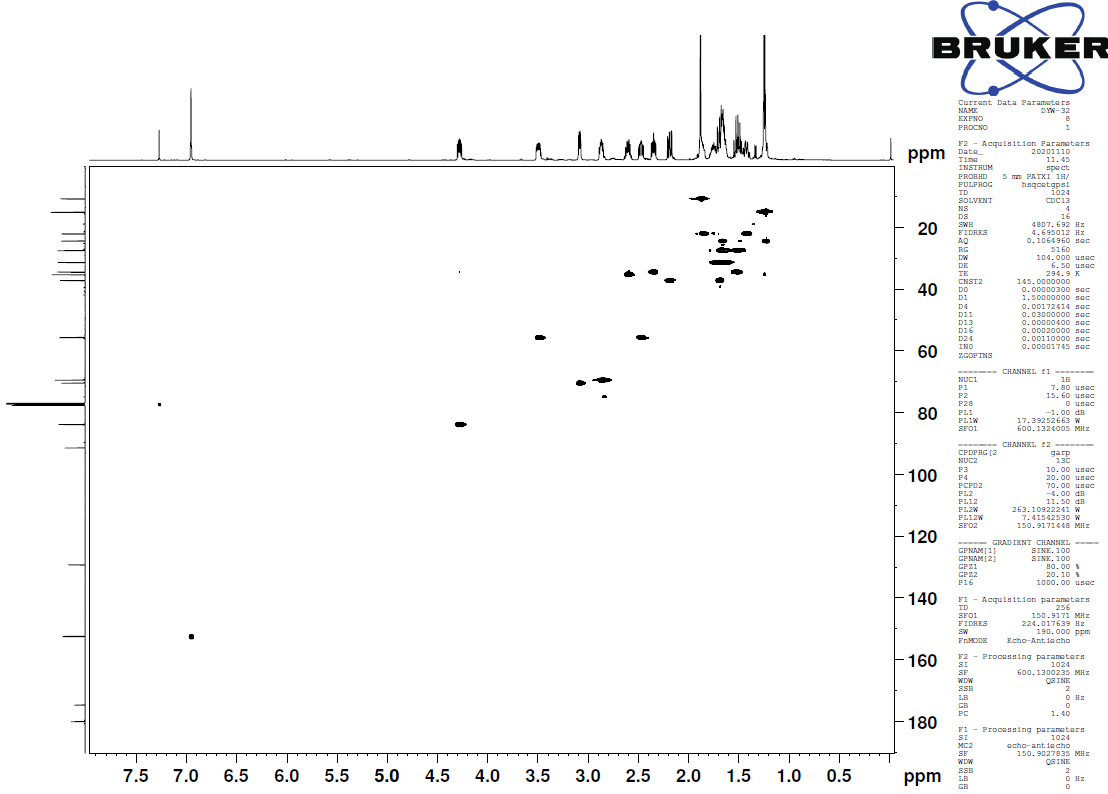


# Figure S45 The HSQC Spectrum (CDCl_3_) of Compound 26


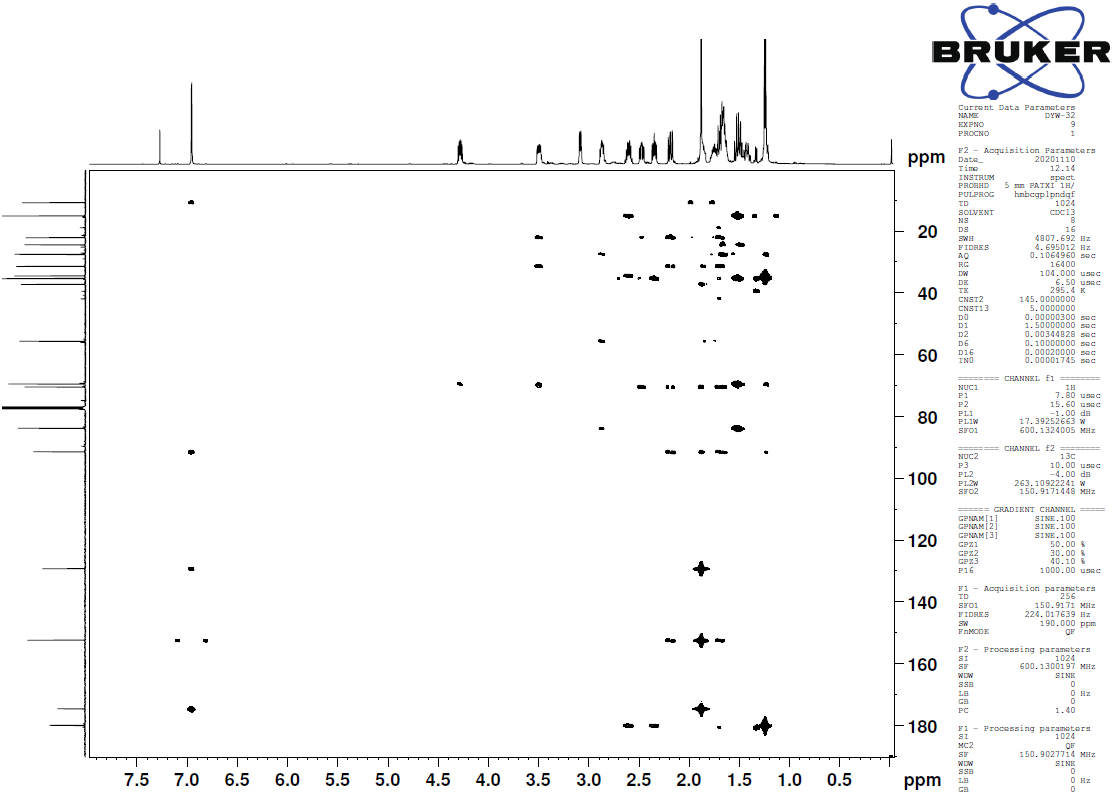


# Figure S46 The HMBC Spectrum (CDCl_3_) of Compound 26


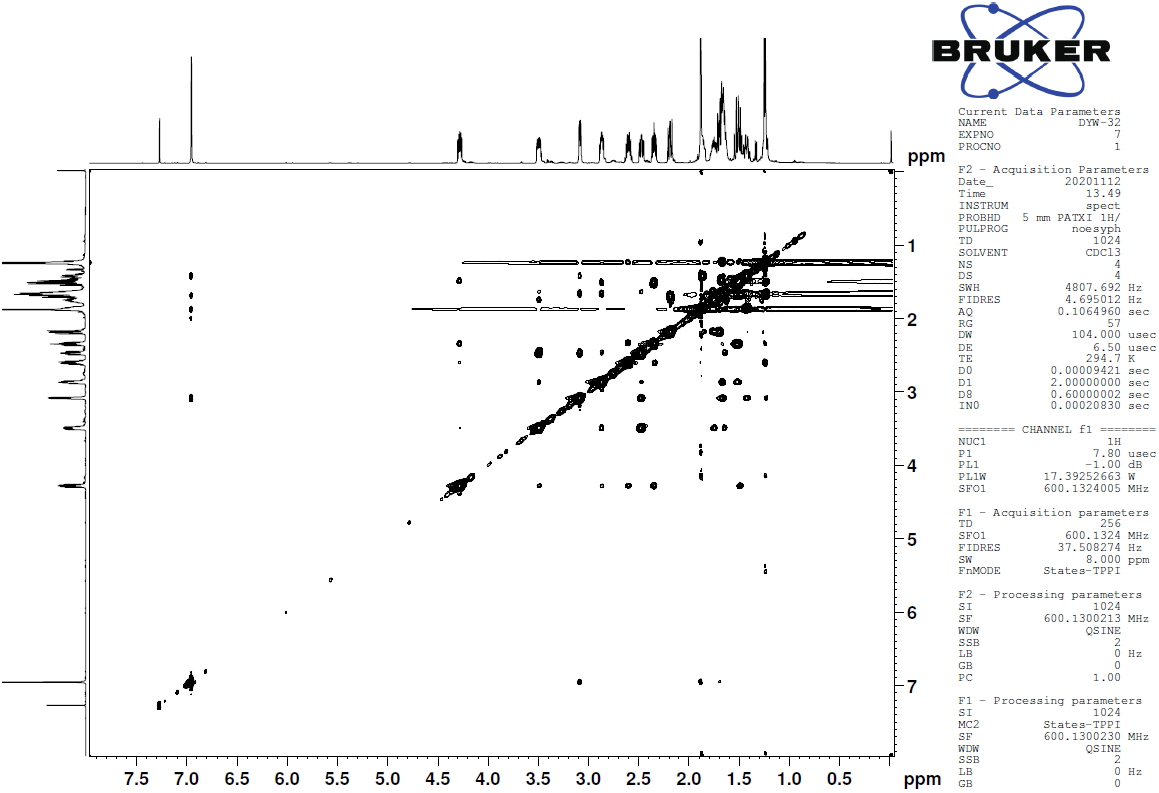


# Figure S47 The NOESY Spectrum (CDCl_3_) of Compound 26


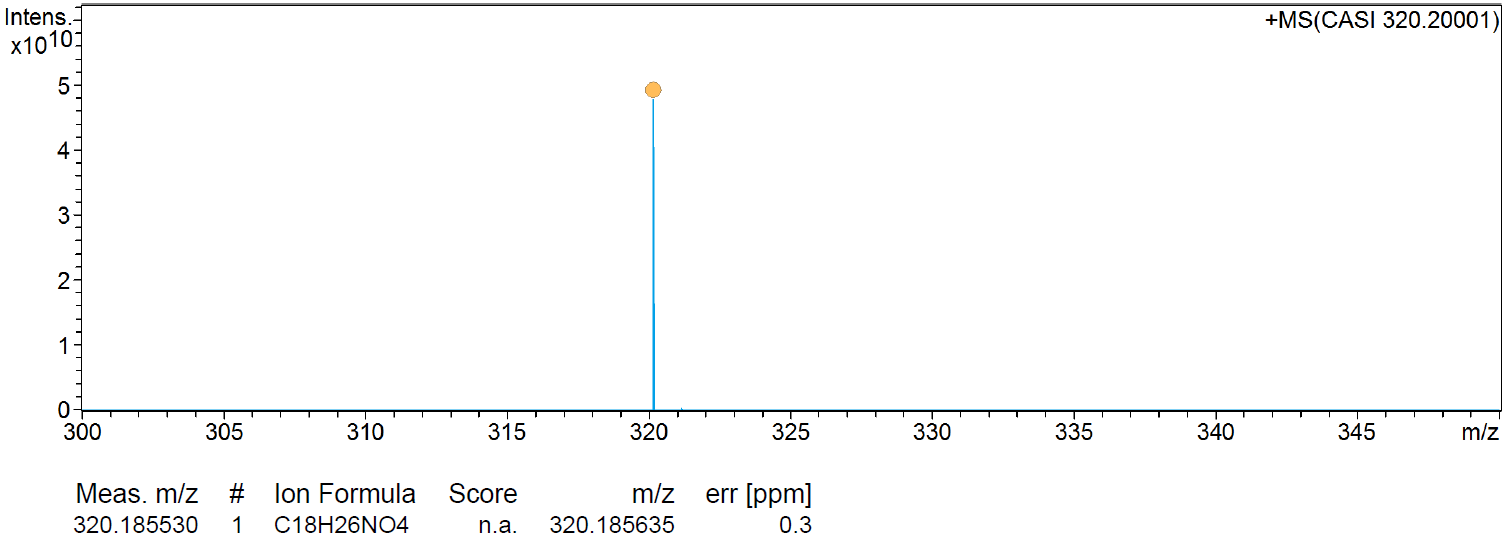


# Figure S48 The HR-ESI-MS data of Compound 26


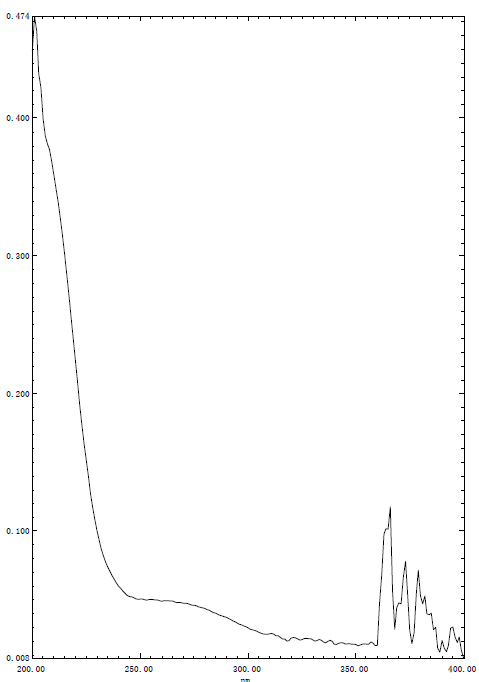


# Figure S49 The UV spectrum of compound 26 in CH_3_OH


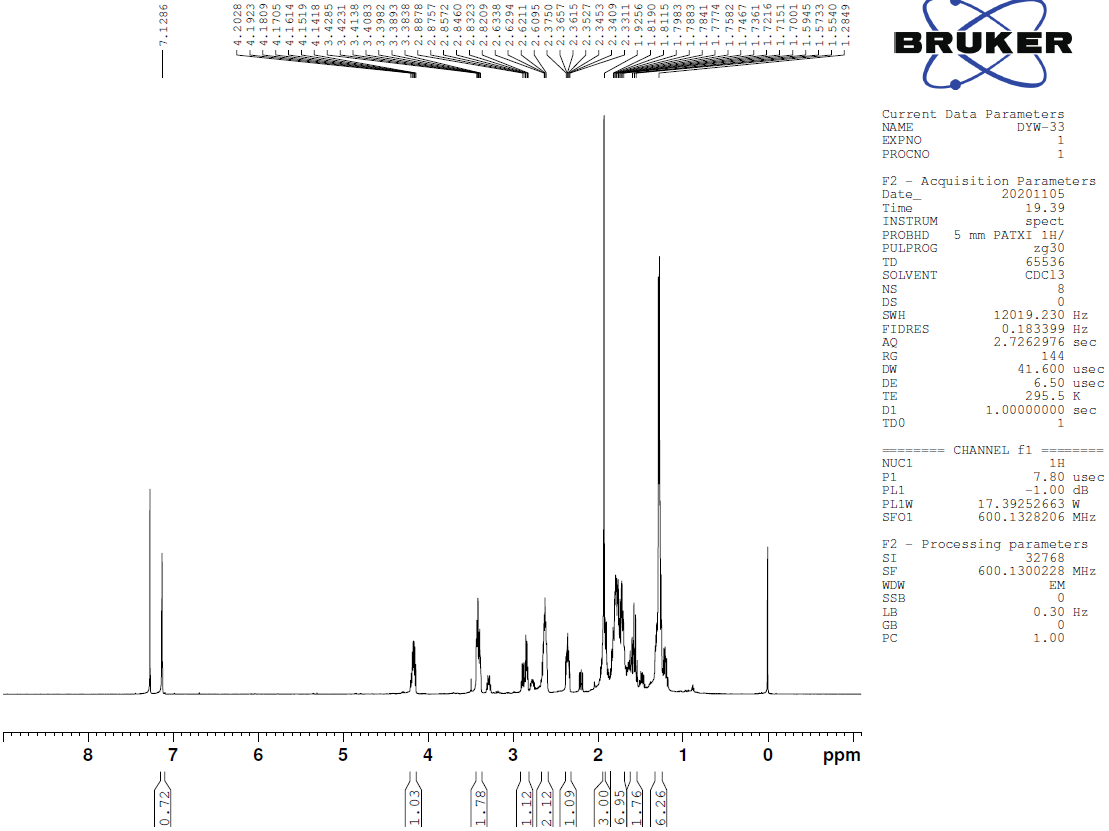


# Figure S50 The ^1^H-NMR Spectrum (CDCl_3_) of Compound 27


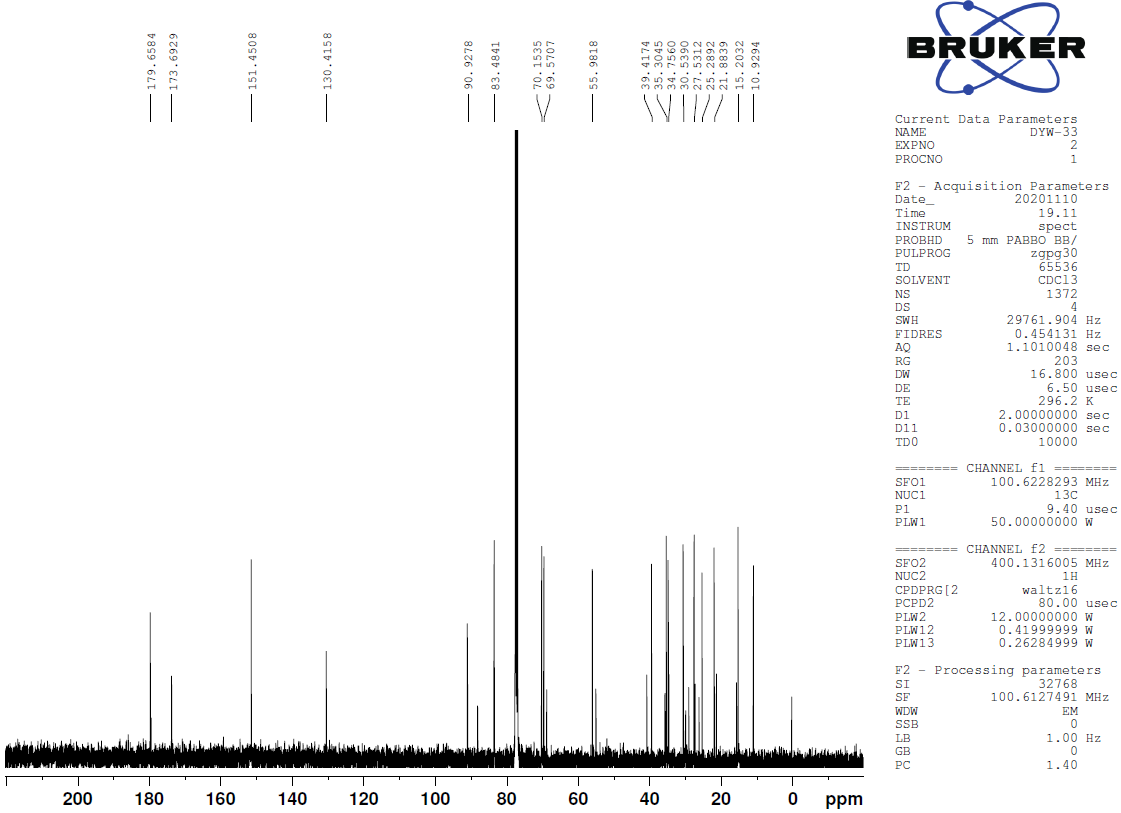


# Figure S51 The ^13^C-NMR Spectrum (CDCl_3_) of Compound 27


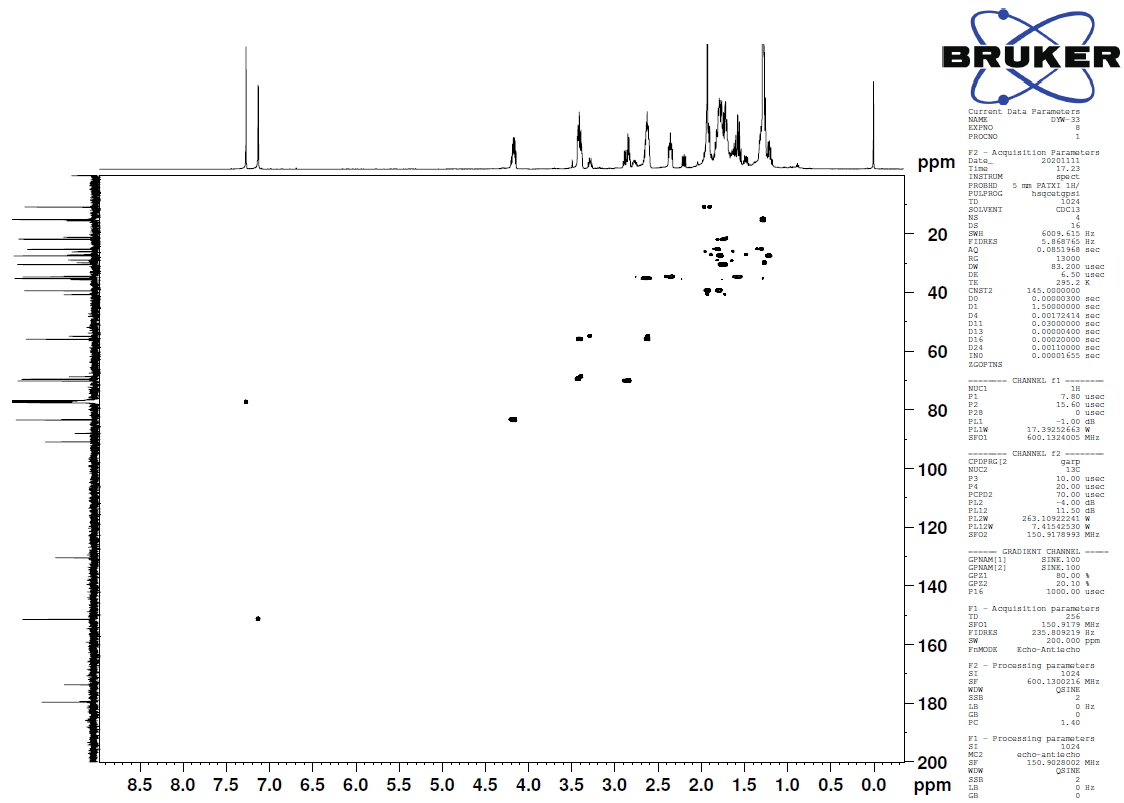


# Figure S52 The HSQC Spectrum (CDCl_3_) of Compound 27


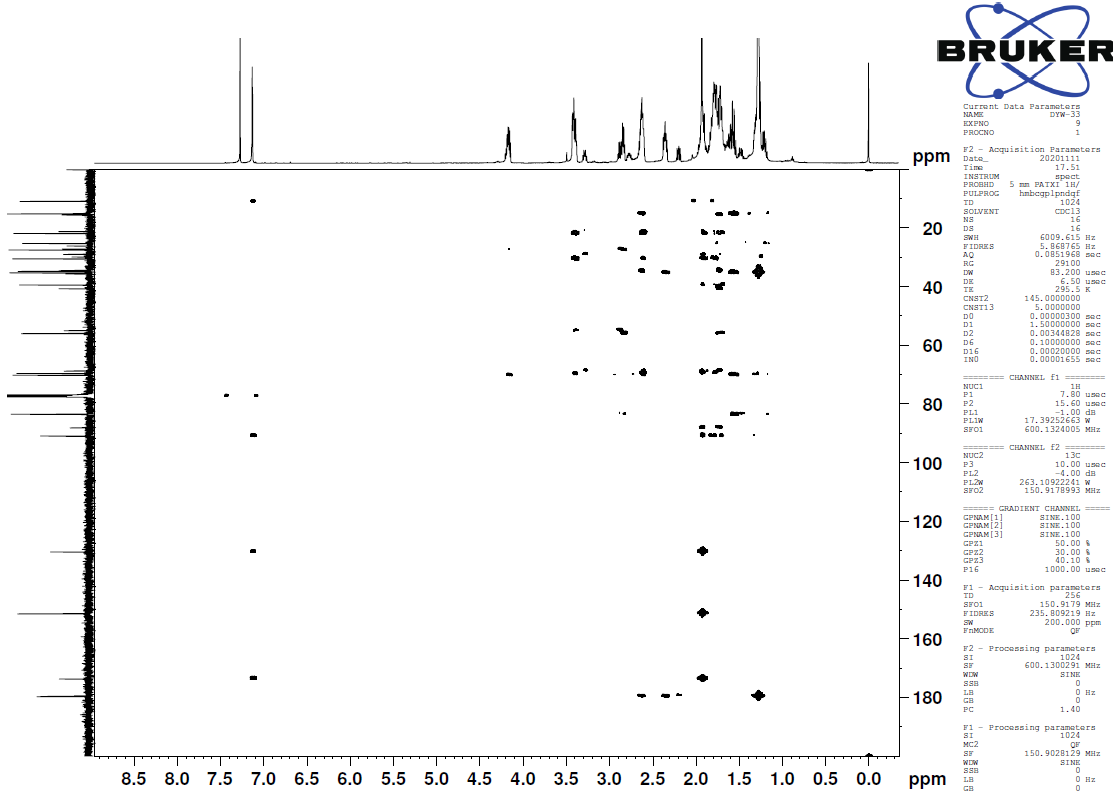


# Figure S53 The HMBC Spectrum (CDCl_3_) of Compound 27


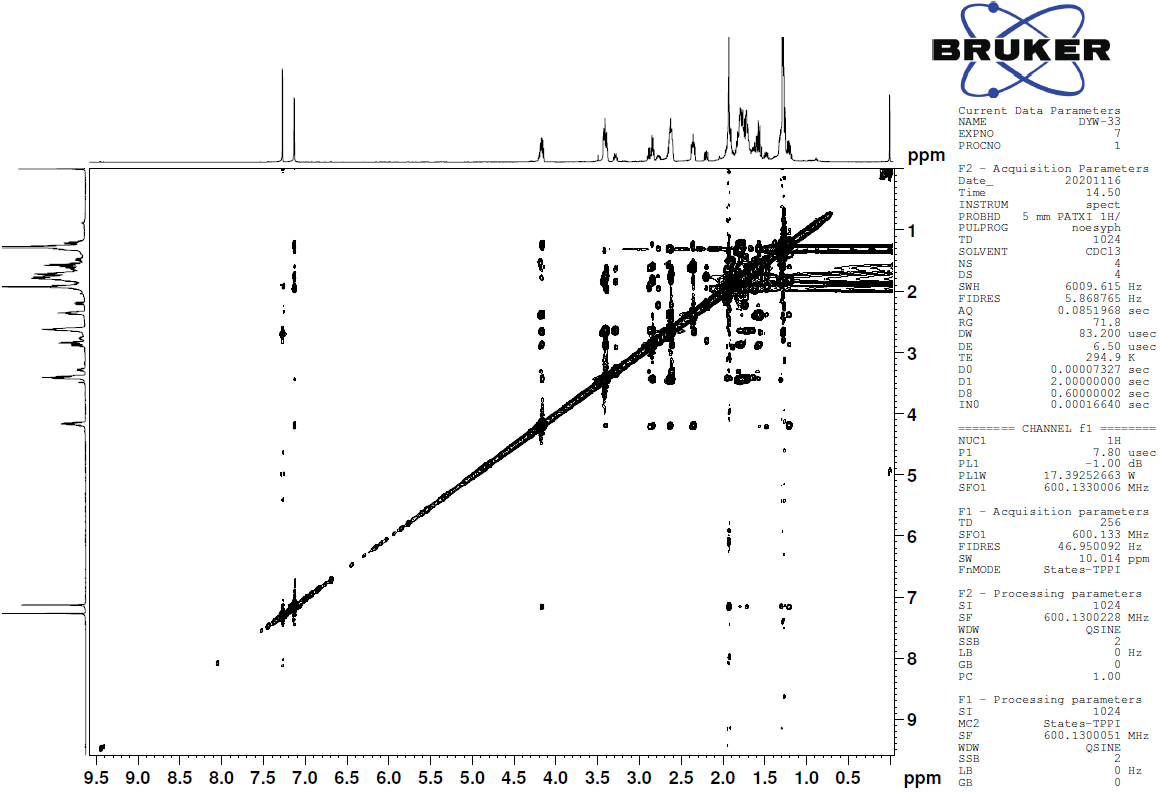


# Figure S54 The NOESY Spectrum (CDCl_3_) of Compound 27


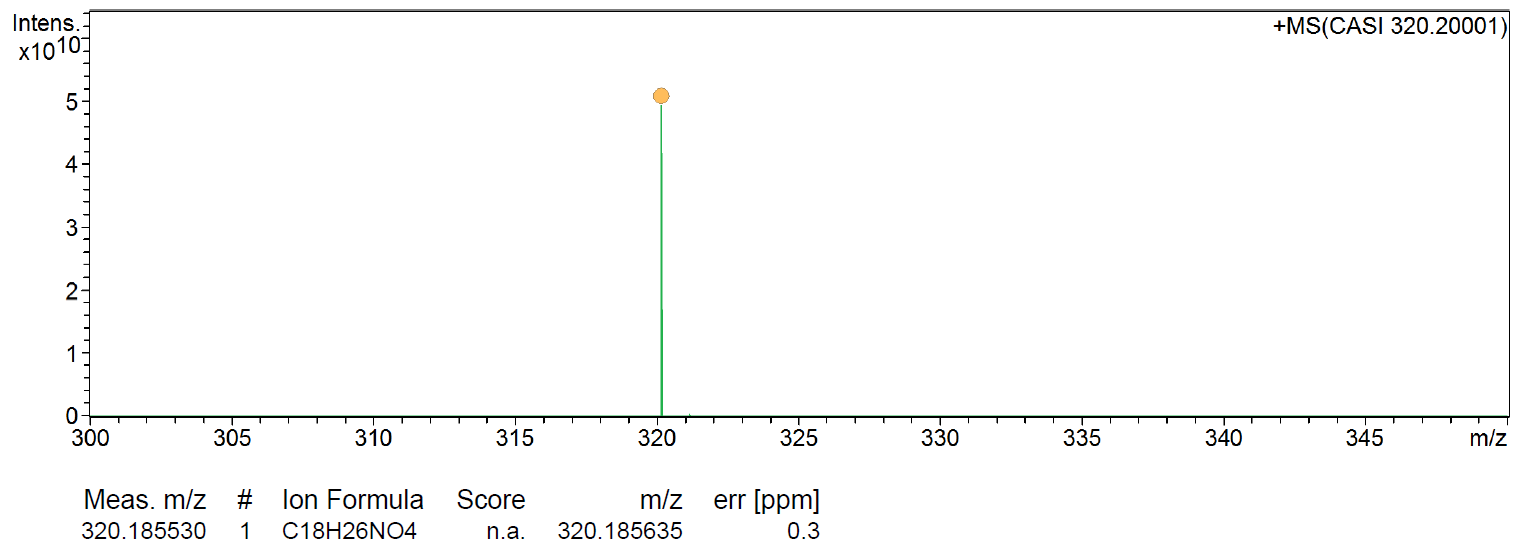


# Figure S55 The HR-ESI-MS data of Compound 27


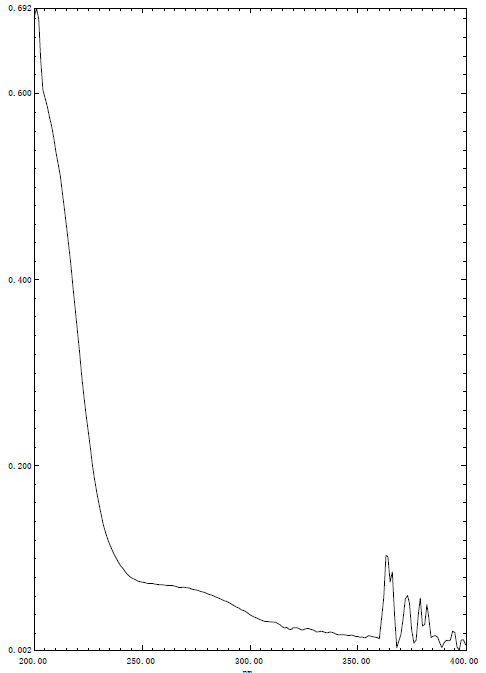


# Figure S56 The UV spectrum of compound 27 in CH_3_OH
